# TORPEdO: A phase III trial of intensity-modulated proton beam therapy versus intensity-modulated radiotherapy for multi-toxicity reduction in oropharyngeal cancer

**DOI:** 10.1016/j.ctro.2022.11.010

**Published:** 2022-11-21

**Authors:** David J. Thomson, Clare Cruickshank, Helen Baines, Russell Banner, Matthew Beasley, Guy Betts, Helen Bulbeck, Frances Charlwood, Judith Christian, Matthew Clarke, Olly Donnelly, Bernadette Foran, Callum Gillies, Clare Griffin, Jarrod J. Homer, Johannes A. Langendijk, Lip Wai Lee, James Lester, Matthew Lowe, Andrew McPartlin, Elizabeth Miles, Christopher Nutting, Nachi Palaniappan, Robin Prestwich, James M. Price, Clare Roberts, Justin Roe, Ramkumar Shanmugasundaram, Rita Simões, Anna Thompson, Catharine West, Lorna Wilson, Jane Wolstenholme, Emma Hall

**Affiliations:** a The Christie NHS Foundation Trust, Manchester, United Kingdom; b The Institute of Cancer Research, London, United Kingdom; c Radiotherapy Trials QA Group (RTTQA), The Leeds Teaching Hospitals NHS Trust, Leeds, United Kingdom; d Swansea Bay University Health Board, Swansea, United Kingdom; e Bristol Cancer Institute, Bristol, United Kingdom; f Manchester University NHS Foundation Trust. Manchester, United Kingdom; g Brainstrust – The Brain Cancer People, Cowes, United Kingdom; h Nottingham University Hospitals NHS Trust, Nottingham, United Kingdom; i Portsmouth Hospitals NHS Trust, Portsmouth, United Kingdom; j Sheffield Teaching Hospitals NHS Foundation Trust, Sheffield, United Kingdom; k University College Hospitals London NHS Foundation Trust, London, United Kingdom; l University Medical Centre Groningen, University of Groningen, Groningen, the Netherlands; m Princess Margaret Hospital, Toronto, Canada; n Radiotherapy Trials QA Group (RTTQA), Mount Vernon Hospital, Northwood, United Kingdom; o The Royal Marsden NHS Foundation Trust, London, United Kingdom; p Velindre NHS Trust, Cardiff, United Kingdom; q The Leeds Teaching Hospitals NHS Trust, Leeds, United Kingdom; r Imperial College, London, United Kingdom; s University Hospital Southampton NHS Foundation Trust, Southampton, United Kingdom; t The University of Manchester, Manchester, United Kingdom; u Health Economics Research Centre, University of Oxford, United Kingdom

## Abstract

• There is a lack of prospective level I evidence for the use of PBT for most adult cancers including oropharyngeal squamous cell carcinoma (OPSCC).
• TORPEdO is the UK’s first PBT clinical trial and aims to determine the benefits of PBT for OPSCC.
• Training and support has been provided before and during the trial to reduce variations of contouring and radiotherapy planning.
• There is a strong translational component within TORPEdO. Imaging and physics data along with blood, tissue collection will inform future studies in refining patient selection for IMPT.

## 1 Introduction/rationale

**Fig. 1:**
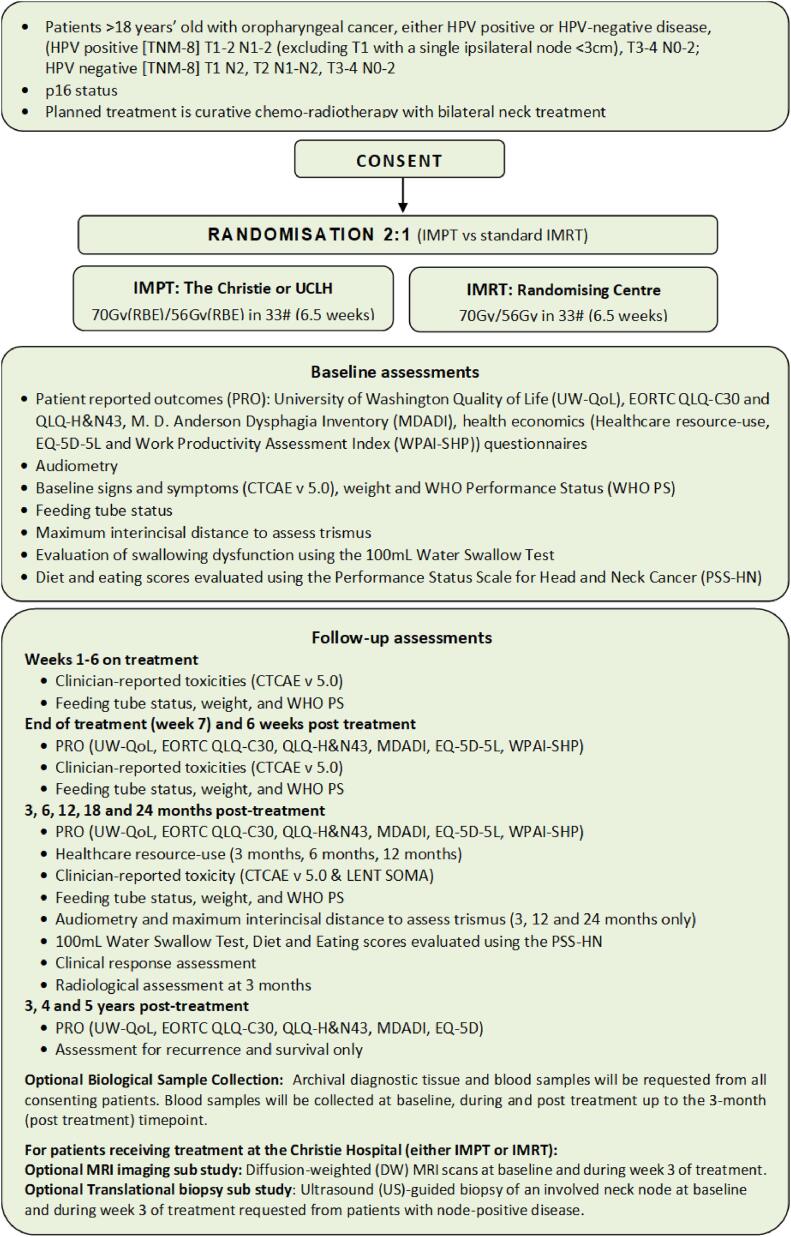
Trial schema.

In 2020, there were an estimated 98,412 cases of oropharyngeal cancer worldwide [1]. Numbers of oropharyngeal squamous cell carcinoma (OPSCC) are rising rapidly due to an increase in HPV-related disease. For locally advanced OPSCC, concurrent chemo-intensity modulated radiotherapy (IMRT) is a standard of care [2]. The treatment of OPSCC with concurrent chemo-radiotherapy is associated with severe acute and late side-effects, which can have a profound detrimental impact on long-term quality-of-life (QoL) [3].Fig. 1.

There is a need to improve treatment related toxicities and health related (HR)-QoL for patients treated with chemo-radiotherapy for OPSCC. Technological advances in radiotherapy delivery, including use of proton beam therapy (PBT), aim to increase sparing of normal tissues with improved functional outcomes and HR-QoL for patients [4], [5].

In the UK, two state-of-the-art PBT facilities are operational; the first (at The Christie NHS Foundation Trust, Manchester) began treating patients in December 2018, and the second (at University College London Hospital, UCLH) in December 2021. Each centre expects to treat up to 750 patients per annum with up to 40 % of capacity for those treated as part of clinical trials.

There is a lack of prospective level I evidence for the use of PBT for most adult cancers [6], including OPSCC. The use of normal tissue complication probability (NTCP) models to select patients for PBT was not considered sufficient justification for the use of PBT in the UK. It was agreed that a high-quality clinical trial of PBT to determine and quantify the benefits of PBT for patients with OPSCC was required to justify the: (i) inconvenience to patients and families who need to travel and stay away from home for a centralised service, (ii) increased treatment costs [7] and (iii) greater resource intensiveness (to account for treatment planning and delivery uncertainties) [8]. The trial also affords valuable opportunity to prospectively evaluate the NTCP models for patient selection, contributing to our understanding of which patients may benefit most from the use of PBT. TORPEdO is the UK’s first PBT clinical trial and aims to determine the benefits of PBT for OPSCC.

## 2 Design

TORPEdO is a phase III multi-centre open-label randomised controlled trial to assess whether intensity modulated proton therapy (IMPT) compared IMRT reduces treatment-related toxicities in patients with locally advanced oropharyngeal squamous cell carcinoma. The trial is registered [ISRCTN: 16424014].

### 2.1 Study objectives

#### 2.1.1 Primary objective

To assess whether IMPT compared with IMRT reduces treatment-related toxicities in patients with locally advanced oropharyngeal squamous cell carcinoma.

#### 2.1.2 Secondary objectives

• To validate a biomarker (normal tissue complication probability (NTCP)-model) as a predictor of benefit from IMPT versus IMRT.
• To estimate the cost-effectiveness of IMPT versus IMRT for OPSCC in the UK
• Define methodology and processes for future UK-led IMPT trials.

#### 2.1.3 Exploratory objectives

• To perform diffusion-weighted magnetic resonance imaging (DW-MRI) at baseline and week three of treatment to determine threshold changes in apparent diffusion co-efficient (ADC) in organs at risk (OARs) that could predict the occurrence of late toxicities.
• To collect biopsy samples at baseline and week three of treatment to evaluate per-treatment changes in the tumour micro-environment for IMRT and IMPT.
• To offer those who decline participation in TORPEdO an opportunity to enrol in a recruitment factors study, to understand barriers to enrolment in UK proton trials.
• To collect samples and treatment planning data for future linked translational research studies.

### 2.2 Patient population

All patients provide written informed consent to participate. Inclusion and exclusion criteria are as follows:

#### 2.2.1 Inclusion criteria

• Histologically confirmed oropharyngeal squamous cell carcinoma
• Human papillomavirus (HPV) positive [TNM version 8] T1-2 N1-2 (excluding T1 with a single ipsilateral node <3 cm), T3-4 N0-2
• HPV negative [TNM version 8] T1 N2, T2 N1-N2, T3-4 N0-2
• Local multi-disciplinary team decision for concurrent chemoradiotherapy with bilateral neck treatment
• Age ≥ 18 years
• WHO performance status 0–1
• Adequate renal function, glomerular filtration rate (GFR) ≥ 60 ml/min calculated using Cockcroft-Gault formula
• Adequate cognitive ability (in the opinion of the local principal investigator (PI) or delegated co-investigator) to complete patient reported outcome (PRO) assessments
• Willingness to comply with the protocol, including travel to the proton centre for IMPT treatment

#### 2.2.2 Exclusion criteria

• Feeding tube insertion required for nutrition (for example due to dysphagia, trismus or low weight/body mass index) prior to treatment [Note: patients who have a feeding tube inserted as part of a planned prophylactic feeding tube approach remain eligible for the study.]
• N3 disease
• Upfront neck dissection
• Use of induction chemotherapy
• Contra-indication to the use of cisplatin for cycle 1 concurrent chemotherapy.
• Previous head and neck radiotherapy
• Major surgery within 6 months of trial entry
• Permanent pacemaker or implantable cardioverter defibrillator
• Any invasive malignancy within previous 2 years (other than non-melanomatous skin carcinoma or cervical carcinoma in situ)
• Previous or concurrent illness (e.g., active infection, symptomatic congestive heart failure, unstable angina pectoris, cardiac arrhythmia, active peptic ulcer disease or gastritis), which in the investigator’s opinion would interfere with completion of therapy, trial assessments or follow up
• Pregnancy, lactating women or women of childbearing potential unwilling or unable to use adequate non-hormonal contraception (male patients should also use contraception if sexually active)
• Pre-existing speech or swallowing problems unrelated to the diagnosis of cancer, which in the local principal investigator’s or delegated co-investigator’s opinion would interfere with completion of therapy, trial assessments or follow up

### 2.3 Treatment allocation

Treatment allocation is by minimisation with a random element to account for imbalances between IMPT and IMRT groups ensuring comparability of clinically important pre-specified prognostic factors (balancing factors) between treatment groups. Balancing factors are; randomising centre, bilateral neck nodes, site of disease, p16 status, smoking status and *T*-stage.

### 2.4 Treatment description

Consenting patients are randomised in a 2:1 ratio to IMPT at a proton centre vs standard IMRT at the recruiting centre. Radiotherapy doses ((when accounting for the greater relative biological effectiveness of PBT) are the same in both treatment groups; a therapeutic dose of 70 Gy (relative biological effective [RBE] equivalent) and an elective dose of 56 Gy (RBE equivalent), both delivered in 33 once-daily fractions over 6.5 weeks using a simultaneous integrated boost technique. An RBE of 1.1 is applied for PBT and 1.0 for IMRT.

Details of the schedule of assessments and follow-up are shown in Table 1.

**Table 1:** Schedule of assessments.

			Treatment Period For IMPT patients ALL assessments during treatment period will be at Proton Beam centre													
ASSESSMENT/VISIT	Screening (Pre randomisation)	Pre-trt (After randomisation)	RT week 1	RT week 2	RT week 3	RT week 4	RT week 5	RT week6	RT week 7	Week 6 post RT	3 months post RT	6 months post RT	12 months post RT	18 months post RT	24 months post RT	Annually at yr 3,4 & 5 post RT
Histological confirmation (p16 by IHC and TMN 8 staging)	X															
Physical examination (Including height)	X															
Patient weight + WHO PS	X		X	X	X	X	X	X	X	X	X	X	X	X	X	
Radiological assessment (MRI/PET-CT/CT thorax)	X^1^										X^2^					
ECG/BSA (concurrent chemotherapy assessments) optional as per local hospital policy	X															
Dental assessment (where possible)*either time point acceptable	X*	X*														
Blood test (FBC, electrolytes, liver function tests)	X		X	X	X	X	X	X	X							
Calculated renal function (Cockcroft Gault Formula/see page 6)	X															
NM isotope GFR (optional, per local policy) *either time point is acceptable	X*	X*														
5-point customised thermoplastic shell and radiotherapy planning CT scan		X														
Repeat radiotherapy planning CT scan					X^4^											
Radiotherapy planning re-plan						X^5^										
Adverse events & toxicities: CTCAE (+LENT SOMA 3-24mths)		X	X	X	X	X	X	X	X	X	X^6^	X^6^	X^6^	X^6^	X^6^	
Clinical response assessment ^7^										X	X	X	X	X	X	X
Audiometry		X^3^									X		X		X	
Maximum interincisal opening MIO to assess for trismus *either time point is acceptable	X*	X*									X		X		X	
Feeding tube status	X		X	X	X	X	X	X	X	X	X	X	X	X	X	
PRO questionnaires^8^ (UW-QOL, EORTC QLQ-C30, QLQ H&N 43, MDADI, EQ-5D-5L, Healthcare resource use^9^ and WPAI-SHP^10^)	X								X	X	X	X	X	X	X	X
Swallowing function assessments (100 ml Water Swallow Test + PSS-HN)	X										X	X	X	X	X	

Translational sub-study (optional)																
EDTA blood (10 ml) for genomics^12^		X^11^														
Streck bloods(40 ml pre-trt; 30 ml thereafter)for liquid biopsies^12^		X^11^				X				X	X					
EDTA bloods (30 ml) for immune markers^12^		X^11^			X			X		X						
EDTA bloods (10 ml) for proteomics		X^11,13^	X	X	X	X	X	X	X		X					
Diagnostic FFPE block request for transcriptomics		X														
**DW-MRI sub-study (optional)^14^**		X			X											
**Biopsy sub-study (optional)^14^**		X			X											

^1^Baseline: MRI neck + CT thorax preferred, however CT neck + CT thorax is acceptable. ^2^At 3 months, PET-CT is preferred, however MRI neck + CT Thorax acceptable (may be performed earlier if clinically indicated). ^3^baseline audiometry should ideally be conducted at randomising centre. ^4^Repeat planning CT scan to be ideally performed on Wednesday of week three. ^5^Re-plan where needed, to start on new plan Monday of week five. ^6^LENT SOMA as well as CTCAE assessments. ^7^Additional tests will be requested if clinically indicated as in standard practice. ^8^PRO booklets will be given to the patient in clinic at screening, end of treatment, 6 weeks post RT, and then sent to the patient’s home address for completion at 3, 6, 12, 18, 24, 36, 48, 60 months. ^9^Healthcare resource use questionnaire only completed at screening, and 3, 6, and 12 months post RT. ^10^ WPAI-SHP questionnaires not completed years 3–5. ^11^Samples will be taken as described in the Sample Collection Manual ^12^ Samples to be collected and sent the same day on Mon-Weds only, and not two days prior to a bank holiday. ^13^Two proteomic samples taken pre-treatment. ^14^Patients recruited by The Christie only.

### 2.5 Radiotherapy quality Assurance (RT QA)

A comprehensive RT QA programme for the TORPEdO trial has been designed and implemented by the National Radiotherapy Trials Quality Assurance (RTTQA) Group including pre-trial and on-trial components. The QA processes for the TORPEdO trial has been streamlined, where possible, with previous head and neck trial QA based on both centre and PI trial participation.

For pre-trial QA centres must complete the following prior to site activation: 1) Facility questionnaire, 2) Benchmark outlining cases, 3) Benchmark planning cases 4) Dosimetry audit (subject to prior RTTQA accreditation).

On trial QA includes: Independent prospective case review of all contouring cases and the first plan from each centre, and retrospective case review of all other plans. Digital Imaging and Communications in Medicine (DICOM) data is collected for all patients. Treatment plan QA is supported by professionals with experience from an independent European proton centre. Radiotherapy planning and delivery guidelines are provided in appendix 2.

### 2.6 Associated research

#### 2.6.1 Health economics study

A within-trial economic component has been integrated into the study to estimate the cost-effectiveness of IMPT from the perspective of the UK NHS, patients and society. Healthcare resource use will be collected from hospital electronic patient records as well as via patient or family member self-reporting using a patient resource-use questionnaire developed specifically for the study. Patient resource use from the date of randomisation to the end of 1-year follow-up will be used to define cumulative healthcare costs alongside within trial responses to the EQ-5D-5L questionnaire for the within-trial economic component.

#### 2.6.2 Imaging and physics based translational study

The collection of all pre-accrual (benchmarking) and during-accrual (recruited patients) DICOM-RT and associated non-DICOM data, will underpin future studies to validate and develop improved NTCP models, refining patient selection for IMPT and explore the following hypotheses:

• Correlation of image-based data mining DICOM data and per-voxel RT doses with continuous toxicity variables (e.g., trismus, audiometry, lymphopenia) will improve dose constraints for normal tissue sub-structures.
• Data from weekly cone beam CT images and the repeat verification CT scan will increase understanding of how often to adapt IMPT and IMRT plans and allow development of meaningful thresholds and a clinical traffic light protocol for adaptation.

#### 2.6.3 Biological sub studies

These sub-studies are co-ordinated by the Manchester Cancer Research Centre at the University of Manchester. TORPEdO provides an opportunity to collect samples and data for translational research to inform the design of future biomarker-driven trials aimed at the optimal selection of IMPT vs IMRT based on not only NTCP models but also tumour and normal tissue genomics. Due to differences in the more complex nature of the DNA damage produced by protons vs photons, the following hypotheses are made:

• Patients with tumours exhibiting a defective DNA damage response profile associated with defects in homologous recombination will benefit more from protons compared to photons.
• There will be some non-overlapping genetic variants that increase the risk of toxicity to protons compared to photons.
• Protons will generate an enhanced immune response compared with photons
• Protons will generate differential DNA methylation patterns compared with photons due to differences in DNA damage and normal tissue irradiation.

### 2.7 Safety reporting

Serious Adverse Events (SAEs) are reported after the commencement of radiotherapy and within 30 days of the last radiotherapy fraction.

### 2.8 Endpoints

The co-primary endpoints measured at 12 months after completion of chemoradiotherapy are:

• University of Washington Quality of Life Questionnaire version 4 (UW-QoL v4.0) physical composite score;and
• gastrostomy dependence or Common Terminology Criteria for Adverse Events (CTCAE) grade 3 wt loss (i.e. ≥ 20 % weight loss from baseline)

A patient-reported outcome measure combining a number of relevant physical toxicity endpoints was chosen as a highly relevant way to test the ability of proton therapy to reduce late toxicity and improve quality of life for patients. This choice was supported by patient focus groups, clinicians and commissioners, as a valid comparator, which, if satisfied would lead to a change in practice.

Gastrostomy dependence is a highly relevant endpoint as long-term dependence has a profound negative impact on quality of life for patients [9]. It is a surrogate for severe swallowing dysfunction and other functional impairments such as problems with chewing, taste disturbance or oral dryness. The potential for physician-bias in tube removal is mitigated by the composite inclusion of grade 3 wt loss, where premature tube removal would likely translate into increased weight loss.

Secondary endpoints include longitudinal pattern of health-related quality of life (assessed using the following questionnaires: UW-QoL, EORTC QLQ-C30, EORTC QLQ-H&N43 and M.D. Anderson Dysphagia Inventory (MDADI)), tube feeding status, weight loss >10 % from baseline at any timepoint after 6 months post-treatment, acute and late severe toxicity (assessed using CTCAE v5.0), clinician-rated swallowing function assess using the 100 ml Water Swallow Test [10] and, Performance Status Scale for Head and Neck Cancer (PSS-HN) [11] (normalcy of diet, place of eating and understandability of speech subscales), hearing loss, maximum interincisal opening (MIO) to assess trismus, resection rates, loco-regional tumour control, overall survival and cost-effectiveness.

### 2.8 Statistical considerations

#### 2.8.1 Hypotheses

The intention of the trial is show superiority of IMPT over IMRT in terms of the patient reported and clinician reported co-primary outcome measures. However, demonstration of a significant treatment effect on either of the co-primary endpoints will be considered sufficient to support a conclusion of effectiveness. The use of 2:1 randomisation aligns with NHS England’s preference to increase access to proton therapy for patients within clinical trials and increases power for secondary and exploratory endpoints relating to IMPT.

The sample size requirements are driven by the clinician reported composite endpoint; thus, this is considered first.

#### 2.8.2 Sample size/power considerations for clinician reported co-primary endpoint

The clinician reported co-primary endpoint is a composite binary endpoint where an “event” is.

• gastrostomy dependence or,
• CTCAE grade 3 wt loss (≥20 % from baseline) at 12 months after completion of chemoradiotherapy.

This endpoint was used in a prospective cohort study [12] including 150 patients treated 1:2 with IMPT or IMRT, matched by patient, tumour and treatment factors. In this study, gastrostomy-tube dependence, or grade 3 wt loss after treatment favoured IMPT with rates of 25 % for IMRT and 8 % for IMPT with an odds ratio of 0.23 (95 % CI: 0.07–0.73; p-value = 0.01). The event rate in the IMRT group is consistent with rates of feeding tube dependence alone at 12 months after treatment reported in a systematic review (range 6.9 % to 29.0 %) [13] and with data published from The Christie (16 %) [14].

The event rate in the IMRT control group is thus assumed to be 25 %. The target treatment effect was selected based on an odds ratio of 0.23 translating to a 7 % event rate in the IMPT group. Using a chi-squared based comparison of proportions, with 2:1 randomisation (IMPT: IMRT), 80 % power, and 2-sided 2.5 % significance, 165 participants (110 IMPT: 55 IMRT) are required to detect a difference of 25 % (IMRT) versus 7 % (IMPT) in the proportion of patients with feeding tube dependence or grade 3 wt loss, at 12 months.

An inflation of 10 % has been applied to allow for non-evaluability (e.g., due to drop-out due to disease recurrence or death before 12 months, non-compliance with the 12-month questionnaire or requirement for tube feeding prior to commencement of chemo-radiotherapy). This gives a target sample size of 183 participants (122 IMPT: 61 IMRT).

#### 2.8.3 Sample size/power considerations for patient reported co-primary endpoint

The patient reported co-primary endpoint is the UW-QoL physical composite score. In a previous cohort of head and neck cancer patients treated with IMRT, mean UW-QoL physical composite score 12 months after treatment completion was 71.2 (SD = 14.05), compared to 89.3 at baseline [15]. An 8-point improvement represents a ‘moderate’ and clinically important increase and is consistent with: 1) an NTCP model-based estimate of the mean difference of 13 points at 12 months [16], [17] and 2) the change in EORTC QLQ-C30 Physical Function of 9 points at 12 months seen between patients treated with volumetric modulated arc therapy (VMAT) and with protons [18], [19]. With 156 participants (104 IMPT: 52 IMRT) an 8-point improvement in the mean UW-QoL physical composite scale (assuming equal SD of 14.05 in each group) can be detected at the two-sided 2.5 % significance level with 86 % power (two-sample *t*-test).

In a contemporary ICR-CTSU managed head and neck cancer radiotherapy trial (DARS) [20] where HR-QoL questionnaires are centrally administered, return rates at 12 months post-treatment are approximately 85 %. Applying a 15 % inflation for non-evaluability gives the target sample size of 183 participants (122 IMPT: 61 IMRT).

#### 2.8.4 Interim analysis and stopping rules

Recruitment will be closely monitored by the Trial Management Group (TMG) with escalation to the independent Trial Steering Committee (TSC) should it fall below 50 % of target.

An Independent Data Monitoring Committee (IDMC) will review the accumulating data at least annually in confidence.

An initial review of the statistical assumptions underlying the power calculations for each of the co-primary endpoints will be undertaken after 25 control arm patients have been followed up for 12 months.

No formal stopping rule based on the primary endpoint is proposed although a pre-planned review will take place when 12-month primary endpoint data are available for half the target sample size (estimated month 32 from start of recruitment). There is no suggestion that IMPT will be less effective than IMRT and given the pattern of recurrence seen in this disease setting, and the relatively short accrual period of the trial, any early stopping rule based on recurrence rates is likely to be based on limited information. To monitor recurrence rates, the number of loco-regional recurrences out of the number of patients who have started trial treatment at that point will be tabulated by treatment group along with a p-value from Fisher’s exact test. These data will be sent to the IDMC, along with further details on the site of loco-regional recurrences reported and the IDMC will use this information along with any other emerging data to advise on early stopping of the trial.

### 2.9 Planned timeline

TORPEdO recruited its first patient on 25th February 2020. There was a three-month hiatus to recruitment from March to May 2020 due to the first wave of the COVID-19 pandemic. As of 5th October 2022, the trial is open in 16 centres and is expected to complete recruitment by October 2023.

## Ethics approval

TORPEdO was approved by the North West – Greater Manchester West Research Ethics Committee (19/NW/0700).

## Declaration of Competing Interest

The authors declare that they have no known competing financial interests or personal relationships that could have appeared to influence the work reported in this paper.
