# Supplementary material for: TORPEdO: A phase III trial of intensity-modulated proton beam therapy versus intensity-modulated radiotherapy for multi-toxicity reduction in oropharyngeal cancer
Source: Clin Transl Radiat Oncol. 2022 Nov 21;38:147–54. doi: 10.1016/j.ctro.2022.11.010 (PMC9702982; doi:10.1016/j.ctro.2022.11.010)
Supplement: Supplementary data 1 [file mmc1.pdf]

A phase III trial of intensity-modulated proton beam therapy versus intensity-modulated radiotherapy for multi-toxicity reduction in oropharyngeal cancer.

PROTOCOL

FINAL Version: 5
Dated: 9th February 2022

Chief Investigator:	Dr David Thomson (The Christie NHS Foundation Trust)
Sponsor:	The Institute of Cancer Research
Approval:	Cancer Research UK: Clinical Research Committee (CRUK CRC)
Funder:	Cancer Research UK
Additional Financial Support:	The Taylor Family Foundation
Coordinating Trials Unit:	ICR Clinical Trials and Statistics Unit (ICR-CTSU)<br>The Institute of Cancer Research

Main REC Reference Number: 19/NW/0700
Protocol Number: ICR-CTSU/2019/10067
CCR Number: CCR5134
ISRCTN: 16424014
CRUK Reference Number: CRUK/18/010
IRAS ID: 268843

The TORPEdO trial has been approved by Cancer Research UK's Clinical Research Committee (CRUK CRC).
The TORPEdO trial is part of the National Institute for Health Research Clinical Research Network Trial Portfolio

This protocol is a controlled document and should not be copied, distributed or reproduced without the written permission of the ICR-CTSU

ADMINISTRATION

Clinical Coordination

Dr David Thomson (Chief Investigator)
The Christie NHS Foundation Trust
Wilmslow Road
Manchester
M20 4BX

Translational Lead

Prof Catharine West
Professor of Radiation Biology, Translational Radiobiology Group
Division of Cancer Sciences
School of Medical Sciences, Faculty of Biology, Medicine and Health
The University of Manchester, Manchester Academic Health Science Centre
The Christie NHS Foundation Trust
Wilmslow Road
Manchester
M20 4BX

Trial Coordination

ICR-CTSU (a UKCRC registered CTU and an NCRI cancer clinical trials unit) is responsible for the central coordination of the trial on behalf of the Sponsor.

ICR Clinical Trials & Statistics Unit (ICR-CTSU)
Division of Clinical Studies
The Institute of Cancer Research
Sir Richard Doll Building
Cotswold Road
Sutton, Surrey SM2 5NG

ICR-CTSU Scientific/Trial Methodology Lead : Emma Hall
Tel: 020 8722 4013
Email: Emma.Hall@icr.ac.uk

ICR-CTSU Clinical Trials Programme Manager: Clare Cruickshank
Tel: 020 8722 4058
Email: Clare.Cruickshank@icr.ac.uk

TORPEdO Trial Manager: Debbie Gardiner
Tel: 020 8722 4057
Email: Deborah.Gardiner@icr.ac.uk

ICR-CTSU Statistician: Abdullahi Omar
Tel: 020 8722 4535
Email: Abdullahi.Omar@icr.ac.uk

ICR-CTSU Principal Statistician: Clare Griffin
Tel: 020 8722 4187
Email: Clare.Griffin@icr.ac.uk

Any questions relating to this protocol should be addressed in the first instance to the TORPEdO Trial Manager within ICR-CTSU:

Email: TORPEdO-icrctsu@icr.ac.uk
General enquiries: 020 8722 4157
Fax: 020 8770 7876

PROTOCOL DEVELOPMENT GROUP

David Thomson	Chief Investigator	The Christie NHS Foundation Trust
Matthew Beasley	Clinical Oncologist	University Hospitals Bristol NHS Foundation Trust
Helen Bulbeck	Founder and the Director of Services and Policy (Patient Involvement Advisor)	Brainstrust
Frances Charlwood	Physicist	The Christie NHS Foundation Trust
Matt Clarke	Physicist	The Christie NHS Foundation Trust
Olly Donnelly	Clinical Oncologist	University Hospital Southampton NHS Foundation Trust
Marie Emson	Clinical Trial Programme Manager	ICR-CTSU, The Institute of Cancer Research, London
Bernadette Foran	Clinical Oncologist	Weston Park Hospital, Sheffield Teaching Hospitals NHS Foundation Trust.
Callum Gillies	Physicist	University College Hospitals London NHS Foundation Trust
James Good	Clinical Oncologist	Queen Elizabeth Hospital, University Hospitals Birmingham NHS Foundation Trust.
Emma Hall	Methodology Lead	ICR-CTSU, The Institute of Cancer Research, London
Johannes Langendijk	Head and Neck Radiation Oncologist	University Medical Centre Groningen
Lip Wai Lee	Clinical Oncologist	The Christie NHS Foundation Trust
Mat Lowe	Physicist	The Christie NHS Foundation Trust
Andrew McPartlin	Clinical Oncologist – DW-MRI substudy lead	The Christie NHS Foundation Trust
Elizabeth Miles	NCRI Radiotherapy Trials QA Group (RTTQA) Co-ordinator	Mount Vernon Hospital
Mercy Ofuya	Statistician (ART-NET)	ICR-CTSU, The Institute of Cancer Research, London
Christopher Nutting	Clinical Oncologist	The Institute of Cancer Research and Royal Marsden NHS Foundation Trust, London
Nachi Palaniappan	Clinical Oncologist	Velindre NHS Trust
Robin Prestwich	Clinical Oncologist	St James's University Hospital, The Leeds Teaching Hospitals NHS Trust.
James Price	Clinical Oncologist	The Christie NHS Foundation Trust
Clare Roberts	Dietician	The Christie NHS Foundation Trust
Justin Roe	Speech and Language Therapy Lead	Royal Marsden NHS Foundation Trust/Imperial College, London.
Joel Smith	Health Economics Lead	Health Economics Research Centre, University of Oxford
Anna Thompson	Clinical Oncologist	UCLH
Catharine West	Translational Research Lead	Translational Radiobiology Group, Division of Cancer Sciences, The University of Manchester
Lorna Wilson	Dietician	The Christie NHS Foundation Trust

Protocol Authorised by:

Name & Role	Signature	Date
Dr David Thomson (Chief Investigator)	David Thomson	09/02/2022
Prof Emma Hall (ICR-CTSU Scientific/Trial Methodology Lead)	E Hall	09/02/2022

This protocol describes the TORPEdO trial and provides information about procedures for entering participants into this trial. The protocol should not be used as a guide for the treatment of patients outside of this trial.

Every care was taken in the preparation of this protocol, but corrections or amendments may be necessary. Protocol amendments will be circulated to participating sites as they occur, but sites entering patients for the first time are advised to contact ICR-CTSU to confirm they have the most recent version.

HISTORY OF CHANGES

PROTOCOL VERSION AND DATE	SUMMARY OF CHANGES
Version 1.0 14th October 2019	Original approved version
Version 2.0 7th July 2020	Main changes relate to:<br>• Removal of exclusion of T2 disease with a single ipsilateral node <3cm.<br>• Prophylactic and reactive feeding tube insertion has been defined in order to provide standardisation across participating sites.<br>• Eligibility criteria has been clarified in relation to Cisplatin requirements for cycle 1 of concurrent chemotherapy.<br>• Participation in other clinical trials now includes wording relating to no co-enrolment into INOVATE study (IRAS 237168).<br>• Screening assessments have been clarified.<br>• Audiometry assessments now include details of specific tests.<br>• AE and SAE section has been corrected. AEs will be collected up to 2 years, SAEs will be collected up to 30 days post completion of radiotherapy.<br>• Addition of T stage as a balancing factor (details added to 17.2).
Version 3.0 12th November 2020	Main changes relate to:<br>• Correction of section numbering in main body of text, following the addition of section 7 in SA01.<br>• Pre-randomisation time point changed to pre-treatment time point to ensure only patients randomised to the study have bloods collected.<br>• Clarification to inclusion criteria that GFR can be 60ml/min or over for inclusion.
Version 4.0 6th July 2021	Main Changes relate to:<br>• Addition of translational biopsy sub-study to section 24.5.<br>• Addition of secondary endpoint relating to feeding tube

	removal.<br>• Addition of section 12.10 and 12.11 to clarify definition of progression events and subsequent follow-up.<br>• Chemotherapy section clarified to permit the switch to carboplatin in cycle 2 due to other severe toxicities.
Version 5.0 9th February 2022	Main changes relate to:<br>• Allowance of calculation for creatinine clearance to be calculated via method other than Cockcroft-Gault if approved by the Chief Investigator.<br>• Clarification foot note on required histology for trial entry added.<br>• Randomisation section updated to reflect change of method centres use to request participant randomisation from telephone to email when ICR-CTSU staff are working remotely due to COVID-19 pandemic.<br>• Update to timing of radiological assessment for screening.<br>• Addition of allowance of remote water swallow test.<br>• Biopsy sub study section updated with new method of storage for one of the biopsy samples.

CONTENTS

1. INTRODUCTION 1
Background 1
Known risks and benefits of proton beam therapy 1
Description of population 3
Study rationale 3
2. TRIAL OBJECTIVES 4
2.1 Primary objective 4
2.2 Secondary objectives 4
2.3 Exploratory objectives 4
3. TRIAL DESIGN 5
4. STUDY ENDPOINTS 5
4.1 Co-primary endpoints 5
4.2 Secondary endpoints 5
5. PATIENT SELECTION & ELIGIBILITY 6
5.1 Number of participants 6
5.2 Source of participants 6
5.3 Inclusion criteria 6
5.4 Exclusion criteria 7
5.5 Life style guidelines 7
6. SCREENING 8
6.1 Screening log 8
6.2 Procedure for obtaining informed consent 8
6.3 Participation in other clinical trials 8
7. FEEDING TUBE INSERTION 9
8. CONCOMITANT CHEMOTHERAPY 9
9. PATIENT PATHWAY PRIOR TO RANDOMISATION. 10
10.RANDOMISATION 10
10.1 Patients allocated to receive proton beam therapy 11
11.PROTON BEAM THERAPY: ACCOMMODATION AND TRAVEL 11
11.1 Accommodation 11
11.2 Travel 11
12.TRIAL ASSESSMENTS 12
12.1 Screening assessments 12
12.2 Pre-treatment assessments 13
12.3 On-treatment assessments 13
12.4 End of treatment assessments (at end of week 7) 13
12.5 Post radiotherapy treatment assessments 14
12.6 Long term follow up 14
12.7 Surgical Outcomes 15
12.8 Discontinuation from treatment 15
12.9 Discontinuation from follow-up 15
12.10 Progression Events 15
12.11 Progression Events, Second Cancers and Collection of Follow-up Data 15
13.TREATMENT 19

13.1 Patient immobilisation and image acquisition 19
13.2 Target outlining and planning 19
13.3 Verification and replanning 19
13.4 Treatment Interruptions 20

14.RADIOTHERAPY QUALITY ASSURANCE 21
15.ASSESSMENT OF SWALLOWING FUNCTION 21

15.1 100mL water swallow test (WST) 21
15.2 Performance Status Scale for Head and Neck Cancer (PSS-HN). 22

16.SAFETY REPORTING 22

16.1 Definitions 22
16.2 Reporting Adverse Events to ICR-CTSU 24
16.3 Reporting of Serious Adverse Events to ICR-CTSU 24
16.4 Review of Serious Adverse Events 24
16.5 Expedited Reporting of Related Unexpected SAEs 25
16.6 Anticipated treatment related SAEs 25
16.7 Follow up of Serious Adverse Events 25
16.8 Annual Safety Reporting 25
16.9 Reporting Pregnancies 26

17.STATISTICAL CONSIDERATIONS 27

17.1 Statistical Design and Sample Size Justification 27
17.2 Treatment Allocation 29
17.3 Endpoint Definitions 29
17.4 Statistical Analysis Plan 31
17.5 Interim Analyses and Stopping Rules 33

18.TRIAL MANAGEMENT 33

18.1 Trial Management Group (TMG) 33
18.2 Trial Steering Committee (TSC) 33
18.3 Independent Data Monitoring Committee (IDMC) 34

19.RESEARCH GOVERNANCE 34

19.1 Sponsor Responsibilities 34
19.2 Participating Site Responsibilities 34

20.TRIAL ADMINISTRATION & LOGISTICS 34

20.1 Site activation 34
20.2 Investigator training 34
20.3 Data Acquisition 35
20.4 Central Data Monitoring 35
20.5 On-Site Monitoring 35
20.6 Completion of the Study and Definition of Study End Date 36
20.7 Archiving 36

21.PATIENT PROTECTION AND ETHICAL CONSIDERATIONS 36

21.1 Risk Assessment and Approvals 36
21.2 Public and Patient Involvement 36
21.3 Ethics Approvals 36
21.4 Trial Conduct 36
21.5 Informed consent 36
21.6 Patient Confidentiality 37

21.7 Data Protection 37
21.8 Liability 37

22.FINANCIAL MATTERS 37
23.PUBLICATION POLICY 37
24.ASSOCIATED STUDIES 38

24.1 Health Economics Study 38
24.2 Imaging and physics based translational study 38
24.3 Biological sub study 38
24.4 Diffusion weighted (DW) MRI substudy 40
24.5 Translational biopsy sub study 41

References 45
A1: NTCP Models 51
A2: Health Economics 54
A3: WHO Performance Status 55
A4: Patient Quality of Life and Swallowing Function Assessments 56
A5: Evaluation of Response 58

TORPEdO TRIAL SUMMARY

Protocol title	**TORPEdO - A phase III trial of intensity-modulated proton beam therapy versus intensity-modulated radiotherapy for multi-toxicity reduction in oropharyngeal cancer.**
Short title	TOxicity Reduction using Proton bEam therapy for Oropharyngeal cancer
Trial population	Men and women aged 18 years and over with newly diagnosed oropharyngeal cancer who require concurrent chemo-radiotherapy including bilateral neck treatment.
Target disease	Oropharynx cancer, both HPV positive and HPV-negative disease<br>HPV positive (TNM-8): T1-2 N1-2 (excluding T1 with a single ipsilateral node <3cm), T3-4 N0-2.<br>HPV negative (TNM-8): T1 N2, T2 N1-N2, T3-4 N0-2<br>N3 disease is excluded
Study objectives	The primary aim is to assess whether intensity modulated proton therapy (IMPT) compared with intensity modulated radiotherapy treatment (IMRT) reduces toxicity in oropharyngeal squamous cell carcinoma.<br><br>The secondary aim is to validate a biomarker (NTCP-model) as a predictor of benefit from IMPT *vs* IMRT.
Study design	Phase III randomised, controlled, multicentre radiation technology trial.
Recruitment target	183 patients
Trial treatment	Consenting patients will be randomised in a 2:1 ratio to IMPT at The Christie or UCLH vs standard IMRT at the recruiting centre.<br><br>Radiotherapy doses will be the same in both treatment groups:<br>• therapeutic dose of 70 Gy (relative biological effective (RBE) equivalent)<br>• elective dose of 56 Gy (RBE equivalent)<br>• delivered in 33 once daily fractions over 6.5 weeks
Chemotherapy	Chemotherapy in the context of this trial is not an investigational medicinal product.<br><br>All patients should be suitable for concurrent platinum chemoradiotherapy. Cisplatin 100 mg/m^2 on day 1 and day 22 of radiotherapy will be mandated for all patients with adequate creatinine clearance (≥60 ml/min calculated by Cockcroft-Gault[+] formula prior to randomisation). If patients subsequently develop renal impairment, ototoxicity or neuropathy, or because of other toxicities (and at the discretion of the treating oncologist PI/co-investigator), carboplatin AUC=5 may be substituted for the second cycle of chemotherapy.<br><br>[+]*Where it is a centre's standard practice to use a different GFR estimation of AUC equivalence (e.g., Wright formula), the TORPEdO Trials Office should be notified and approach approved by the CI.*
Co-primary endpoints	The co-primary endpoints are:<br>• University of Washington Quality of Life Questionnaire (UW-QoL) physical composite score; and<br>• gastrostomy dependence or CTCAE grade 3 weight loss (i.e. ≥ 20% weight loss from baseline)<br>measured at 12 months after completion of chemoradiotherapy.

Secondary endpoints	• Longitudinal pattern of health related quality of life (HR-QoL).<br>• Tube feeding status.<br>• Weight loss >10% from baseline at any timepoint after 6 months post-treatment.<br>• Acute and late severe toxicity.<br>• Swallowing function.<br>• Performance Status Scale for Head and Neck Cancer (PSS-HN).<br>• Hearing loss.<br>• Trismus.<br>• Resection rates.<br>• Loco-regional tumour control.<br>• Overall survival.<br>• Cost-effectiveness.
Trial assessments and follow up	• **Audiometry and trismus**<br>Audiometry, and trismus by change in maximum interincisal distance, at baseline, and at 3, 12 and 24 months post treatment<br>• **Radiological imaging** (MRI/CT/PET-CT)<br>At baseline, 12-14 weeks after completion of therapy, and subsequently as required by the investigator if clinical review raises the suspicion of recurrent disease.<br>• **Evaluation of swallowing function**<br>100ml water swallow test and Performance Status Scale for Head and Neck Cancer (PSS-HN) at baseline, and at 3, 6, 12, 18 and 24 months following completion of study treatment.<br>• **Quality of life assessment**<br>Patient Reported Outcomes (PRO) booklets containing the validated UW-QoL, EORTC QLQ-C30 and QLQ-H&N43, MDADI, Work Productivity Assessment Index (WPAI-SHP) (only until 24 months), EuroQol five-dimensional questionnaire (EQ-5D-5L) and Healthcare resource-use (only until month 12) instruments will be given to the patient in clinic at baseline, at the end of treatment, 6 weeks post-treatment, and then sent to the patient's home address for completion at 3, 6, 12, 18, 24, 36, 48, and 60 months.<br>• **Clinical follow-up is consistent with routine practice**<br>Patients will be assessed weekly during treatment, 6 weeks post-treatment, and at 3, 6, 12, 18 and 24 months post-treatment. For the purposes of the trial, subsequent routine follow-up data will be collected annually in years 3-5.
Imaging and physics-based translational research	(1) Pre-accrual (benchmarking) and during-accrual (recruited participant) DICOM-RT and associated non-DICOM data will be collected to validate and develop improved NTCP models and refine patient selection for IMPT.<br>(2) Image-based data mining of DICOM data is planned to correlate per-voxel radiotherapy doses.
**Optional** biological sample collection	Archival diagnostic tissue and blood samples will be requested from all consenting patients. Blood samples will be collected at baseline, during and post treatment up to the 3-month (post-treatment) timepoint (see Section 24 for further details).
**Optional** MRI imaging sub study	Patients receiving treatment at The Christie Hospital (either IMPT or IMRT) will be asked to have additional diffusion-weighted (DW)-MRI scans at baseline. Some patients may also scanned during week 3 of their treatment. Separate

	patient information will be provided regarding this sub study (see Section 24 for further details).
**Optional** translational biopsy sub study	Patients with node-positive disease treated at The Christie (either IMPT or IMRT) will be asked to have additional research biopsies of an involved neck node at baseline and at week 3 of treatment. Separate patient information will be provided (see Section 24 for further details).

TRIAL SCHEMA

- Patients >18 years' old with oropharyngeal cancer, either HPV positive or HPV-negative disease, (HPV positive [TNM-8] T1-2 N1-2 (excluding T1 with a single ipsilateral node <3cm), T3-4 N0-2; HPV negative [TNM-8] T1 N2, T2 N1-N2, T3-4 N0-2
- p16 status
- Planned treatment is curative chemo-radiotherapy with bilateral neck treatment

CONSENT

↓

RANDOMISATION 2:1 (IMPT vs standard IMRT)

**IMPT: The Christie or UCLH**	**IMRT: Randomising Centre**
70Gy(RBE)/56Gy(RBE) in 33# (6.5 weeks)	70Gy/56Gy in 33# (6.5 weeks)

Baseline assessments

- Patient reported outcomes (PRO): University of Washington Quality of Life (UW-QoL), EORTC QLQ-C30 and QLQ-H&N43, M. D. Anderson Dysphagia Inventory (MDADI), health economics (Healthcare resource-use, EQ-5D-5L and Work Productivity Assessment Index (WPAI-SHP)) questionnaires
- Audiometry
- Baseline signs and symptoms (CTCAE v 5.0), weight and WHO Performance Status (WHO PS)
- Feeding tube status
- Maximum interincisal distance to assess trismus
- Evaluation of swallowing dysfunction using the 100mL Water Swallow Test
- Diet and eating scores evaluated using the Performance Status Scale for Head and Neck Cancer (PSS-HN)

Follow-up assessments

Weeks 1-6 on treatment
- Clinician-reported toxicities (CTCAE v 5.0)
- Feeding tube status, weight, and WHO PS

End of treatment (week 7) and 6 weeks post treatment
- PRO (UW-QoL, EORTC QLQ-C30, QLQ-H&N43, MDADI, EQ-5D-5L, WPAI-SHP)
- Clinician-reported toxicities (CTCAE v 5.0)
- Feeding tube status, weight, and WHO PS

3, 6, 12, 18 and 24 months post-treatment
- PRO (UW-QoL, EORTC QLQ-C30, QLQ-H&N43, MDADI, EQ-5D-5L, WPAI-SHP)
- Healthcare resource-use (3 months, 6 months, 12 months)
- Clinician-reported toxicity (CTCAE v 5.0 & LENT SOMA)
- Feeding tube status, weight, and WHO PS
- Audiometry and maximum interincisal distance to assess trismus (3, 12 and 24 months only)
- 100mL Water Swallow Test, Diet and Eating scores evaluated using the PSS-HN
- Clinical response assessment
- Radiological assessment at 3 months

3, 4 and 5 years post-treatment
- PRO (UW-QoL, EORTC QLQ-C30, QLQ-H&N43, MDADI, EQ-5D)
- Assessment for recurrence and survival only

Optional Biological Sample Collection: Archival diagnostic tissue and blood samples will be requested from all consenting patients. Blood samples will be collected at baseline, during and post treatment up to the 3-month (post treatment) timepoint.

For patients receiving treatment at the Christie Hospital (either IMPT or IMRT):
Optional MRI imaging sub study: Diffusion-weighted (DW) MRI scans at baseline and during week 3 of treatment.
Optional Translational biopsy sub study: Ultrasound (US)-guided biopsy of an involved neck node at baseline

1. INTRODUCTION

Background

Oropharyngeal cancer

In 2016 there were over 3000 new diagnoses of oropharyngeal cancers in England (1). Numbers are rising rapidly, with the age-standardised UK incidence approximately doubling from 2.1 to 4.1 between 2002 and 2011 (2). The rising incidence is due to an increase in HPV-related disease, which affects a younger population (mean age 57 *vs* 61 years) (2) and has a better prognosis than HPV negative disease with a 58% reduction in the risk of death (3). For locally advanced oropharyngeal cancer, organ preservation using concurrent chemo-intensity modulated radiotherapy (IMRT) is the standard of care (4). Transoral resection of oropharyngeal tumours and post-operative (chemo)radiotherapy is considered another option, although generally restricted to T1-T2 tumours in the absence of parapharyngeal or midline extension (4).

Clinical need to reduce treatment related toxicities

Over 60% of patients treated with concurrent chemo-IMRT for oropharyngeal cancer experience grade 3 acute side effects (5). Acute toxicities include fatigue, mucositis, pain, taste disturbance, reduced oral intake, dysphagia, aspiration pneumonia, requirement for tube feeding and hospital admissions, which can result in treatment gaps and poor chemotherapy compliance. Acute toxicities can be a precursor to late effects which adversely impact quality of life (QoL) (6, 7). The RTOG retrospective analysis of three concurrent chemo-radiotherapy trials using conventional radiotherapy techniques reported that 43% of 230 patients developed ≥grade 3 late toxicities (8) In a multi-institutional analysis of 1,238 patients treated with concurrent chemo-IMRT, the 1-year rate of gastrostomy dependence was 9% (9). In a systematic review the average rate was 18% (10), and in data published from the Christie the rate was 16% (11). Long-term gastrostomy dependence has profound negative impact on quality of life for patients and is a surrogate for severe swallowing dysfunction and other functional impairments such as problems with chewing, taste disturbance or oral dryness (7). For 201 oropharyngeal cancer patients treated in Manchester and Leeds between 2011 and 2013, there were poor long term swallowing outcomes (composite M. D. Anderson Dysphagia Inventory [MDADI] scores <60) in 32% of patients at least two years following treatment with concurrent chemo-IMRT (12). The Royal Marsden Hospital reported a substantial and statistically significant decrease in University of Washington QoL (UW-QoL) (13) physical functioning composite scores in 61 head and neck cancer patients treated with chemo-IMRT from pre-treatment to 3 and 6 months, followed by only slight recovery at 12 months. A multi-institutional meta-analysis of 1,366 patients from 25 studies and 12 countries using the EORTC QLQ Head and Neck-35 (n=704), UW-QoL (n=474) and MDADI (n=381) questionnaires showed large deteriorations in QoL for multiple physical symptoms at least 12 months following treatment, which corresponded with moderate to severe changes in physical, emotional and global QoL (14). These data strongly support the need for new treatments with fewer side effects.

Known risks and benefits of proton beam therapy

Advanced radiation technologies for oropharyngeal cancer

There is a need to improve treatment related toxicities and health related (HR)-QoL for patients treated with chemo-radiotherapy for oropharyngeal cancer. Technological advances in radiotherapy delivery aim to increase sparing of normal tissues, to realise improved functional outcomes and HR-QoL for patients, and potentially exploit dosimetric-toxicity benefits for dose escalation strategies to improve local control and survival outcomes. The practice changing PARSPORT study (CRUK/03/005) provided level I evidence for the benefit of contralateral parotid-sparing IMRT *vs* conventional radiotherapy in head and neck cancer, showing a significant reduction in ≥grade 2 oral dryness at 12 months with IMRT (38% (95% CI: 23-55) *vs* 74% (56–87), p=0.0027) (5). The study supported widespread implementation of high quality IMRT in the

UK. Further improvements in patient reported dryness and global health scores may be achieved by optimising radiotherapy plans to reduce doses to submandibular and minor salivary glands (oral cavity), which in combination with reducing dose to the parotid glands maximises benefit in terms of patient reported dryness and HR-QoL (15). Of note, there is inter-dependency between oral dryness, dysphagia and HR-QoL outcomes. A longitudinal study of concurrent chemo-IMRT for oropharyngeal cancer showed patient-reported oral dryness was a stronger predictor of patient perceived swallowing dysfunction (HNQOL-Sw and UWQOL-Sw) than videofluoroscopy defined functional impairment (16). There is also interest in reducing dose to the brain's posterior fossa, which may account for the excess fatigue seen with IMRT (17).

The benefit of IMPT for oropharyngeal cancer

The superior dosimetric properties of protons with sharp lateral penumbra and distal fall-off reduce the radiation dose to normal tissues beyond the target volume, which compared with photons may lessen treatment-related toxicities. Radiotherapy planning studies for oropharyngeal cancer consistently show IMPT compared with IMRT reduces mean doses to organs at risk including the contralateral parotid gland and oral cavity (18-22); and reduces predicted late toxicities for oral dryness, dysphagia and aspiration (21, 23). There are observational data to support the theoretical clinical advantage of proton beam therapy for oropharyngeal cancer (24-27). In a cohort of 50 patients with locally advanced oropharyngeal cancer who received intensity modulated proton therapy (IMPT; 32/50 with concurrent chemotherapy) acute toxicities were reduced compared with a similar IMRT series from the same centre (24% *vs* 47% required a feeding tube during treatment) (28) and late toxicities were also low (late grade 2 dryness 25% at last follow-up and 12-month gastrostomy dependence 2%) (24). A subsequent prospective cohort of 150 patients treated 1:2 with IMPT:IMRT had well matched patient, tumour and treatment factors, and a median follow-up time of 32 months. The study showed a benefit for IMPT for a pre-planned composite end point of grade 3 weight loss or gastrostomy-tube dependence 12-months with an odds ratio of 0.23 (95% CI: 0.07–0.73; p=0.01) (26). A subsequent retrospective analysis from the same institute compared IMRT (n=534) and IMPT (n=50). After a median follow-up of 34 months, the lower mandibular doses (minimum 0.8 *vs* 7.3 Gy; mean 25.6 *vs* 41.2 Gy; p<0.001) for IMPT *vs* IMRT corresponded with reduced rates of osteoradionecrosis (2% *vs* 8%)(27). The first retrospective report of patient reported outcomes (PROs) using the MD Anderson Symptom Inventory (MDASI) showed modest gains from IMPT, with reduction in the 'top 5' symptoms scores up to 3 months following treatment, as well as late changes in taste and appetite (25). While the symptom burden was lower with IMPT, the study was small, observational and unbalanced. Treatments were given across different time periods with discordant systemic therapies (including now non-standard of care induction chemotherapy) and the mean follow-up times were short (7.7 *vs* 2.7 months for IMPT *vs* IMRT). Whilst dosimetric benefit of IMPT is not under question, it is internationally recognised that there is a clear need for well-designed randomised clinical trials of IMPT in oropharyngeal cancer (29).

Model-based selection for IMPT for oropharyngeal cancer

A model-based approach is being used to select head and neck cancer patients for IMPT in the Netherlands (30). The Dutch argue that randomised trials are not always the most suitable methodology for validating IMPT. Their normal tissue complication probability (NTCP) models include dose-volume parameters for organs-at-risk with models for different endpoints combined in a composite NTCP model. Their approach involves comparative planning with IMPT and IMRT to identify patients predicted to have reduced toxicity with protons. Although attractive, the approach is not prospectively validated. There are also concerns that the accuracy of model-based selection may be affected by inherent NTCP model uncertainties and differences between the planned and delivered dose (31), and the proportion of patients selected for IMPT

is also dependent on the set-up and range robustness settings used in the models (32). This method is approved by the Health Council of the Netherlands whilst recognising the need for prospective validation. Our proposed trial provides an opportunity to test model-based patient selection (akin to biomarker validation) within a randomised trial to inform future clinical trial strategies.

Description of population

Patients >18 years' old with oropharyngeal cancer, either HPV positive or HPV-negative disease, (HPV positive TNM8 T1-2 N1-2 (excluding T1 with a single ipsilateral node <3cm), T3-4 N0-2; HPV negative TNM 8: T1N2, T2N1-N2, T3-4N0-2 to be treated with concurrent chemo-radiotherapy, who meet the inclusion criteria and in the absence of any exclusion criteria, are eligible to participate in the trial.

Study rationale

Need for a randomised control trial to evaluate IMPT

The background above highlights the need for a randomised controlled trial to establish an unbiased evidence base for IMPT in oropharyngeal cancer, to provide level 1 evidence for patient benefit which justifies: inconvenience to patients and families who need to travel and stay away from home for treatment, increased treatment costs, and greater resource intensiveness (to account for treatment planning and delivery uncertainties). A randomised trial will also inform future trials in poor prognosis head and neck cancer patients aimed at dose escalation in patients with a reduced risk of toxicity with IMPT. Translational research will inform the design of future biomarker driven trials aimed at the optimal selection of IMPT *versus* IMRT based not only on NTCP models but also tumour genomics.

Standardisation, quality assurance and bias mitigation

Measures will be taken to ensure standardization and quality assurance and to mitigate against bias between the treatment groups.

1. Selection bias: Treatment allocation will be centralised and will be by minimisation with a random element. The minimisation process will account for any imbalances between IMPT and IMRT groups on important patient and tumour factors for treatment-related toxicities, as described in section 17.2.

2. Performance bias (radiotherapy): radiotherapy volume delineation and planning will be standardised using a prospectively quality-assured contouring protocol for all trial participants. The same organs-at-risk plan optimisation prioritisation parameters will be specified for all IMPT and IMRT plans to ensure the best available plans for each modality are compared in a fair way. A repeat planning CT scan is mandated in week 3 of treatment for both IMPT and IMRT patients. An objective assessment will be conducted and re-plan performed where necessary to ensure full optimisation throughout treatment.

3. Performance bias (supportive care):

All participating centres will declare at the outset whether they adopt a prophylactic or reactive approach to feeding tube insertion for all trial patients irrespective of treatment modality (see section 7). Either approach is valid; a systematic review demonstrated the impact of prophylactic versus reactive tube feeding on swallowing and swallowing-related outcomes is unclear, and there is no consensus in either the literature or UK practice (33). Long-term swallow function should not be influenced, but bias will be minimised by the randomisation centre's policy being followed for all patients it recruits to the trial (both IMRT and IMPT).

It is permissible for centres to vary from their specified policy on an individual patient basis, reasons will be collected.

Definition of feeding tube insertion for the purpose of TORPEdO trial patients is given in section 7.

Regardless of a centre's approach to feeding tube insertion, patients should be encouraged to keep swallowing (even if this is a limited amount such as sips of water) for the duration of their treatment. Each centre should report on whether or not they administer prophylactic swallowing exercises. Either approach is valid as evidence is still emerging in this area, and a Cochrane systematic review demonstrated no evidence that undertaking therapeutic exercises before, during and/or immediately after treatment leads to improvement in swallowing (34). Patients treated at the Christie or UCLH must receive the same supportive care approach independent of treatment allocation.

4. Detection/reporting bias: Follow-up after IMPT treatment will be repatriated to the referring centre with assessment schedules the same for all patients. Post treatment, patient-reported outcome measure questionnaires will be administered centrally by the ICR-CTSU and posted to patients for completion, independent of the treating clinician. Feeding tubes will be removed if not required for more than two weeks. Reasons for feeding tube removal will be collected. Feeding tube dependence will be defined as **the use of the feeding tube for nutrition within three weeks prior to the 12 month follow-up appointment**. Patients who require a feeding tube for nutrition prior to starting chemo-radiotherapy are not eligible for the study, but if randomised will be considered non-evaluable for the tube-feeding related primary endpoint. *Note that patients who have a feeding tube inserted as part of a planned prophylactic feeding tube approach (as defined above), remain eligible for the study.*

2. TRIAL OBJECTIVES

2.1 Primary objective

To assess whether IMPT compared with IMRT reduces treatment-related toxicities in patients with locally advanced oropharyngeal squamous cell carcinoma.

2.2 Secondary objectives

- To validate a biomarker (NTCP-model) as a predictor of benefit from IMPT versus IMRT (see Appendix A1).
- Define methodology and processes for future UK-led IMPT trials.
- To estimate the cost-effectiveness of IMPT versus IMRT (see Appendix A2).

2.3 Exploratory objectives

- To collect samples and treatment planning data for future linked translational research studies.
- To collect DW-MRI images to determine a threshold change in apparent diffusion co-efficient (ADC) on DW-MRI imaging of organs at risk (OAR) between baseline and week 3 of treatment that can predict patients that experience severe toxicity.

3. TRIAL DESIGN

TORPEdO is a phase III multi-centre open-label randomised controlled trial to assess whether intensity-modulated proton therapy (IMPT) compared with intensity-modulated radiotherapy (IMRT) reduces treatment-related toxicities in patients with locally advanced oropharyngeal squamous cell carcinoma.

Patients ≥ 18 years' old with oropharyngeal squamous cell carcinoma, either HPV positive or HPV-negative disease, (HPV positive TNM8 T1-2 N1-2 (excluding T1 with a single ipsilateral node <3cm)), T3-4 N0-2; HPV negative TNM 8: T1N2, T2N1-N2, T3-4N0-2, who are suitable for concurrent chemo-radiotherapy and require bilateral neck radiotherapy will be included. Patients with N3 disease, or where the local MDT decision is upfront neck dissection, or use of induction chemotherapy are excluded.

Patients who require feeding tube insertion for nutrition (for example due to dysphagia, trismus or low weight / body mass index) prior to treatment are not eligible for the trial. *It is important to note that patients who have a feeding tube inserted as part of a planned prophylactic feeding tube approach remain eligible for the study.*

Patients will be allocated in a 2:1 ratio to receive IMPT at one of the UK's NHS proton centres (The Christie or University College London Hospitals) or IMRT at their recruiting centre. Radiotherapy doses are the same in both treatment groups: therapeutic dose of 70 Gy (relative biological effective (RBE) equivalent), elective dose of 56 Gy (RBE equivalent) in 33 once daily fractions over 6.5 weeks. All trial participants should be suitable for concurrent platinum chemo-radiotherapy.

Trial participants will be followed up at the following intervals post treatment: 6 weeks, 3, 6, 12, 18 and 24 months and 3, 4 and 5 years (consistent with routine practice).

4. STUDY ENDPOINTS

4.1 Co-primary endpoints

The co-primary endpoints are:

- UW-QoL physical composite score; and
- gastrostomy dependence or CTCAE grade 3 weight loss (i.e. ≥ 20% weight loss from baseline)

measured at 12 months after completion of chemoradiotherapy.

4.2 Secondary endpoints

- Longitudinal pattern of health related quality of life (HR-QoL).
- Tube feeding status.
- Weight loss >10% from baseline at any timepoint after 6 months post-treatment.
- Acute and late severe toxicity.
- Swallowing function.
- Performance Status Scale for Head and Neck Cancer (PSS-HN).
- Hearing loss.
- Trismus.
- Resection rates.
- Loco-regional tumour control.
- Overall survival.
- Cost-effectiveness.

5. PATIENT SELECTION & ELIGIBILITY

5.1 Number of participants

The aim is to recruit 183 participants.

5.2 Source of participants

Participants will be recruited from approximately 15 participating sites in the UK. Centres are chosen to participate in the study based on successful completion of a feasibility questionnaire to determine site capacity and resources necessary to deliver the trial, previous experience of head and neck cancer trials and fulfilment of all TORPEdO RTQA requirements. In selecting sites consideration will be given to maximising access to the trial i.e. to geographic spread. Potential participants will be identified in oncology clinics and discussed at Multi-Disciplinary Team (MDT) meetings. For certain time period/s during the COVID19 pandemic recruitment may be suspended, restricted or focussed to certain (otherwise eligible) patient groups (e.g. to those geographically close to treatment centres). Details of these changes to the eligible population will be circulated to all open centres and included in the TMF.

ICR-CTSU encourages investigators to consider equality, diversity and inclusion when recruiting participants into its trials.

5.3 Inclusion criteria

- Histologically* confirmed oropharyngeal squamous cell carcinoma
- HPV positive [TNM-8] T1-2 N1-2 (excluding T1 with a single ipsilateral node <3cm), T3-4 N0-2; or
  HPV negative [TNM-8] T1 N2, T2 N1-N2, T3-4 N0-2
- Local MDT decision for concurrent chemoradiotherapy with bilateral neck treatment
- Age ≥18 years
- WHO performance status 0-1
- Adequate renal function, glomerular filtration rate (GFR) ≥60ml/min calculated using Cockcroft-Gault formula+
- Adequate cognitive ability (in the opinion of the local PI or delegated co-investigator) to complete PRO assessments
- Willingness to comply with the protocol, <u>including travel to the proton centre for IMPT treatment</u>
- Written informed consent

**Where imaging findings are in keeping with the diagnosis of oropharyngeal cancer and a biopsy of the primary site is not planned as part of routine care, histology from a neck node alone is acceptable. Cytological confirmation is also allowed in place of histology where the cytology demonstrates p16 positive squamous cell carcinoma. Where the cytology is p16 negative squamous cell carcinoma, histological confirmation and p16 status from the primary site or a neck node is required.*

+Where it is a centre's standard practice to use a different GFR estimation of AUC equivalence (e.g., Wright formula), the TORPEdO Trials Office should be notified and approach approved by the CI.

<u>For patients taking part in the optional translational biopsy sub study at The Christie the following additional inclusion criteria apply:</u>

- Patients enrolled in the TORPEdO trial with cervical lymph node involvement, i.e. p16 positive N1-2 or p16 negative N1-2c
- Involved lymph nodes must be > 2cm in diameter
- Adequate full blood count and clotting screen parameters (i.e., platelet count > 100 and prothrombin time < 14 seconds)

5.4 Exclusion criteria

- Feeding tube insertion required for nutrition (for example due to dysphagia, trismus or low weight / body mass index) prior to treatment *[Note: patients who have a feeding tube inserted as part of a planned prophylactic feeding tube approach remain eligible for the study.]*
- N3 disease
- Upfront neck dissection
- Use of induction chemotherapy
- Contra-indication to the use of cisplatin for cycle 1 concurrent chemotherapy (see section 8)
- Previous head and neck radiotherapy
- Major surgery within 6 months of trial entry
- Permanent pacemaker or implantable cardioverter defibrillator
- Any invasive malignancy within previous 2 years (other than non melanomatous skin carcinoma or cervical carcinoma in situ)
- Previous or concurrent illness (e.g., active infection, symptomatic congestive heart failure, unstable angina pectoris, cardiac arrhythmia, active peptic ulcer disease or gastritis), which in the investigator's opinion would interfere with completion of therapy, trial assessments or follow up
- Pregnancy, lactating women or women of childbearing potential unwilling or unable to use adequate non-hormonal contraception (male patients should also use contraception if sexually active)
- Pre-existing speech or swallowing problems unrelated to the diagnosis of cancer which in the local principal investigator's or delegated co-investigator's opinion would interfere with completion of therapy, trial assessments or follow up

<u>For patients taking part in the optional DW-MRI study at The Christie Hospital the following additional exclusion criteria apply:</u>

- Any contra-indication to MRI scanning, including metallic heart valve replacement, permanent pacemaker, implantable cardiac defibrillator, non-MRI compatible metal implants, neuro-stimulators.
- A history of allergy / reaction to Gadolinium contrast.

5.5 Life style guidelines

Female participants must be surgically sterile or be post-menopausal, or must agree to use effective contraception during the period of therapy and for 14 days after the last radiotherapy treatment. Effective contraception is defined as double barrier contraception (e.g. condom plus spermicide in combination with a diaphragm, cervical cap or intrauterine device).

If any trial participant becomes pregnant while receiving trial treatment or up to 30 days after treatment, this should be reported to ICR-CTSU using the pregnancy reporting form (see section 16.9).

6. SCREENING

6.1 Screening log

All participating sites will be required to keep a log of all patients with oropharyngeal cancer that are potentially eligible for this study. The information collected on the log will include:

- Date patient identified
- Screening outcome (patient approached/accepted participation/declined participation)
- Reasons for not approaching / declining participation (if available)
- Trial ID (if applicable)

This information will be used by the TMG to monitor recruitment activity. No patient identifiable data should be sent to ICR-CTSU at this stage.

6.2 Procedure for obtaining informed consent

The Principal Investigator (or designated individual) must ensure that each trial potential trial participant is fully informed about the nature and objectives of the trial and possible risks associated with participation. Potential participants should be given the current ethics approved TORPEdO patient information sheet for their consideration. Patients should only be asked to consent to the study after they have had sufficient time to consider the trial, and the opportunity to ask any further questions.

No protocol required assessments should be conducted until the TORPEdO consent form has been signed and dated by both the patient and the Investigator, unless they are performed routinely as part of standard patient care.

At the time of consent patients will also be asked to consent to donate a formalin-fixed paraffin-embedded (FFPE) diagnostic tumour tissue sample and blood samples for future translational research. Patients should be made aware that participation in the TORPEdO sample collection sub-study is entirely voluntary. Refusal to participate in the TORPEdO sub-study will not result in ineligibility to participate in the main clinical trial and will not impact the medical care received.

Confirmation of the patient’s consent and the informed consent process must be documented in the patient’s medical notes. A copy of the signed consent form should be provided to the patient and the original retained in the investigator site file, which must be available for verification by ICR-CTSU study staff at any time.

6.3 Participation in other clinical trials

Patients who fulfil the eligibility criteria will be given the opportunity to participate in TORPEdO even if they have participated in other research prior to recruitment.

Participation in other research whilst patients are being treated and followed up within TORPEdO will be considered on a study by study basis by the Trial Management Group (TMG). In addition to the patient’s best interests, the TMG’s key concern will be whether or not such participation would compromise ascertainment of the TORPEdO co-primary endpoints at the 12 month post treatment primary timepoint of interest.

The TORPEdO Trial Management Group have discussed co-enrolment into the INOVATE study (IRAS 237168) and have agreed that this should be avoided due to the numbers of blood sample required and the logistics of transfer of patients to the proton centre.

Patients enrolled from The Christie may be approached to take part in further additional audiometry sub-studies. Additional patient information and consent will be given by researchers at the University of Manchester.

7. FEEDING TUBE INSERTION

All participating centres will declare at the outset whether they adopt a prophylactic or reactive approach to feeding tube insertion for all trial patients irrespective of treatment modality.

In order to further minimise bias, feeding tube insertion and use for nutrition will be defined and standardised as follows:

(a) PROPHYLACTIC TUBE INSERTION – planned insertion of a feeding tube before radiotherapy or prior to the development of toxicities that would require tube insertion, for nutrition during treatment.
(b) REACTIVE TUBE INSERTION – insertion of a feeding tube due to the development of toxicities, for nutrition during treatment.

For both prophylactic and reactive approaches, the minimum thresholds for feeding tube use for nutrition are: >5% weight loss (CTCAE v5.0 grade 1) or other CTCAE v5.0 grade 2 toxicity (e.g. mucositis, dysphagia, nausea).

8. CONCOMITANT CHEMOTHERAPY

Chemotherapy in the context of this trial is not an investigational medicinal product.

All patients should be fit and eligible to receive chemo-radiotherapy

Cisplatin 100 mg/m^2 on day 1 and day 22 of the radiotherapy schedule will be the mandated regimen for all patients.*

Suitability for the use of concurrent cisplatin chemotherapy will be assessed and determined by local policy noting that patients are not eligible for the study if they have a calculated creatinine clearance of <60mls/min or contraindication to the use of cisplatin. Such contraindications would include peripheral neuropathy that limits instrumental activities of daily living, or hearing loss and/or tinnitus for which clinical advice has previously been sought. Audiometric assessment before randomisation is not required because we do not know if people with audiogram defined pre-existing hearing loss are more or less susceptible to oto-toxicity (35) , audiometric hearing impairment only partially correlates with functional hearing abilities (36) and the pure tone audiogram only partially explains hearing difficulty in background noise (37).

If before cycle 2 cisplatin chemotherapy participants subsequently develop renal impairment (creatinine clearance 30-60mls/min), clinically significant hearing loss or new tinnitus that interferes with activities of daily living, or neurotoxicity (peripheral neuropathy ≥ grade 2 i.e., moderate symptoms, limiting instrumental activities of daily living), or because of other toxicities (and at the discretion of the treating oncologist PI/co-investigator), carboplatin AUC=5 may be substituted for the second cycle of

chemotherapy. If the creatinine clearance is < 30 mls/min, it is anticipated that the second cycle of chemotherapy will be omitted.

*For centres unable to give concomitant cisplatin at 100 mg/m^2 due to a lack of in-patient resources, concomitant cisplatin may be given at 50 mg/ m^2 on day 1 and day 2 and then again on day 22 and day 23. Centres unable to give 100 mg/m^2 on day 1 and day 22, should contact the TORPEdO Trial Manager prior to site opening.

Centres should use local guidelines for the administration of concurrent chemotherapy. Supportive medications are as per local hospital policy.

9. PATIENT PATHWAY PRIOR TO RANDOMISATION.

- Suitable patients should be identified in the local MDT and oncology clinics.
- Standard pre-screening assessments should be completed to confirm suitability for chemo-radiotherapy.
- The trial should be discussed and TORPEDO-specific written information provided to the patient in the oncology clinic. All patients should be given the additional generic information regarding travel and accommodation for proton beam therapy.
- The patient should be given adequate time to consider participation in the trial, and typically be contacted the following day for their decision
- The patient should provide written informed consent to participate in the trial.
- Trial specific pre-screening assessments should be completed to confirm eligibility for the trial.
- Baseline PRO booklet and swallowing assessments should be completed after consent but before randomisation. In order to limit potential bias on self-reporting the PRO booklet should always be completed prior to conducting the 100ml water swallow test and the Performance Status Scale for Head and Neck Cancer (PSS-HN).

10. RANDOMISATION

Following completion of the TORPEdO baseline investigations the clinician/research nurse should contact ICR-CTSU to randomise the patient.

Patients must be randomised centrally by ICR-CTSU before trial treatment can commence. During the COVID-19 pandemic, in periods when the ICR-CTSU are working remotely, randomisations may be managed via email, randomisation-icrctsu@icr.ac.uk. Please refer to the most recent guidance provided by the trials team.

Patients should be randomised by telephoning ICR-CTSU on:
+44 (0)20 8643 7150
09.00-17.00 (UK time) Monday to Friday

The following information will be required at randomisation:

- Name of randomising hospital, consultant and person randomising patient.
- Confirmation that patient has given written informed consent for trial and for any sub-studies.
- Confirmation that patient is eligible for the trial by completion of the eligibility checklist.
- Patient’s full name, hospital number, date of birth, postcode and NHS/CHI number.
- Smoking status, site of disease, p16 status, T-stage and bilateral neck involvement.

- Confirmation that baseline PRO questionnaires, 100ml water swallow test and PSS-HN were carried out prior to randomisation.

The caller will be given the patient's unique randomisation number (Trial ID) and treatment allocation.

ICR-CTSU will send confirmation to the data management contact at the recruiting site to confirm a patients' entry into the trial.

Radiotherapy treatment should commence as soon as possible and ideally within 4 weeks after randomisation.

10.1 Patients allocated to receive proton beam therapy

For patients allocated to receive proton beam therapy, the PI or delegate must complete patient details on the NHS National Proton Therapy Referral Portal:

https://protons.protontherapyreferrals.nhs.uk/Login/Signin

Following confirmation that a patient has been appropriately referred for IMPT they will be contacted by a 'key worker', who will be a named specialist nurse or radiographer from one of the NHS proton centres. The role of the key worker is to provide assistance and support to the patient throughout the treatment. The key worker should contact the patient and referring clinician to arrange for the patient and relative/friend/carer to attend for a pre-treatment single-assessment visit, which should be within one week of receipt of the referral through the Portal. The key worker should discuss the travel and accommodation arrangements during treatment and answer any questions that the patient may have.

11. PROTON BEAM THERAPY: ACCOMMODATION AND TRAVEL

11.1 Accommodation

Patients allocated to IMPT will need to visit one of the two national NHS proton centres for treatment planning and the treatment itself. The proton centres are located at The Christie NHS Foundation Trust in Manchester and University College London Hospitals NHS Foundation Trust in London. Patients do not have to stay in the provided accommodation if they would prefer to return home every day following their treatment. The NHS will provide accommodation for the pre-assessment visit and duration of radiotherapy for one patient plus one partner or carer. The accommodation arrangements will be made by the key worker from the proton centre and only accommodation suggested by the proton centre will be funded by the NHS. Further details about the accommodation should be provided to the patient at the time of consent.

11.2 Travel

Patients will need to make their own travel arrangements. The assigned key worker should discuss the travel arrangements with the patient and answer any questions that the patient may have. Economy travel expenses for patients and/or carers will be reimbursed by the ICR-CTSU from a grant from The Taylor Family Foundation in accordance with specific terms and conditions detailed in the 'Guidance on the Reimbursement of Travel Expenses for the TORPEdO Study'. The guidance will be provided to participating centres by ICR-CTSU and should be given to all patients prior to consent.

12. TRIAL ASSESSMENTS

12.1 Screening assessments

See specific timings below for assessments 1 and 2.

1) *This assessment should be conducted within 12 weeks prior to randomisation.*

Radiological assessment of oropharyngeal cancer: all patients will be clinically assessed and radiologically staged in line with standard practice. Radiological assessment should include as a minimum: MRI* neck & CT thorax preferred, however CT neck + CT /PET CT thorax is acceptable.

2) *These assessments should be conducted within 31 days of randomisation.*

Full blood count, renal function# (Cockcroft-Gault formula+ is required before randomisation to assess for eligibility. Isotope nuclear medicine GFR is not mandated by the trial and is as per local policy.), electrolytes, liver function tests (as per local hospital policy).

3) Physical examination and WHO performance status (see Appendix A3).
4) Histology report.
5) Pre-radiotherapy dental assessment where this is possible in patients (to be conducted at randomising centre)
6) ECG/BSA (concurrent chemotherapy assessments) optional as per local hospital policy.
7) Feeding tube status (including type of tube e.g. nasogastric vs gastrostomy and tube use)
8) Patient weight (see the TORPEdO trial guidance notes regarding consistency of measurement).

** Diagnostic MRI – may be used to aid GTV definition.*

# if required by local policy, isotope nuclear medicine GFR can be conducted either pre-randomisation or pre-treatment – however GFR must be ≥60ml/min by Cockcroft-Gault+ pre-randomisation to be eligible for TORPEdO. If an isotope nuclear medicine test is performed after randomisation but before start of treatment and GFR <50 ml/min the patient cannot receive chemoradiotherapy as part of TORPEdO. In such cases, the ICR-CTSU should be notified and the patient should still be followed up to provide data for study endpoints.

+Where it is a centres standard practice to use a different GFR estimation of AUC equivalence (e.g., Wright formula), the TORPEdO Trials Office should be notified and approach approved by the CI.

<u>Patient Reported Outcomes questionnaires</u> – see Appendix A4

- University of Washington Quality of Life (UW-QoL).
- EORTC QLQ-C30 and QLQ-H&N43 questionnaires.
- M.D. Anderson Dysphagia Inventory (MDADI).
- Health economics (Healthcare resource-use, EQ-5D-5L and Work Productivity Assessment Index questionnaires (WPAI-SHP)).

Note: PRO assessment booklets will be given to the patient in clinic at baseline, at the end of treatment, at 6 weeks and then sent to the patient's home address for completion at 3, 6, 12, 18, 24, 36, 48, 60 months.

<u>Swallowing function assessments</u> - to be conducted by a speech and language therapist or where this is not possible a trained research nurse/radiographer – see section 15

- 100ml Water Swallow Test.
- The Performance Status Scale for Head and Neck Cancer (PSS-HN).

Note: In order to limit potential bias on self-reporting the swallowing assessments should always be carried out in the following order:

(1) MDADI and UW-QoL
(2) 100ml Water Swallow Test and PSS-HN.

For further details regarding the administration of the 100ml Water Swallowing Test, PSS-HN and Audiometry please refer to the Trial Guidance Notes in the TORPEdO Site Investigator File.

12.2 Pre-treatment assessments

The following assessments should be conducted after randomisation but prior to treatment.

- Audiometry (baseline audiometry should ideally be conducted at the randomising centre, for IMPT patients this should be done prior to transfer to proton centre for treatment)). Audiometry tests include Pure Tone Audiometry (PTA) in all centres. Where centres are also able to conduct audiometry in the Extended High Frequency (EHF) range and Distortion Product Otoacoustic Emissions (DPOAEs) these data will also be collected.
- Baseline signs and symptoms (NCI CTCAE v5.0).
- Maximum interincisal distance to assess for trismus. It is acceptable for this procedure to be performed pre-randomisation as part of standard of care.
- DW-MRI (for patients taking part in the optional DW-MRI sub-study at the Christie Hospital).

Radiotherapy Quality Assurance

Review of contours by the QA team will be required for all cases, to be done prospectively prior to treatment. Review of treatment plans will also be required for all cases, to be completed retrospectively by the end of week 2 of treatment. Further detail is given in the TORPEdO Radiotherapy Planning and Delivery guidelines (see section 13.2).

12.3 On-treatment assessments

The duration of radiotherapy treatment will be 6.5 weeks in both treatment groups. **During weeks 1-7 of radiotherapy treatment all patients should be assessed weekly** during IMPT and IMRT. Patients should be assessed as detailed below at the end of each week of treatment.

- Acute toxicities (including dermatitis, mucositis, dysphagia, hoarse voice, pain, weight loss, fatigue, xerostomia, alopecia) will be assessed using the NCI CTCAEv5.0 weekly during IMPT and IMRT.
- Blood tests including FBC, electrolytes, liver function tests (as per local hospital policy).
- Patient weight and WHO performance status.
- Feeding tube status (including type or tube e.g. nasogastric vs gastrostomy and tube use)
- DW-MRI (for patients taking part in the optional DW-MRI sub-study at the Christie Hospital) during week 3 of treatment.

For IMPT patients <u>all on-treatment assessments</u> will take place at the Proton centre.

12.4 End of treatment assessments (at end of week 7)

As well as the assessments listed under section 12.3 ‘On-treatment assessments’, at the visit at the end of week 7 the patient should also be asked to complete the following PRO questionnaires:

- UW-QoL, EORTC QLQ-C30, QLQ-H&N43, MDADI, EQ-5D-5L, WPAI-SHP.

12.5 Post radiotherapy treatment assessments

After treatment, clinical follow up should follow local guidelines. The study requires the following assessments:

12.5.1 At 6 weeks post treatment (timed from the end of radiotherapy)

- Clinician-reported toxicities (NCI CTCAE v5.0).
- Feeding tube status (including type or tube e.g. nasogastric vs gastrostomy and tube use).
- Patient weight + WHO performance status.
- PRO questionnaires (UW-QoL, EORTC QLQ-C30, QLQ-H&N43, MDADI, EQ-5D-5L, WPAI-SHP)).
- Clinical response assessment to determine disease response (see Appendix A5).

12.5.2 At 3, 6, 12, 18 and 24 months post-treatment (timed from the end of radiotherapy)

- **Late toxicities:** the NCI CTCAE v5.0, and LENT SOMA late radiotherapy scoring systems will be used to assess late effects occurring after radiotherapy treatment.

Late toxicity data should not be collected for patients who have relapsed and are unfit for any other procedure.

- WHO performance status.
- Audiometry and maximum interincisal distance to assess for trismus (at 3, 12 and 24 months).
- Feeding tube status (including type of tube e.g. nasogastric vs gastrostomy and tube use).
- Patient weight.

- PRO questionnaires (UW-QoL, EORTC QLQ-C30, QLQ-H&N43, MDADI, EQ-5D-5L, WPAI-SHP).
- Healthcare resource-use (3 months, 6 months, 12 months).
- Swallowing function assessments *(to be conducted after the PRO questionnaires by a speech and language therapist or where this is not possible a trained research nurse/radiographer – see section 15)*
  - 100ml Water Swallow Test
  - The Performance Status Scale for Head and Neck Cancer (PSS-HN)
- All patients will undergo clinical and radiological assessment to determine their response to treatment 12-14 weeks after completion of radiotherapy. Radiological assessment will be in line with standard practice: PET-CT is preferred, however MRI neck + CT Thorax is acceptable. Imaging may be performed earlier if clinically indicated, e.g. if there is a clinical suspicion of residual disease.
- A clinical assessment should be performed at 6, 12, 18 and 24 months to determine disease response. Further radiological review can be performed in keeping with standard practice if clinical assessment raises the suspicion of recurrent disease.

12.6 Long term follow up

At 3, 4 and 5 years post-treatment:

- PRO questionnaires (UW-QoL, EORTC QLQ-C30, QLQ-H&N43, MDADI, EQ-5D-5L).
- Assessment for recurrence and survival only.

Where possible, long term follow up will utilise information available through routinely collected data resources such as the National Cancer Registration and Analysis Service (NCRAS).

12.7 Surgical Outcomes

Surgical outcomes will be collected for patients who require salvage surgery. Indications for and date of surgery will be recorded on the eCRFs.

12.8 Discontinuation from treatment

Participants may discontinue from trial treatment at any time at their own request, or they may be discontinued at the discretion of the Principal Investigator. Reasons for discontinuation may include:

- Disease progression or recurrence
- Unacceptable toxicity
- Pregnancy

Participants who discontinue treatment should continue to be followed up.

12.9 Discontinuation from follow-up

If a patient withdraws from further follow-up a change in trial status form should be submitted to ICR-CTSU stating whether the patient simply no longer wishes to attend trial follow up visits or whether the patient has withdrawn consent for any further information to be sent to the ICR-CTSU.

12.10 Progression Events

A progression event is defined as one of the following:

- Residual or recurrent primary site disease
- Progressive or recurrent regional (neck) nodal disease
- Distant metastases.

Special consideration should be given to the following:

- residual regional nodal disease with incomplete treatment response, where a salvage neck dissection is performed that removes gross disease (R0 or R1 resection), does NOT constitute a progression event. The neck dissection typically follows concern for residual disease at the 12-14 week post-treatment reassessment scan or, where indicated for equivocal findings, an interval surveillance scan (as determined by the local PI/sub-I or MDT).
- regional nodal disease determined by the local MDT as new node(s) or progressive disease (rather than incomplete response) will constitute an event, unless a salvage neck dissection is performed that shows no viable malignant cells.
- all cases of regional only residual, progressive or recurrent disease will be reviewed centrally to confirm progression classification.

If a patient has residual, recurrent or progressive disease this should be reported to ICR-CTSU trials office in an expedited fashion as soon as this is confirmed.

12.11 Progression Events, Second Cancers and Collection of Follow-up Data

Only overall survival data will continue to be collected for the trial, where there is a progression event defined by:

- Residual or recurrent primary site disease
- Residual, progressive or recurrent regional (neck) only nodal disease NOT salvaged by a neck dissection with removal of gross disease (R0 or R1 resection)

- Distant metastases

Where there is residual, progressive or recurrent regional (neck) only nodal disease salvaged by a neck dissection with removal of gross disease (R0 or R1 resection) then standard trial follow-up including toxicity and quality of life data will continue.

Where there is a second primary cancer, standard trial follow-up including toxicity and quality of life data will continue.

Following local MDT confirmation of a progression event, collection of data for all primary and secondary endpoints, **except overall survival** should discontinue. For the avoidance of doubt, collection of overall survival data will continue at the designated time points.

Schedule of Assessments

ASSESSMENT/VISIT	Screening (Pre randomisation)	Pre-trt (After randomisation)	Treatment Period<br>For IMPT patients ALL assessments during treatment period will be at Proton Beam centre<br>RT week 1	RT week 2	RT week 3	RT week 4	RT week 5	RT week6	RT week 7	Week 6 post RT	3 months post RT	6 months post RT	12 months post RT	18 months post RT	24 months post RT	Annually at yr 3,4 & 5 post RT
Histological confirmation (p16 by IHC and TMN 8 staging)	X															
Physical examination (Including height)	X															
Patient weight +WHO PS	X		X	X	X	X	X	X	X	X	X	X	X	X	X	
Radiological assessment (MRI /PET-CT/CT thorax)	X[1]										X[2]					
ECG/BSA (concurrent chemotherapy assessments) optional as per local hospital policy	X															
Dental assessment (where possible)**either time point acceptable*	X*	X*														
Blood test (FBC, electrolytes, liver function tests)	X		X	X	X	X	X	X	X							
Calculated renal function (Cockcroft Gault Formula/see page 6)	X															
NM isotope GFR (optional, per local policy) **either time point is acceptable*	X*	X*														
5-point customised thermoplastic shell and radiotherapy planning CT scan		X														
Repeat radiotherapy planning CT scan					X[4]											
Radiotherapy planning re-plan						X[5]										
Adverse events & toxicities: CTCAE (+LENT SOMA 3-24mths)		X	X	X	X	X	X	X	X	X	X[6]	X[6]	X[6]	X[6]	X[6]	
Clinical response assessment [7]										X	X	X	X	X	X	X
Audiometry		X[3]									X		X		X	
Maximum interincisal distance to assess for trismus **either time point is acceptable*	X*	X*									X		X		X	
Feeding tube status	X		X	X	X	X	X	X	X	X	X	X	X	X	X	
PRO questionnaires[8] (UW-QOL, EORTC QLQ-C30, QLQ H&N 43, MDADI, EQ-5D-5L, Healthcare resource use[9] and WPAI-SHP[10])	X								X	X	X	X	X	X	X	X
Swallowing function assessments (100ml water swallow test + PSS-HN)	X										X	X	X	X	X	
**Translational sub-study (optional)**																
EDTA blood (10 ml) for genomics[12]		X[11]														
Streck bloods(40ml pre-trt; 30ml thereafter)for liquid biopsies[12]		X[11]				X				X	X					
EDTA bloods (30 ml) for immune markers[12]		X[11]			X			X		X						
EDTA bloods (10 ml) for proteomics		X[11,13]	X	X	X	X	X	X	X		X					
Diagnostic FFPE block request for transcriptomics		X														
**DW-MRI sub-study (optional)[14]**		X			X											
**Biopsy sub-study (optional)[14]**		X			X											

[1]Baseline: MRI neck + CT thorax preferred, however CT neck + CT thorax is acceptable. [2]At 3 months, PET-CT is preferred, however MRI neck + CT Thorax acceptable (may be performed earlier if clinically indicated). [3]baseline audiometry should ideally be conducted at randomising centre. [4]Repeat planning CT scan to be ideally performed on Wednesday of week three. [5]Re-plan where needed, to start on new plan Monday of week five. [6]LENT SOMA as well as CTCAE assessments. [7]Additional tests will be requested if clinically indicated as in standard practice. [8]PRO booklets will be given to

the patient in clinic at screening, end of treatment, 6 weeks post RT, and then sent to the patient's home address for completion at 3, 6, 12, 18, 24, 36, 48, 60 months. [9]Healthcare resource use questionnaire only completed at screening, and 3, 6, and 12 months post RT. [10] WPAI-SHP questionnaires not completed years 3-5. [11]Samples will be taken as described in the Sample Collection Manual [12] Samples to be collected and sent the same day on Mon-Weds only, and not two days prior to a bank holiday. [13]Two proteomic samples taken pre-treatment. [14]Patients recruited by The Christie only.

13. TREATMENT

Patients entered in the TORPEdO trial are randomised to receive either intensity modulated proton therapy (IMPT) at The Christie (Manchester) or UCLH (London), or intensity modulated radiotherapy (IMRT) at their local radiotherapy centre.

Radiotherapy treatment should commence as soon as possible and ideally within 4 weeks after randomisation.

For IMPT and IMRT, treatment is via a simultaneous integrated boost in a single phase, as follows:

- 70Gy(RBE) in 33 fractions (2.12 Gy(RBE) per fraction) over 6.5 weeks to the therapeutic clinical target volume (CTV1)for IMPT and the therapeutic planning target volume (PTV1) for IMRT.

- 56Gy(RBE) in 33 fractions (1.70Gy(RBE) per fraction) over 6.5 weeks to areas at risk of microscopic disease (CTV2 for IMPT and PTV2 for IMRT))

13.1 Patient immobilisation and image acquisition

All patients should be immobilised in a supine position using a 5 point customised thermoplastic shell. Patients should undergo a radiotherapy planning CT scan wearing the shell, with a maximum CT slice thickness of 3mm, from the vertex of the skull superiorly to the carina inferiorly.

For both IMPT and IMRT planning scans, the use of intravenous contrast is recommended in order to facilitate optimal delineation. For IMPT, an additional pre-contrast scan should be acquired and rigidly co-registered with the post-contrast scan.

An MRI scan of the neck, either diagnostic or in the treatment position, is also recommended to aid delineation of the target volumes and organs at risk. Rigid or deformable registration of the MRI scan with the planning CT may be helpful, depending on a clinical assessment of the quality of co-registration.

13.2 Target outlining and planning

Radiotherapy planning and outlining should be carried out in accordance with the guidelines in the current version of the TORPEdO Radiotherapy Planning and Delivery guidelines ("Quality Assurance (QA) pack") available on request from ICR-CTSU TORPEdO-icrctsu@icr.ac.uk) or found on the Radiotherapy Trials Quality Assurance (RTTQA) website www.rttrialsqa.org.uk.

13.3 Verification and replanning

Volume delineation, planning and treatment will take place locally (IMRT) or at The Christie or UCLH (IMPT).

Patient specific dose verifications will be undertaken before the patient begins treatment as per local practice.

Local practice should be followed. As a minimum it is recommended that cone beam CT (CBCT) images should be acquired for the initial three fractions and assessed off-line, with weekly images taken thereafter. If a weekly image has an out of tolerance error, verification images should be repeated for the next 2

treatments to allow for calculation of a new systematic shift. Any systematic correction applied should be confirmed using imaging after being first applied.

Patients will have a repeat planning CT scan during week 3 (IMPT and IMRT), ideally on the Wednesday. This scan will be co-registered with the initial planning CT scan and volume delineation contours transferred from initial planning CT scan to the verification CT scan for review by the local Clinical Oncologist.

Following review and where necessary, only OAR and elective nodal contours will be amended. The therapeutic target volume contours (i.e. the geometric expansions of 5+5m or 10mm around the primary or nodal disease) are not amended/adapted to a change in GTV. Doses to targets, electively treated volumes and OARs will be re-calculated based on the original plan and coverage of target volumes reassessed.

A replan is required if:

- Target coverage requires improvement.
- Cumulative maximum dose to critical structures is >2% over tolerance
- Mean dose to parotids/oral cavity/submandibular glands have increased by >3 Gy(RBE) assuming the entire treatment will be delivered as per the verification scan.

A threshold of 3 Gy(RBE) was selected as corresponding to a difference of up to 10% in NTCP for the mean dose model in the linear range (25 Gy(RBE) to 55 Gy(RBE)) (38). Patient-reported dryness is improved by oral cavity and submandibular gland sparing, and by extrapolation a re-planning threshold of delta 3 Gy(RBE) will be applied.

If replanning is required then the patient should begin on the new plan as soon as possible, ideally on or before the Monday of week 5. Changes in patient anatomy and setup will be monitored during treatment with re-plans permitted where required. The reasons for replanning will be collected and plans retrospectively reviewed for quality assurance.

13.4 Treatment Interruptions

Patients are treated as Category 1, defined by The Royal College of Radiologists (39). Gaps in treatment should (if clinically appropriate) be compensated to ensure treatment is delivered within the same overall treatment time (6.5 weeks). This will be by treatment with 2 fractions per day with an inter-fraction interval of at least 6 hours or patients may be treated on a weekend day (Saturday or Sunday). In this situation, patients should receive a maximum of six fractions per week.

Patients should not have a break in treatment where possible. Where interruptions are due to breakdown or unavailability of the treatment machine, patients should be transferred to a matched treatment machine in the first instance. Other causes of treatment interruption may be mitigated by treatment on weekends and bank holidays.

For proton patients, in the event of cyclotron downtime that cannot be mitigated by weekend treatment, patients will be prioritised to receive a photon backup plan according to standard departmental policy.

An IMRT contingency plan will be prospectively generated for each patient receiving IMPT so that in case of breakdown, patients will receive IMRT until service restored ensuring no treatment gaps. If used, these plans will be retrospectively reviewed for quality assurance.

14. RADIOTHERAPY QUALITY ASSURANCE

The radiotherapy quality assurance (RT QA) programme for the TORPEdO trial has been designed and will be implemented by the National Radiotherapy Trials QA (RTTQA) Group. QA requirements will be reduced for centres participating in trials involving IMRT treatment for head and neck cancer, where appropriate. The components of the TORPEdO QA programme are described in the TORPEdO Radiotherapy Planning and Delivery guidelines ("Quality Assurance (QA) pack").

The full details of the programme are available on the RTTQA group website (www.rttrialsqa.org.uk).

15. ASSESSMENT OF SWALLOWING FUNCTION

Swallowing function should be evaluated at baseline and **3, 6, 12, 18 and 24 months** following the completion of treatment using a Water Swallow Test and the Performance Status Scale for Head and Neck cancer (PSS-HN).

15.1 100mL water swallow test (WST)

The Water Swallow Test (WST) should be conducted by Speech & Language Therapists (SLTs). Where assessment by a SLT is not feasible within the time/resource constraints of the SLT clinic, assessments may be conducted by a research nurse or radiographer who has been trained by a SLT with reliability checks on assessment of ten volunteers. Prior to administering the WST nurses will be requested to submit confirmation of proven reliability for a minimum of 10 examinations to the ICR-CTSU.

Sites should decide how to organise the assessments for their patients and should confirm that staff conducting the assessment are appropriately trained. The staff conducting the WST at each centre should, where possible, remain consistent throughout the course of the study.

The test requires a stopwatch, syringe or measuring cup, plastic cup and tap water and should be carried out as detailed in the TORPEdO Trial Guidance Notes in the Site Investigator File. There are instructions for both in person and remote assessments.

The assessor records:
1. the number of swallows taken;
2. the time taken to complete task (seconds);
3. the presence/absence of throat clear or cough.

The WST should not be attempted on individuals who have been recommended by their managing clinician to remain nil by mouth. However, the test may form part of a clinical swallowing examination to determine the patient's readiness for return to oral feeding, if deemed appropriate by the managing clinician.

If there are overt signs of significant aspiration (explosive coughing, prolonged coughing) or the patient is becoming distressed, the assessment should be stopped and the remaining amount in the cup measured and recorded. Those patients should be highlighted to SLTs for a more detailed review.

- Measures of swallow capacity (mL/ time) and swallow volume (mL/ number of swallows) are derived from the data in the Functional Outcome Panel SOP as detailed above.

15.2 Performance Status Scale for Head and Neck Cancer (PSS-HN).

The PSS-HN is a 3-item scale designed to evaluate functional performance of head and neck cancer patients, specifically:

- Normalcy of Diet;
- Eating in Public;
- Understandability of Speech.

Each subscale is rated based on an unstructured interview with a score of 0 to 100, with higher scores indicating better performance. The subscale scores are reported separately, and the Normalcy of Diet subscale will be the primary score of interest for this trial.

The PSS-HN takes approximately 5 minutes to complete and is inexpensive to administer. The PSS-HN has been incorporated into the UK Dataset for Head and Neck Oncology (DAHNO) and is a National Comprehensive Cancer Network (NCCN) recommended measure in the US. It also allows for the assessor to note enteral tube feeding status alongside these scores.

The PSS-HN should be rated by SLTs. The person collecting these data should remain consistent where possible throughout the course of the study. Scores are determined following an unstructured interview. Where assessment by a SLT is not feasible within the time/resource constraints of the SLT clinic, assessments may be conducted by a trained research nurse or radiographer who should be trained by the SLT. Prior to administering the PSS-HN, research nurses and radiographers are required to submit the PSS training confirmation form to ICR-CTSU.

16. SAFETY REPORTING

16.1 Definitions

Adverse Event (AE)
An AE is any untoward medical occurrence in a patient administered a study treatment; the event does not necessarily have a causal relationship with the treatment.

Serious Adverse Event (SAE)
An SAE is any untoward medical occurrence that occurs after the commencement of radiotherapy treatment and within 30 days of the last radiotherapy treatment and:

- results in death,
- is life-threatening
- requires hospitalisation or prolongation of existing inpatients´ hospitalisation
- results in persistent or significant disability or incapacity
- is a congenital anomaly or birth defect

For those patients taking part in the optional biopsy sub-study (The Christie only): an SAE should be reported if any of the above SAEs were caused by the patient having a biopsy.

Important adverse events that are not immediately life-threatening or do not result in death or hospitalisation, but may jeopardise the subject or may require intervention to prevent one of the other outcomes listed in the definition above, may also be considered serious.

Progression of the indicated disease and death due to progression of the indicated disease are not considered SAEs.

Pregnancy or aid in the conception of a child whilst participating in a trial is not itself considered an SAE but should be followed up for congenital anomalies or birth defects.

Note

Hospital admissions for the following events do not require immediate reporting on the TORPEdO SAE form but any AEs should be recorded in the TORPEdO electronic Case Report Forms:

- admission for administration of chemotherapy
- admission for symptom control for expected acute toxicities (see section 16.6) for chemotherapy and radiotherapy (CTC grade ≤3 only)
- admissions secondary to tumour recurrence
- admission for palliative treatments, terminal care or elective surgery
- Outpatient treatment in accident and emergency/casualty is not itself an SAE, although the reasons for it may be.
- Hospital admissions/surgical procedures planned for a pre-existing condition before a patient is randomised to the study are not considered SAEs, unless the illness/disease deteriorates in an unexpected way during the study.

Serious Adverse Reaction (SAR)
A serious adverse reaction is an SAE that is suspected as having a causal relationship to the trial treatment, as assessed by the investigator responsible for the care of the patient. A suspected causal relationship is defined as possibly, probably or definitely related (see definitions of causality table).

Table 1: Definitions of causality

Relationship	Description
Unrelated	There is no evidence of any causal relationship with the trial treatment
Unlikely	There is little evidence to suggest there is a causal relationship (e.g. the event did not occur within a reasonable time after administration of the trial treatment). There is another reasonable explanation for the event (e.g. the patient's clinical condition, other concomitant treatment)
Possible	There is some evidence to suggest a causal relationship (e.g. because the event occurs within a reasonable time after administration of the trial treatment). However, the influence of other factors may have contributed to the event (e.g. the patient's clinical condition, other concomitant treatments)
Probable	There is evidence to suggest a causal relationship, and the influence of other factors is unlikely
Definitely	There is clear evidence to suggest a causal relationship, and other possible contributing factors can be ruled out
Not	There is insufficient or incomplete evidence to make a clinical judgement of the

assessable	causal relationship.

Related Unexpected Serious Adverse Event
An adverse event that meets the definition of serious and is assessed by the CI or nominated representative as:

- "Related" – that is, it resulting from administration of any of the research procedures, and
- "Unexpected" – that is, the type of event is not listed in the protocol as an expected occurrence (see Section 16.6)

16.2 Reporting Adverse Events to ICR-CTSU

Any toxicity, sign or symptom that occurs after commencement of radiotherapy treatment and within 2 years of the last administration of radiotherapy treatment, which is not unequivocally due to progression of disease, should be considered an AE.

All AEs must be reported on the relevant eCRF and submitted to ICR-CTSU.

The severity of AEs should be graded according to the NCIC-CTC/CTCAE v5.0 criteria (see TORPEdO Site Investigator File). For each AE, the highest grade observed since the last visit should be reported.

Whenever one or more toxicity/sign/symptom corresponds to a disease or a well-defined syndrome only the main disease/syndrome should be reported.

16.3 Reporting of Serious Adverse Events to ICR-CTSU

Any SAE that occurs after the commencement of radiotherapy treatment and up to 30 days following the last administration of radiotherapy treatment must be reported.

All SAEs, apart from "hospital admissions not requiring immediate reporting (previous page)" should be reported to ICR-CTSU within 24 hours of the Principal Investigator (or designated representative) becoming aware of the event, by completing the TORPEdO SAE form and emailing to:

The ICR-CTSU safety desk
sae-icr@icr.ac.uk For the attention of the TORPEdO Trial team

As much information as possible, including the Principal Investigator's assessment of causality, must be reported to ICR-CTSU in the first instance. Additional follow up information should be reported as soon as it is available.

All SAE forms must be completed, signed and dated by the Principal Investigator or designated representative.

16.4 Review of Serious Adverse Events

The Chief Investigator (or designated representative) will assess all reported SAEs for causality and expectedness (NB. The Chief Investigator cannot down-grade the Principal Investigator's assessment of causality.)

SAEs assessed as having a causal relationship to radiotherapy and as being unexpected will undergo expedited reporting to the relevant authorities and all other interested parties by ICR-CTSU (see 14.4).

Sites should respond as soon as possible to requests from the Chief Investigator or designated representative (via ICR-CTSU) for further information that may be required for final assessment of an SAE.

16.5 Expedited Reporting of Related Unexpected SAEs

If an SAE is identified as being related and unexpected by the Chief Investigator it will be reported by ICR-CTSU to the main REC, the Sponsor and all other interested parties within 15 days of being notified of the event.

The Principal Investigators at all actively recruiting sites will be informed of any related unexpected SAEs occurring within the trial at appropriate intervals.

16.6 Anticipated treatment related SAEs

It is expected that patients receiving chemo-radiotherapy for head and neck cancers commonly require admission for symptom control (for example: mucositis, dehydration, dysphagia, pain, anaemia, nausea and vomiting, constipation, weight loss, poor oral intake, tinnitus (chemotherapy related), infection which includes chest infection). They will require intravenous rehydration, nasogastric feeding tube (NG) or percutaneous endoscopic gastrostomy (PEG) feeding and pain control. If unsure as to whether an event is considered as anticipated within the trial please contact ICR-CTSU.

For patients taking part in the additional biopsy sub-study (The Christie only) expected acute toxicities are; discomfort secondary to administration of local anaesthetic, bruising, haematoma (rare). No late toxicities related to neck node biopsy are expected. In the event of a serious adverse event considered to be directly related to the biopsy procedure, the delegated investigator at Christie will be contacted to assess the causality and expectedness of the event.

Note: For the purposes of this protocol anticipated treatment related SAEs of grade ≤3 are not subject to expedited reporting should still be recorded in the eCRFs.

16.7 Follow up of Serious Adverse Events

SAEs should be followed up until clinical recovery is complete or until disease has stabilised. SAE outcomes should be reported to ICR-CTSU using the relevant section of the SAE form as soon as the Principal Investigator or designee becomes aware of the outcome.

16.8 Annual Safety Reporting

An annual progress report will be provided to the main REC by ICR-CTSU and copied to the Sponsor at the end of the reporting year. This will include data about related unexpected SAEs and whether any safety concerns have arisen during the reporting period.

16.9 Reporting Pregnancies

If any trial participant becomes pregnant while receiving trial treatment or up to 90 days after receiving trial treatment, this should be reported to ICR-CTSU using the pregnancy reporting form. Participants who become pregnant should discontinue from trial treatment immediately. Pregnancies should be followed up until conclusion and all follow-up information should be reported to ICR-CTSU. If the outcome of the pregnancy meets the definition of serious (i.e. congenital abnormality) this should be reported to ICR-CTSU following the serious adverse event reporting procedures described above.

Figure 1: : Flow diagram for SAE reporting, and action following report

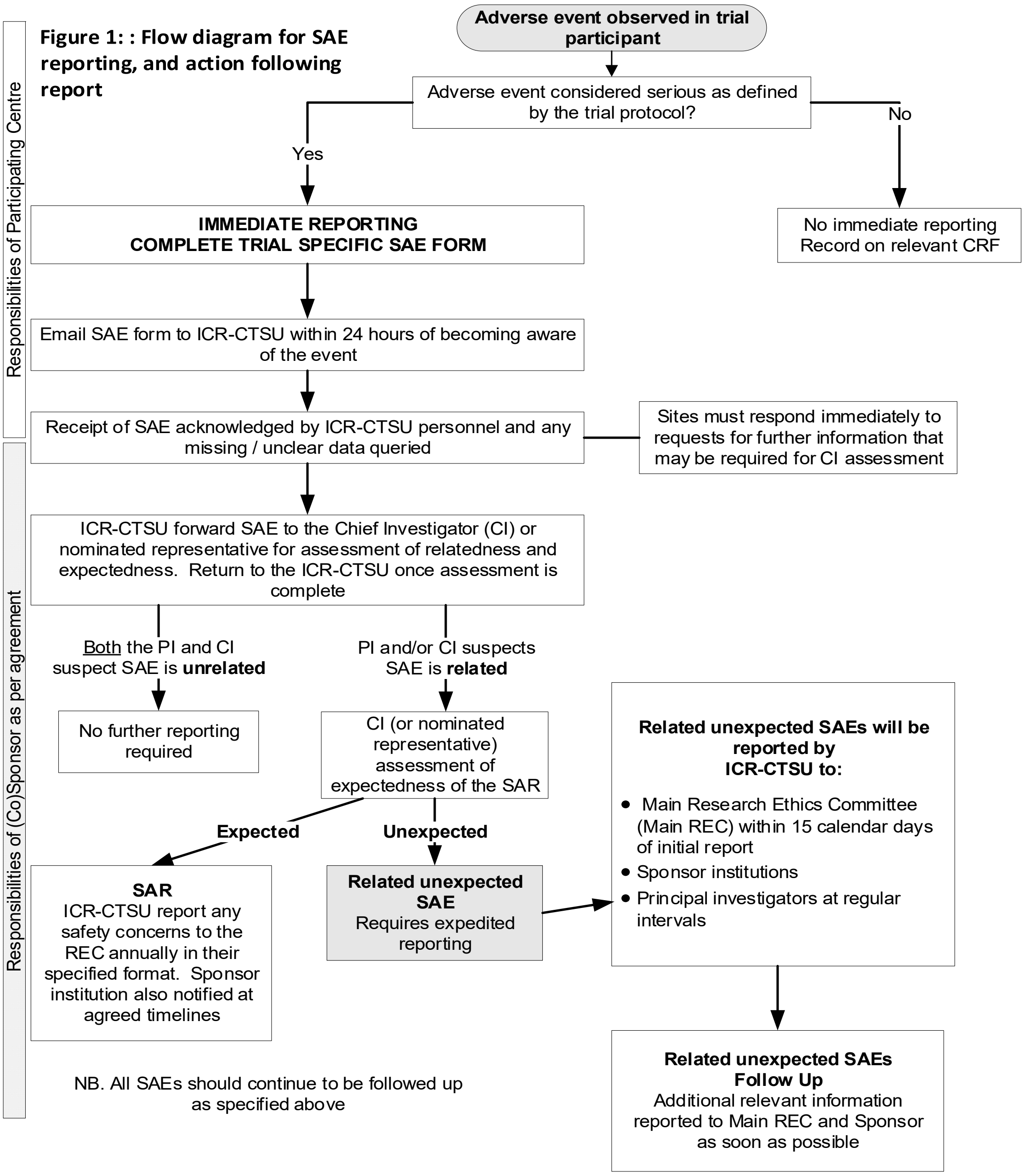

17. STATISTICAL CONSIDERATIONS

17.1 Statistical Design and Sample Size Justification

17.1.1 Statistical Design

TORPEdO is a phase III multi-centre open-label parallel-group randomised controlled trial. Co-primary endpoints have been selected:

- UW-QoL physical composite score
  A patient-reported outcome measure was chosen as a highly relevant way to test the ability of proton therapy to reduce late toxicity and improve quality of life for patients. This was supported in patient focus groups, by clinicians and commissioners as a valid comparator, which if satisfied would lead to a change in practice. The potential for bias by clinicians is minimised as the questionnaire will be administered centrally by the ICR-CTSU and posted to patients for self-completion, independent of treating clinicians or the proton centres.

- Gastrostomy dependence or grade 3 weight loss (≥ 20% from baseline i.e. CTCAE grade 3 toxicity)
  This is a highly relevant endpoint as long-term gastrostomy dependence has profound negative impact on quality of life for patients (7), and is a surrogate for severe swallowing dysfunction and other functional impairments such as problems with chewing, taste disturbance or oral dryness. The potential for physician-bias in tube removal is mitigated by the composite inclusion of grade 3 weight loss, where premature tube removal would likely translate into increased weight loss.

The intention is show superiority of IMPT over IMRT in terms of the patient reported and clinician reported co-primary outcome measures but demonstration of a significant treatment effect on either of the co-primary endpoints will be considered sufficient to support a conclusion of effectiveness. As such the Bonferroni method has been used to protect the family wise error rate associated with using two primary endpoints. The Bonferroni method tends to be conservative for the study overall Type I error rate if the endpoints are positively correlated but this approach has been taken to provide additional power for secondary endpoints, to mitigate against minor departures from the control arm event rates and to maximise conjunctive power (albeit with larger effect patient reported outcome effect size) should this need to be considered in the future.

The sample size requirements are driven by the clinician reported composite endpoint thus this is considered first.

17.1.2 Sample size/power considerations for clinician reported co-primary endpoint

The clinician reported co-primary endpoint is a composite binary endpoint where an “event” is
- gastrostomy dependence or;
- CTCAE grade 3 weight loss (≥ 20% from baseline)

at 12 months after completion of chemoradiotherapy.

This endpoint was used in a prospective cohort study (26) including 150 patients treated 1:2 with IMPT or IMRT, matched by patient, tumour and treatment factors. In this study, gastrostomy-tube dependence or grade 3 weight loss after treatment favoured IMPT with rates of 25% for IMRT and 8% for IMPT, an odds ratio of 0.23 (95% CI: 0.07–0.73; p-value = 0.01). The event rate in the IMRT group is consistent with rates

of feeding tube dependence alone at 12 months after treatment reported in a systematic review (average 18%) (10) and with data published from The Christie (16%) (11).

The event rate in the IMRT control group is thus assumed to be 25%. The target treatment effect was selected based on an odds ratio of 0.23 translating to a 7% event rate in the IMPT group. Using a chi-squared based comparison of proportions, with 2:1 randomisation (IMPT:IMRT), 80% power, and 2-sided 2.5% significance, 165 participants (110 IMPT: 55 IMRT) are required to detect a difference of 25% (IMRT) versus 7% (IMPT) in the proportion of patients with feeding tube dependence or grade 3 weight loss, at 12 months.

An inflation of 10% has been applied to allow for non-evaluability (e.g. due to drop-out due to disease recurrence or death before 12 months, non-compliance with the 12 month questionnaire or requirement for tube feeding prior to commencement of chemo-radiotherapy). This gives a target sample size of 183 participants (122 IMPT: 61 IMRT).

17.1.3 Sample size/power considerations for patient reported co-primary endpoint

The patient reported co-primary endpoint is the UoW physical composite score. In a previous cohort of head and neck cancer patients treated with IMRT, mean UW-QoL physical composite score 12 months after treatment completion was 71.2 (SD=14.05), compared to 89.3 at baseline (40). An 8-point improvement represents a 'moderate' and clinically important increase and is consistent with: 1) an NTCP model based estimate of the mean difference of 13 points at 12 months (30, 41) and 2) the change in EORTC QLQ-C30 Physical Function of 9 points at 12 months seen between patients treated with VMAT and Protons (42, 43). With 156 participants (104 IMPT: 52 IMRT) an 8-point improvement in the mean UW-QoL physical composite scale (assuming equal SD of 14.05 in each group) can be detected at the two-sided 2.5% significance level with 86% power (two-sample t-test).

In a contemporary ICR-CTSU managed head and neck cancer radiotherapy trial (DARS) where HR-QoL questionnaires are centrally administered, return rates at 12 months post-treatment are approximately 85%. Applying a 15% inflation for non-evaluability gives the target sample size of 183 participants (122 IMPT: 61 IMRT).

17.1.4 Sample size/power considerations for Secondary Research Question

An NTCP biomarker model (with a 3 outcome composite endpoint, SUM-NTCP: NTCP xerostomia ≥grade 2, NTCP dysphagia ≥grade 2, NTCP tube feeding dependence) may identify a subgroup of patients who would benefit most from IMPT vs IMRT. The six physical symptoms comprising the UW-QoL physical subscale were related to the EORTC QLQ-HN35 questionnaire to comprise a UWQoL- equivalent scale. The Dutch models select patients for IMPT based on the predicted NTCP profile 6 month after completion of treatment. Data were available to show that a predicted difference in SUM-NTCP (IMRT vs IMPT) of 15% at 6 months corresponded with a mean difference in UW-QoL score of approximately 18 points at 12 months. The IMRT and IMPT plans for each TORPEdO participant will be used to validate the NTCP model i.e. predicted vs observed toxicity. We also hypothesise that for an enriched subgroup of patients (approximately 50%) with a predicted difference in SUM-NTCP (IMRT vs IMPT) of 15% at 6 months, there would be a moderate to large observed increase in UW-QoL physical composite scale at 12 months of ≥15 points (which is conservative based on an estimated predicted difference of 18 points). With approximately 150 evaluable patients overall (as estimated for the patient reported co-primary endpoint) and assuming 50% in each of the enriched and unenriched subgroups, the precision of the estimate of difference between the two

subgroups in mean change of HR-QoL scores would be approximately +/- 4.5 points (2-sided 95% confidence interval); a standard deviation of 14.05 is assumed for the mean UW-QoL, as specified for the patient-reported co-primary endpoint in section 17.1.3.

17.1.5 Review of sample size assumptions

An initial review of the statistical assumptions underlying the power calculations for each of the co-primary endpoints will be undertaken after 25 control arm patients have been followed up for 12 months. If the clinician reported outcome event rate is much lower or the mean UoW physical composite score is much higher (better) in the control arm than assumed for the power calculations this may impact on the likelihood of detecting a significant improvement with IMPT. In this scenario, or if the non-evaluability rate or rate of withdrawals prior to study treatment due to significant co-morbidity (from both arms) is much higher than anticipated, the IDMC and/or TSC (as appropriate) will be asked to advise on the statistical power to detect meaningful differences in the IMPT arm and whether any changes are needed to the sample size.

17.2 Treatment Allocation

Patients will be centrally randomised via ICR-CTSU to treatment groups in a 2:1 allocation ratio (IMPT: IMRT). The use of 2:1 randomisation aligns with NHS England's preference to increase access to proton therapy for patients within clinical trials and increases power for secondary and exploratory endpoints relating to IMPT.

Treatment allocation will be by minimisation with a random element to account for imbalances between IMPT and IMRT groups ensuring comparability of clinically important pre-specified prognostic factors (balancing factors) between treatment groups. Details of how factors are categorised for the purposes of balancing are provided in the Statistical Analysis Plan.

Balancing factors are:

- randomising centre
- bilateral neck nodes
- site of disease
- p16status
- smoking status
- T-stage

17.3 Endpoint Definitions

17.3.1 Primary endpoints

The co-primary endpoints are:

- UW-QoL physical composite score; and
- gastrostomy dependence or CTCAE grade 3 weight loss (i.e. ≥ 20% weight loss from baseline)

measured at 12 months after completion of chemoradiotherapy.

The UW-QOL is a validated patient reported quality of life tool examining 12 quality of life domains pain, appearance, activity, recreation, swallowing, chewing, speech, shoulder function, taste, saliva, mood and anxiety (13, 44, 45). A score is provided from each domain ranging from 0 to 100, with higher scores

indicating better function. The composite physical score is generated by computing the average of six scores – swallowing, chewing, speech, taste, saliva and appearance with the requirement that at least 4 domain scores are available.

Feeding tube dependence will be defined as use of the feeding tube for nutrition within 3 weeks prior to the 12 month follow-up appointment. In the rare event that a randomised patient requires tube feeding prior to commencement of radiotherapy (as opposed to a prophylactic or reactive feeding tube approach as defined in section 7) they will be considered non-evaluable for the primary endpoint.

17.3.2 Secondary endpoints

17.3.2.1 Longitudinal pattern of HR-QoL

HR-QoL will be evaluated using the PRO questionnaires:

- University of Washington Quality of Life (UW-QoL) (13, 44, 45)
- EORTC QLQ-C30 and QLQ-H&N43 (46-49)
- M.D. Anderson Dysphagia Inventory (MDADI) (50)

All questionnaires are validated. Continued assessment past the primary endpoint for up to 5 years following treatment is proposed to allow further investigation into the long-term pattern of improvement from radiation side-effects. Analyses will focus (but will not be limited to) QoL subscales that relate to physical functioning and will include: physical function and social-emotional function composite UW-QoL scores, global health, appetite loss, physical and other functioning subscale scores from the EORTC QLQ-C30, the problems with swallowing, dry mouth/sticky saliva, social eating and problems with teeth subscales of the QLQ-H&N43 module, and the MDADI composite and global, emotional, functional and physical subscale scores. Scores will be computed in line with relevant scoring manuals.

17.3.2.2 Tube feeding status

Evaluated at 3, 12, 18 and 24 months after completion of treatment. Time to feeding tube requirement (defined as the time from randomisation to the patient requiring a feeding tube for nutrition) will also be analysed.

17.3.2.3 Acute and late severe toxicity

Physician recorded CTCAE v 5.0 severe toxicity is defined as grade 3 or worse. Acute toxicity is defined as treatment emergent adverse events occurring up to 3 months post treatment; late toxicity is evaluated up to 5 years.

17.3.2.4 Swallowing function

Evaluated using the 100-mL Water Swallowing Test (51). Two separate scores will be generated and analysed for each patient, swallow volume and swallow capacity.

17.3.2.5 Performance Status Scale for Head and Neck Cancer (PSS-HN)

The Normalcy of Diet and Place of Eating scores will be reported separately (52, 53).

17.3.2.6 Hearing loss

Hearing will be measured by audiometry using Pure Tone Audiometry (PTA), including audiometry in the Extended High Frequency (EHF) range, and Distortion Product Otoacoustic Emissions (DPOAEs) where possible. The definition of hearing loss will be agreed with expert audiology input – Prof Kevin Munro, Professor of Audiology, Manchester and specified in the Statistical Analysis Plan prior to any comparative analysis being conducted.

17.3.2.7 Trismus

Evaluated using change in maximum interincisal distance.

17.3.2.8 Resection rate

Defined as the proportion of patients proceeding to surgical treatment (including neck dissection) after the completion of radiotherapy treatment.

17.3.2.9 Loco-regional tumour control

Defined as time from randomisation to loco-regional recurrence i.e. recurrence at the primary site or in the neck. Patients will be censored at time of distant recurrence or death prior to loco-regional recurrence. Location (including location relative to treatment field) of loco-regional tumour recurrence will also be of interest.

17.3.2.10 Overall survival

Defined as time from randomisation to death from any cause.

17.3.2.11 Cost-effectiveness

Analysis will utilise the Healthcare resource use questionnaire developed for the trial, Work Productivity Assessment Index (WPAI-SHP) (54, 55) and EuroQol five-dimensional questionnaire (EQ-5D-5L) (56). Analysis will be expressed as Cost per Quality Adjusted Survival (Appendix A2)

17.3.2.12 Weight loss >10% from baseline at any timepoint after 6 months post-treatment.

In addition to CTCAE grade 3 weight loss (≥ 20% weight loss from baseline), weight loss from baseline >10% at 12, 18 and 24 months will be reported.

17.4 Statistical Analysis Plan

Further details of analysis methods will be specified in a Statistical Analysis Plan in accordance with ICR-CTSU Standard Operating Procedures. The Statistical Analysis Plan will include a section to detail how data impacted by strategies introduced to mitigate risks relating to the COVID19 pandemic will be handled.

17.4.1 Co-primary endpoints

UW-QoL physical composite score will be compared between randomised groups using a two-sample t-test or non-parametric Mann-Whitney test if the scores do not follow a normal distribution. The proportion of patients gastrostomy dependent or with CTCAE grade 3 weight loss (i.e. ≥ 20% weight loss from baseline will be compared between randomised groups using a chi-squared or Fishers exact test as appropriate.

The primary analysis will be according to intention to treat principle and will include all randomised patients. Analysis in a per-protocol population will also be performed if significant deviations from protocol are identified. Although the primary analysis of the co-primary endpoints will be non-stratified,

consideration will also be given in the statistical analysis plan to multivariable sensitivity analyses including adjustment for balancing and other prognostic factors.

If a significant proportion of patients are not evaluable, the pattern of missing data will be assessed and if the data appear not to be missing at random, i.e. if there are potential differences in UW QoL physical composite outcome, between evaluable and non-evaluable patients, a sensitivity analysis assuming different patterns of missing data may be performed. The treatment effect, between the enriched and unenriched subgroups, as predicted by the NTCP biomarker model (Appendix A1), will be compared by estimating the mean difference of the change in UW QoL physical composite score at 12 months, with 95% confidence intervals.

Due to the large number of secondary endpoints, analysis will focus on description with significance testing limited to key endpoints and timepoints to be defined prospectively in the Statistical Analysis Plan.

HR-QoL subscale scores will be presented graphically at each timepoint and compared between arms at specific time points using ANCOVA. Multiple regression models (e.g. ANCOVA) will be used to investigate other patient and clinical factors that may be associated with change in UW-QoL physical composite score The primary timepoint of interest will be 12 months post-treatment to correspond with assessment of the co-primary endpoints (with a significance level of 1% applied to statistical tests). Longitudinal data modelling methods e.g. generalised estimating equations will be used to evaluate change in HR-QoL over time. In the presence of missing data on HR-QoL questionnaires, subscale scores will be calculated according to advice given in the relevant documentation/scoring manuals.

Toxicity data (acute and late) will be analysed by comparing the proportion of patients by randomised treatment group using chi-squared test or Fisher's exact test as appropriate.

Proportions of patients requiring a feeding tube at 3, 12, 18 and 24 months after completion of treatment, experiencing a particular severe toxicity at a time point, ever experiencing a severe toxicity and suffering hearing loss at 12 months post radiotherapy and other binary endpoints will be compared between the two randomised groups using chi-squared or Fisher's exact test as appropriate.

The change in mean incisor distance between randomised groups will be compared by using a two-sample t-test or non-parametric Mann-Whitney test as appropriate, at specified time points.

Time to event endpoints will be displayed graphically using Kaplan-Meier plots, and randomised groups will be compared using log rank test and Cox proportional hazards models. Hazard ratios and Kaplan-Meier estimates of event rates will be reported with 95% confidence intervals.

Health economics analyses using EQ-5D-5L, healthcare resource-use, and WPAI-SHP data will be conducted by health economists and are outlined in Appendix A2.

17.4.3 Timing of analysis

The primary analysis will take place when all patients have completed 12 months post-treatment follow-up and will include analysis of some if not all secondary endpoints. Subsequent analyses (e.g. of late toxicity, longitudinal assessment of HR-QoL, loco-regional control and overall survival) are planned 2 and 5 years as the data mature.

17.5 Interim Analyses and Stopping Rules

Recruitment will be closely monitored by the Trial Management Group (TMG) with escalation to the independent Trial Steering Committee should it fall below 50% of target.

An Independent Data Monitoring Committee (IDMC) will review the accumulating data at least annually in confidence.

An initial review of the statistical assumptions underlying the power calculations for each of the co-primary endpoints will be undertaken after 25 control arm patients have been followed up for 12 months.
As the proposed duration of recruitment is 42 months, it is unlikely that sufficient data on the primary endpoint to inform an early stopping rule of the trial for efficacy or futility would be available by the time recruitment had been completed. Therefore no formal stopping rule based on the primary endpoint is proposed although a pre-planned review will take place when 12-month primary endpoint data are available for half the target sample size (estimated month 32 from start of recruitment). There is no suggestion that IMPT will be less effective than IMRT and given the pattern of recurrence seen in this disease setting, and the relatively short accrual period of the trial, any early stopping rule based on recurrence rates is likely to be based on limited information. In order to monitor recurrence rates, it is proposed that a sequential monitoring approach is adopted. Any loco-regional recurrences will be reported in an expedited fashion by the treating centre. The number of loco-regional recurrences out of the number of patients who have started trial treatment at that point will be tabulated by treatment group along with a p-value from Fisher's exact test. This will be sent to the IDMC, along with further details on the site of loco-regional recurrences reported and the IDMC will use this information along with any other emerging data to advise on early stopping of the trial.

18. TRIAL MANAGEMENT

18.1 Trial Management Group (TMG)

A Trial Management Group (TMG) will be set up and will include the Chief Investigator, ICR-CTSU Scientific Lead, Co-investigators and identified collaborators, the Trial Statistician, Clinical Trials Programme Manager and Trial Manager. Principal Investigators and key study personnel will be invited to join the TMG as appropriate to ensure representation from a range of sites and professional groups. Where possible, membership will include a lay/consumer representative. The TMG will meet at regular intervals, and at least annually. Notwithstanding the legal obligations of the Sponsor and Chief Investigator, the TMG have operational responsibility for the conduct of the trial. The Committee's terms of reference, roles and responsibilities will be defined in a charter issued by ICR-CTSU.

18.2 Trial Steering Committee (TSC)

A Trial Steering Committee (TSC) will be set up and will comprise an independent Chairman and at least two further independent members with clinical or statistical expertise (at least one member must be a statistician). The TSC will meet at regular intervals, and at least annually. The TSC will provide expert independent oversight of the trial on behalf of the Sponsor and funders. The Committee's terms of reference, roles and responsibilities will be defined in charter issued by ICR-CTSU.

18.3 Independent Data Monitoring Committee (IDMC)

An Independent Data Monitoring Committee (IDMC) will be set up to monitor the progress of the trial and will comprise a Chairman and at least two further members with clinical or statistical expertise (at least one member must be a statistician). Membership of the IDMC will be proposed by the TMG and approved by the TSC.

The IDMC will meet in confidence at regular intervals, and at least annually. A summary of findings and any recommendations will be produced following each meeting. This summary will be submitted to the TMG and TSC, and if required, the main REC.

The IDMC will reserve the right to release any data on outcomes or side-effects through the TSC to the TMG (and if appropriate to participants) if it determines at any stage that the combined evidence from this and other studies justifies it.

The Committee's terms of reference, roles and responsibilities will be defined in a charter issued by ICR-CTSU.

19. RESEARCH GOVERNANCE

19.1 Sponsor Responsibilities

The Sponsor of this clinical trial is the Institute of Cancer Research (ICR). The Sponsor is responsible for securing the arrangements to initiate, manage and finance the trial.

19.2 Participating Site Responsibilities

Responsibilities delegated to participating sites are defined in an agreement between the Sponsor and the individual site.

20. TRIAL ADMINISTRATION & LOGISTICS

20.1 Site activation

Before activating the trial, participating sites are required to sign an agreement accepting responsibility for all trial activity which takes place within their site.

Sites may commence recruitment once the site agreement has been signed by all required signatories, the required trial documentation is in place (as specified by ICR-CTSU) and a site initiation (visit or teleconference) has taken place. Site initiation visits will be conducted at sites where the Principal Investigator has requested one or where ICR-CTSU deems it is appropriate.

20.2 Investigator training

Each centre should complete the comprehensive pre-trial section of the radiotherapy quality assurance programme prior to commencing recruitment, as detailed in the TORPEdO Radiotherapy Planning and Delivery guidelines ("Quality Assurance (QA) pack"). In addition to this, and prior to commencing trial recruitment, training and advice will be provided via a trial launch meeting and/or training workshops, and

QA feedback to identified key individuals in each participating centre by members of the trial management group.

The Water Swallow Test should be performed by named SLTs. If this is not feasible within the time/resource constraints of the SLT clinic, a named trained research nurse may carry out the assessment. Evidence of competency of research nurses to perform the assessment must be provided to ICR-CTSU for review by SLT members of the TMG.

Participating centres will be asked to maintain a screening log to monitor randomisation acceptance rates, and additional support/training will be offered when lower than anticipated rates are encountered.

20.3 Data Acquisition

Electronic (e) Case Report Forms (CRF) will be used for the collection of trial data. ICR-CTSU will provide guidance to sites to aid the completion of the eCRFs. The Trial Management Group reserves the right to amend or add to the eCRF template as appropriate. Such changes do not constitute a protocol amendment, and revised or additional forms should be used by sites in accordance with the guidelines provided by ICR-CTSU. Patient Reported Outcomes will be collected using paper questionnaires.

Radiotherapy treatment DICOM data will also be collected. Guidelines on DICOM data submission will be given in the TORPEdO RTQA Pack.

20.4 Central Data Monitoring

Once data has been entered on the eCRF by the site personnel, ICR-CTSU will review it for compliance with the protocol, and for inconsistent or missing data. Should any missing data or data anomalies be found, queries will be raised for resolution by the site.

Any systematic inconsistencies identified through central data monitoring may trigger an on-site monitoring visit.

20.5 On-Site Monitoring

If a monitoring visit is required, ICR-CTSU will contact the site to arrange the visit. Once a date has been confirmed, the site should ensure that full patient notes of participants selected for source data verification are available for monitoring.

Appropriate internet access should be made available by the participating site for monitoring staff to enable review of eCRFs.

ICR-CTSU staff conducting on-site monitoring will review essential documentation and carry out source data verification to confirm compliance with the protocol. If any problems are detected during the course of the monitoring visit, ICR-CTSU will work with the Principal Investigator or delegated individual to resolve issues and determine appropriate action.

20.6 Completion of the Study and Definition of Study End Date

The study end date is deemed to be the date of last data capture.

20.7 Archiving

Essential trial documents should be retained according to local policy and for a sufficient period for possible inspection by the regulatory authorities (at least 5 years after the date of last data capture). Documents should be securely stored and access restricted to authorised personnel.

21. PATIENT PROTECTION AND ETHICAL CONSIDERATIONS

21.1 Risk Assessment and Approvals

This trial has been formally assessed for risk and approved by the Sponsor's Committee for Clinical Research.

21.2 Public and Patient Involvement

Patient advocate members were involved in protocol design including methodology, sample collection, patient information and consent forms and are represented on the TMG.

21.3 Ethics Approvals

The trial will not commence at any participating site until the required approvals are in place. ICR-CTSU, on behalf of the Sponsor, will ensure that the trial has received ethics approval from a research ethics committee (REC) for multi-centre trials, HRA approval and relevant NHS Permissions. Before recruiting patients, the Principal Investigator at each site is responsible for obtaining local approvals.

21.4 Trial Conduct

This trial will be conducted according to the approved protocol and its amendments, supplementary guidance and manuals supplied by the Sponsor and in accordance with the UK Policy Framework for Health and Social Care and the principles of GCP.

21.5 Informed consent

The Principal Investigator retains overall responsibility for the conduct of research at their site; this includes the taking of informed consent of participants. They must ensure that any person delegated responsibility to participate in the informed consent process is duly authorised, trained and competent to do so in accordance with the ethically approved protocol, principles of Good Clinical Practice and Declaration of Helsinki.

Patients should be asked to sign the current ethics approved TORPEdO consent form at trial entry after receiving both verbal and written information about the trial, having been given sufficient time to consider this information. All consent forms must be countersigned by the Principal Investigator or a designated individual. A signature log of delegated responsibilities, listing the designated individuals and the circumstances under which they may countersign consent forms, must be maintained at the participating

site. This log, together with original copies of all signed patient consent forms, should be retained in the Site Investigator File and must be available for inspection. The current ethics approved TORPEdO patient information sheets should be provided in addition to any standard patient information sheets that are provided by the site and used in routine practice

21.6 Patient Confidentiality

Patients will be asked to consent to their full name being collected at trial entry in addition to their date of birth, hospital number, postcode and NHS number or equivalent to allow linkage with routinely collected NHS data and ensure accuracy in handling biological samples.

Each investigator should keep a separate log of all participants' Trial IDs, names, addresses and hospital numbers. The investigator must retain trial documents (e.g. participants' written consent forms) in strict confidence. The investigator must ensure the participants' confidentiality is maintained at all times.

Representatives of ICR-CTSU will require access to participants' hospital notes for quality assurance purposes. ICR-CTSU will maintain the confidentiality of participants at all times and will not reproduce or disclose any information by which participants could be identified.

21.7 Data Protection

All investigators and trials staff must comply with applicable data protection laws at all times.

21.8 Liability

Indemnity to meet the potential legal liability of investigators participating in this trial is provided by the usual NHS indemnity arrangements.

22. FINANCIAL MATTERS

This trial is investigator designed and led. ICR has received funding from Cancer Research UK (CRUK/18/010) for the central coordination of the trial. Additional financial support for the study has been obtained from the Taylor Family Foundation. The trial meets the criteria for R&D support as outlined in the Statement of Partnership on Non-Commercial R&D in the NHS in England. The trial is part of the National Institute for Health Research Clinical Research Network (NIHR CRN) portfolio by virtue of its funding by the NIHR non-commercial partner. NIHR CRN resources should therefore be made available for the trial to cover UK specific research costs. NHS England as commissioners of Proton Beam Therapy services are fully supportive of TORPEdO.

23. PUBLICATION POLICY

The main trial results will be published in a peer-reviewed journal, on behalf of all collaborators. The manuscript will be prepared by a writing group, consisting of members of the TMG. Participating clinicians may be selected to join the writing group on the basis of intellectual and time input. All participating clinicians will be acknowledged in the publication.

Any presentations and publications relating to the trial must be authorised by the TMG. Authorship of any secondary publications e.g. those relating to sub-studies, will reflect intellectual and time input into these studies. Authorship of all publication will usually be in accordance with ICMJE guidance.

No investigator may present or attempt to publish data relating to the TORPEdO trial without prior permission from the TMG.

24. ASSOCIATED STUDIES

24.1 Health Economics Study

A within-trial economic component will be integrated into the study to estimate the cost-effectiveness of IMPT from the perspective of the UK NHS, patient and society. Healthcare resource use will be collected from hospital electronic patient records as well as via patient or family member self-report using a participant resource-use questionnaire developed specifically for the study. The development of the participant resource-use questionnaire will be informed by PPI representatives included within the study research team to capture wider costs to the patient and family of centrally-delivered radiotherapy services (i.e. travel costs, informal carer support and loss of earnings). Patient resource use from the date of randomisation to the end of 1-year follow-up will be used to define cumulative healthcare costs for the within-trial economic component (see Appendix A2).

24.2 Imaging and physics based translational study

- Pre-accrual (benchmarking) and during-accrual (recruited patient) DICOM-RT and associated non DICOM data will be collected to validate and develop improved NTCP models and refine patient selection for IMPT.

The collection of all pre-accrual (benchmarking) and during-accrual (recruited patient) DICOM-RT and associated non DICOM data (RTTQA, funded by a central NIHR grant) will underpin future studies to validate and develop improved NTCP models, refine patient selection for IMPT and explore the following hypotheses:

- Correlation of image-based data mining DICOM data and per-voxel RT doses with continuous toxicity variables (e.g., trismus, audiometry, lymphopenia) will improve dose constraints for normal tissue sub-structures.

- Data from weekly cone beam CT images and the repeat verification CT scan will increase understanding of how often to adapt IMPT and IMRT plans and allow development of meaningful thresholds and a clinical traffic light protocol for adaptation.

24.3 Biological sub study

The TORPEdO biological sub study will be co-ordinated by the Manchester Cancer Research Centre at the University of Manchester.

TORPEdO provides an opportunity to collect samples and data for translational research to inform the design of future biomarker driven trials aimed at the optimal selection of IMPT *vs* IMRT based not only on

NTCP models but also tumour and normal tissue genomics. Due to differences in the more complex nature of the DNA damage produced by protons *vs* photons, the following hypotheses are made:

- Patients with tumours exhibiting a defective DNA damage response (DDRD) profile associated with defects in homologous recombination will benefit more from protons vs photons.
- There will be some non-overlapping genetic variants that increase the risk of toxicity to protons vs photons.
- Protons will generate an enhanced immune response compared with photons that can be detected in HPV-associated oropharyngeal cancers due to their heightened immune response.

The short half-life of circulating free DNA (cfDNA) and ease of longitudinal sampling enables its use for real-time monitoring of cancer burden in response to therapy. A reduction of cfDNA CNV to 5% from baseline is considered a response. Methylation patterns of cfDNA indicate recent cell death events in the body linkable to tissue origin via a reference methylation atlas of human tissues and cell types. Both parameters could be early response indicators and endpoints that would shorten the follow-up required in subsequent trials. Therefore additional exploratory studies can address the following hypotheses:

- A quantitative reduction in the percentage of plasma cfDNA copy number variation (CNV) will provide an early measure of tumour response to treatment.
- A quantitative change in cfDNA methylation from baseline to 3 months post radiotherapy will be an early indicator of late normal tissue damage.

Prestin is a unique inner ear protein that is released when outer hair cells are injured. The severity of hearing loss has been correlated with changes in levels of prestin circulating in the blood. Therefore it is also hypothesised that:

- Early changes in plasma prestin levels will predict risk of treatment induced hearing loss.

24.3.1 Sample Collection & Logistics (Further details are given in the TORPEdO Sample Collection Manual)

At the time of randomisation all patients will be asked to consent to donate:

- a formalin-fixed paraffin-embedded (FFPE) diagnostic tumour tissue sample.
- whole blood samples at baseline, during and after treatment up to the 3 month (post treatment) timepoints as detailed in Section 12 (Schedule of assessments).

The collection of gifted tissue and blood samples will be used to measure changes in cfDNA and also provides a biorepository for use in future translational work (subject to ethical approval) without the need to re consent patients at a later date. If a patient decides to withdraw from the TORPEdO trial their samples will continue be stored for use in this and future translational research, unless they explicitly make it known that they would like their samples to be destroyed.

The Manchester Cancer Research Centre will provide ALL centres with the blood collection supplies. Blood samples should be taken according to the schedule in section 12. The research blood samples are collected at the same time as clinical bloods to reduce the number of needle pricks.

On receipt, all blood samples are bar-coded, logged and stored in monitored -80°C freezers on HTA licensed premises (Manchester Cancer Research Centre Manchester; HTA licence number 3004).

Block samples will be stored at room temperature at the University of Manchester where sections will be cut by histology for analysis and if material is left over, blocks will be returned to the original hospital.

24.4 Diffusion weighted (DW) MRI substudy

24.4.1 Background

MR imaging is increasing in availability, and there is considerable interest in the role of MRI in identifying imaging biomarkers to predict clinical outcomes based on tumour factors (57) and changes in organs at risk (OAR) (58), and ultimately to allow clinicians to change their radiotherapy approach (i.e. amending target / organ at risk outlines and/or changing the prescribed dose) during treatment.

Diffusion-weighted imaging (DWI) relates to the diffusion of water within volumes of interest and is quantified by the apparent diffusion co-efficient (ADC). There is limited work relating changes in the ADC in the major salivary glands of the head and neck – the parotid (PG) and submandibular (SMG) glands with subsequent toxicity. Zhang et al (59) described a series of 26 patients with nasopharyngeal carcinoma who underwent DW-MRI 1 week before radiotherapy, and after 2 weeks of radiotherapy. The resting ADC was significantly higher in both the PG and SMG at 2 weeks, and the change in increased ADC was associated with the degree of xerostomia at 6 months following treatment. Other studies have shown a relationship between the dose received by an OAR and changes in functional MRI characteristics but the correlation with subsequent toxicity is poorly described (58).

The limited current literature pertains to patients treated with photon-based radiation rather than proton therapy. This study offers a unique opportunity to assess changes in ADC for patients treated with both modalities, and allows the opportunity to explore differences between each. Recruiting patients from TORPEdO will allow for high-quality toxicity data (clinician and patient-reported) to be correlated with imaging changes.

24.4.2 Design

This is a prospective (sub) study which will be initially performed in approximately 46 patients enrolled on the TORPEdO trial and randomised to either IMPT or IMRT at the Christie (Manchester). The sub-study will be in 2 stages. In stage 1, patients recruited to the sub-study will also undergo functional MRI sequences at baseline. In stage 2, patients will also undergo functional MRI sequences at baseline and also during week 3 of treatment.

24.4.3 Primary objectives

Determine a threshold change in ADC on DW-MRI imaging of OAR between baseline and week 3 of treatment that can predict patients that experience severe toxicity.

24.4.4 Secondary objectives

Identify the optimum MRI sequences for adaptive planning.
Assess the relative effects of proton- and photon-based treatment on OAR as described by dose received, functional MRI parameters and toxicity measures.

Evaluate treatment response, date of relapse, progression or death

24.4.5 Data acquisition and storage

Patients will be scanned in a supine position, and will be immobilised in a 5-point thermoplastic shell. A peripheral venous cannula will be inserted in the upper limb and gadolinium contrast injected prior to scanning. T1, T2, DW and dynamic contrast-enhanced (DCE) sequences will be obtained.

Analysis of images will be performed via well-established in-house protocols at the University of Manchester, as part of the CRUK National Translational Cancer Imaging Network. Imaging data will be stored on a file server that is accessible only from within the University of Manchester by authorized personnel only. Further processing of imaging data will be strictly done after anonymisation by providing an ID code and removing all personal information from image file headers. Stored MRI scans will not allow identification of subjects. Data held on laptop computers will be anonymised, encrypted and password protected and will be uploaded onto a secure desktop as soon as possible and the data will be removed from the portable device using appropriate data destruction software. Completed consent forms and CRFs will be stored at the Christie in a secure location.

24.5 Translational biopsy sub study - Assessing Changes in the Tumour Microenvironment for Patients with Oropharynx Cancer treated with Chemo-Radiation

24.5.1 Background

The Tumour Microenvironment

Radiotherapy is increasingly understood to have both immune stimulatory and immune suppressive effects within the local tumour microenvironment (TME). (60) Furthermore, the immunological makeup of the TME has been shown to be a predictive biomarker of response to (chemo) RT in head and neck squamous cell carcinoma (HNSCC). Analysis of the immune composition of the TME has shown that high baseline levels of CD8+ (cytotoxic) T-cells (61) and PD-L1 expression on tumour cells Fukushima (62) are associated with improved survival.

Pre-clinical models have demonstrated a number of RT-induced immune stimulatory effects, including induction of immunogenic cell death (which can lead to activation of antigen presenting cells and priming of T-cell activation), production of type I interferons, upregulation of MHC class I molecules and increased expression of adhesion molecules and chemokines, leading to increased T cell infiltration. Conversely, immuno suppressive effects can induce pro-inflammatory cytokines and chemokines leading to recruitment of myeloid derived suppressor cells, macrophages and regulatory T cells and polarisation of macrophages and monocytes from a tumour killing M1 phenotype to a tumour promoting M2 phenotype. Colton (63)

Additional pre-clinical models have shown that RT can influence the composition of the tumour immune microenvironment with changes in the numbers of infiltrating T cells and myeloid cells. RT is able to increase the number of tumour infiltrating T cells which in turn causes the upregulation of the immune checkpoint inhibitor PD-L1 on tumour cells. For these tumours, combining RT with immune checkpoint inhibitors (ICIs) leads to induction of systemic immunity and tumour clearance. Dovedi (64) However, other murine tumours lack T cells and have high numbers of infiltrating suppressive myeloid cells. For these tumours, combining RT with ICIs is unsuccessful and combination therapy with immune stimulatory agents (e.g. TLR agonists, agonistic anti CD40 antibody) is required instead. Thus, profiling of the tumour immune

microenvironment is critical, not just to identify possible biomarkers of response to RT, but to determine whether it is possible to predict which patients may be suitable for future combination therapies with ICIs and which might require combination therapy with other immune stimulatory agents. This enhanced understanding is crucial for the development of future RT/ immuno oncology agent combination trials to improve the outcomes of patients with HNSCC.

In this prospective sub study, patients with node-positive OPSCC randomised to either IMRT or IMPT will be given the option to consent to two additional core biopsies of involved lymph nodes, at baseline and during week 3 of treatment. Multiplex immunohistochemistry and immune gene signatures will be used to analyse changes in the immune TME. From an immunological perspective, we will test the hypotheses that an enhanced immune response is generated (i) for patients treated with IMPT due to more complex DNA damage produced compared to IMRT and (ii) for patients with p16 positive disease compared to p16 negative disease. Correlation of TME features with tumour hypoxia, defined by the West gene signature, will allow testing of the hypothesis that hypoxia is associated with immunosuppression.

Following analysis, it is hoped that an immune signature can be developed that correlates with treatment response on post treatment imaging (e.g., FDG PET CT) and survival endpoints to allow the early identification of patients that may benefit from intensified treatment, e.g. intra-tumoural injection of a toll like receptor agonist Furthermore, it is hoped that these immune signatures may be used to predict which immunotherapy agents to use for intensified treatment which would be tested in future clinical trials.

The rationale for sampling involved lymph nodes
The response of involved lymph nodes to therapy is a paradigm for global treatment response in oropharynx cancer; large and / or necrotic lymph nodes may not respond completely to (chemo) RT and a salvage neck dissection is required in approximately 20% of cases (65). Lymph nodes are typically easier to access and sample when compared to primary tumours and procedures can be performed with ultrasound guidance without the need for operating theatre capacity. Importantly, patient acceptability is likely to be higher, with previous attendees at Patient and Public Involvement Exercise (PPIE) sessions indicating that they would be prepared to undergo additional biopsies, but expressed concern regarding the tolerability of primary site biopsy. Further, studying involved neck nodes may align with future trials where patients treated with a de-escalated radiotherapy regimen undergo scheduled neck dissection.

24.5.2 Design
This is a prospective sub study which will be performed in 50 patients with oropharynx cancer and involved cervical lymph nodes (i.e., p16 positive stage N1-2 or p16 negative N1-2c) enrolled on the TORPEdO trial and randomised to either IMPT or IMRT at The Christie, Manchester.

Consenting patients will undergo an ultrasound (US)-guided core biopsy of an involved node at baseline (i.e., at a time point between enrolment in TORPEdO and prior to commencing treatment) and during week three of chemo radiation. Biopsies will be performed in the radiology department at The Christie NHS Foundation Trust by an experienced specialist head and neck radiologist. Biopsies will be performed using ultrasound guidance and following the administration of local anaesthetic.

24.5.3 Inclusion criteria

Patients enrolled in the TORPEdO trial with cervical lymph node involvement, i.e. p16 positive N1-2 or p16 negative N1-2c

2. An involved lymph node > 2cm in diameter and considered technically accessible for ultrasound-guided biopsy

3. Adequate full blood count and clotting screen parameters (i.e., platelet count > 100 and prothrombin time < 14 seconds)

24.5.4 Primary objective

To collect biopsies of an involved neck node at baseline and during treatment to look for immunological changes in the tumour microenvironment.

24.5.5 Exploratory objectives

- IHC analysis of marker expression (including immunological markers) on tumour tissue from biopsies taken pre and during week 3 of IMRT / IMPT.
- RNA or DNA evaluation of immune signatures in tumour tissue from biopsies taken pre and during week 3 of IMRT / IMPT.
- T-Cell Receptor (TCR) repertoire sequencing of tumour tissue and comparison with TCR repertoire in the blood.
- DNA sequencing for whole exome sequencing for tumour mutational burden
- Correlate changes in the TME with immune blood tests
- Assess for differences in the TME between patients treated with IMPT and IMRT
- Assess for differences in the TME between p16 positive and p16 negative oropharynx cancer
- Correlate TME immune response with tumour hypoxia

24.5.6 Laboratory analysis

Biopsy samples will be collected from the radiology department at The Christie. One sample will be taken to the Christie Pathology department for fixation in formalin and transfer to paraffin embedded blocks (FFPE) before being sent to the Targeted Therapy Group (TTG) laboratory (University of Manchester). The second sample will be immersed in AllProtect tissue reagent and transported directly to the TTG laboratory.

Samples will be analysed to determine a correlation between findings in the primary tumour and associated involved lymph nodes and to look at dynamic changes in the involved lymph nodes during radiotherapy. Tumour tissue will be analysed by techniques including:

- Immunohistochemistry to identify changes in the immune contexture following radiotherapy
- RNA gene expression arrays to identify changes in immune gene signatures
- DNA and RNA sequencing of tumour tissue to investigate tumour mutational burden and T cell repertoire
- Cytokine and chemokine analysis of tumour

24.5.7 Statistical analysis

This sub study is a pilot exploratory study investigating whether there are dynamic changes in the tumour immune microenvironment with radiotherapy. As such it is not possible to power the study to determine sample size. As such, all statistics will be descriptive in nature. For analysis of laboratory data statistical analysis will be performed using R or Graph Pad Prism or other appropriate statistical packages.

24.5.8 Data storage

Data will be stored on a file server that is accessible only from within the University of Manchester by authorized personnel only. Study team records e.g. completed consent forms and CRFs will be stored at the Christie in a secure location.

References

1. Office for National Statistics. Cancer Registration Statistics EsM, No44 2013. 2018.
2. Schache AG, Powell NG, Cuschieri KS, Robinson M, Leary S, Mehanna H, et al. HPV-Related Oropharynx Cancer in the United Kingdom: An Evolution in the Understanding of Disease Etiology. Cancer Res. 2016;76(22):6598-606.
3. Ang KK, Harris J, Wheeler R, Weber R, Rosenthal DI, Nguyen-Tan PF, et al. Human papillomavirus and survival of patients with oropharyngeal cancer. N Engl J Med. 2010;363(1):24-35.
4. Mehanna H, Evans M, Beasley M, Chatterjee S, Dilkes M, Homer J, et al. Oropharyngeal cancer: United Kingdom National Multidisciplinary Guidelines. J Laryngol Otol. 2016;130(S2):S90-S6.
5. Nutting CM, Morden JP, Harrington KJ, Urbano TG, Bhide SA, Clark C, et al. Parotid-sparing intensity modulated versus conventional radiotherapy in head and neck cancer (PARSPORT): a phase 3 multicentre randomised controlled trial. Lancet Oncol. 2011;12(2):127-36.
6. van der Laan HP, Bijl HP, Steenbakkers RJ, van der Schaaf A, Chouvalova O, Vemer-van den Hoek JG, et al. Acute symptoms during the course of head and neck radiotherapy or chemoradiation are strong predictors of late dysphagia. Radiother Oncol. 2015;115(1):56-62.
7. Langendijk JA, Doornaert P, Verdonck-de Leeuw IM, Leemans CR, Aaronson NK, Slotman BJ. Impact of late treatment-related toxicity on quality of life among patients with head and neck cancer treated with radiotherapy. J Clin Oncol. 2008;26(22):3770-6.
8. Machtay M, Moughan J, Trotti A, Garden AS, Weber RS, Cooper JS, et al. Factors associated with severe late toxicity after concurrent chemoradiation for locally advanced head and neck cancer: an RTOG analysis. J Clin Oncol. 2008;26(21):3582-9.
9. Setton J, Lee NY, Riaz N, Huang SH, Waldron J, O'Sullivan B, et al. A multi-institution pooled analysis of gastrostomy tube dependence in patients with oropharyngeal cancer treated with definitive intensity-modulated radiotherapy. Cancer. 2015;121(2):294-301.
10. Wopken K, Bijl HP, Langendijk JA. Prognostic factors for tube feeding dependence after curative (chemo-) radiation in head and neck cancer: A systematic review of literature. Radiother Oncol. 2018;126(1):56-67.
11. Mehta S YK, Mcpartlin A, Garcez K, Sykes A, Thomson D, et al. Feeding tube dependency at 12 months post treatment in oropharyngeal cancer patient treated with radical intent- a single institute experience. Eur J Surg Oncol. 2018;Volume 44(Supplement 1):S45-S6.
12. Dixon L, Ramasamy S, Cardale K, Dyker K, Garcez K, Lee LW, et al. Long term patient reported swallowing function following chemoradiotherapy for oropharyngeal carcinoma. Radiother Oncol. 2018.
13. Lowe D RS. University of Washington Quality of Life Questionnaire (UW-QOL v4 and v4.1): Guidance for scoring and presentation. 2018.
14. Hoxbroe Michaelsen S, Gronhoj C, Hoxbroe Michaelsen J, Friborg J, von Buchwald C. Quality of life in survivors of oropharyngeal cancer: A systematic review and meta-analysis of 1366 patients. Eur J Cancer. 2017;78:91-102.
15. Hawkins PG, Lee JY, Mao Y, Li P, Green M, Worden FP, et al. Sparing all salivary glands with IMRT for head and neck cancer: Longitudinal study of patient-reported xerostomia and head-and-neck quality of life. Radiother Oncol. 2018;126(1):68-74.
16. Vainshtein JM, Samuels S, Tao Y, Lyden T, Haxer M, Spector M, et al. Impact of xerostomia on dysphagia after chemotherapy-intensity-modulated radiotherapy for oropharyngeal cancer: Prospective longitudinal study. Head Neck. 2016;38 Suppl 1:E1605-12.
17. Gulliford SL, Miah AB, Brennan S, McQuaid D, Clark CH, Partridge M, et al. Dosimetric explanations of fatigue in head and neck radiotherapy: an analysis from the PARSPORT Phase III trial. Radiother Oncol. 2012;104(2):205-12.
18. Barten DL, Tol JP, Dahele M, Slotman BJ, Verbakel WF. Comparison of organ-at-risk sparing and plan robustness for spot-scanning proton therapy and volumetric modulated arc photon therapy in head-and-neck cancer. Med Phys. 2015;42(11):6589-98.

19. Thomson DJ, Teo B-KK, Ong A, Ang KW, Kirk M, Ahn PH, et al. The Impact of Anatomic Change on Pencil Beam Scanning in the Treatment of Oropharynx Cancer. International Journal of Particle Therapy. 2015;2(2):394-403.
20. Holliday EB, Kocak-Uzel E, Feng L, Thaker NG, Blanchard P, Rosenthal DI, et al. Dosimetric advantages of intensity-modulated proton therapy for oropharyngeal cancer compared with intensity-modulated radiation: A case-matched control analysis. Med Dosim. 2016;41(3):189-94.
21. Simone CB, 2nd, Ly D, Dan TD, Ondos J, Ning H, Belard A, et al. Comparison of intensity-modulated radiotherapy, adaptive radiotherapy, proton radiotherapy, and adaptive proton radiotherapy for treatment of locally advanced head and neck cancer. Radiother Oncol. 2011;101(3):376-82.
22. Apinorasethkul O, Kirk M, Teo K, Swisher-McClure S, Lukens JN, Lin A. Pencil beam scanning proton therapy vs rotational arc radiation therapy: A treatment planning comparison for postoperative oropharyngeal cancer. Med Dosim. 2017;42(1):7-11.
23. Jakobi A, Bandurska-Luque A, Stutzer K, Haase R, Lock S, Wack LJ, et al. Identification of Patient Benefit From Proton Therapy for Advanced Head and Neck Cancer Patients Based on Individual and Subgroup Normal Tissue Complication Probability Analysis. Int J Radiat Oncol Biol Phys. 2015;92(5):1165-74.
24. Gunn GB, Blanchard P, Garden AS, Zhu XR, Fuller CD, Mohamed AS, et al. Clinical Outcomes and Patterns of Disease Recurrence After Intensity Modulated Proton Therapy for Oropharyngeal Squamous Carcinoma. Int J Radiat Oncol Biol Phys. 2016;95(1):360-7.
25. Sio TT, Lin HK, Shi Q, Gunn GB, Cleeland CS, Lee JJ, et al. Intensity Modulated Proton Therapy Versus Intensity Modulated Photon Radiation Therapy for Oropharyngeal Cancer: First Comparative Results of Patient-Reported Outcomes. Int J Radiat Oncol Biol Phys. 2016;95(4):1107-14.
26. Blanchard P, Garden AS, Gunn GB, Rosenthal DI, Morrison WH, Hernandez M, et al. Intensity-modulated proton beam therapy (IMPT) versus intensity-modulated photon therapy (IMRT) for patients with oropharynx cancer - A case matched analysis. Radiother Oncol. 2016;120(1):48-55.
27. Zhang W, Zhang X, Yang P, Blanchard P, Garden AS, Gunn B, et al. Intensity-modulated proton therapy and osteoradionecrosis in oropharyngeal cancer. Radiother Oncol. 2017;123(3):401-5.
28. Garden AS, Dong L, Morrison WH, Stugis EM, Glisson BS, Frank SJ, et al. Patterns of disease recurrence following treatment of oropharyngeal cancer with intensity modulated radiation therapy. Int J Radiat Oncol Biol Phys. 2013;85(4):941-7.
29. Beitler JJ, Chera BS. Protons for Oropharyngeal Cancer Have Not Yet Justified Their Promise. Int J Radiat Oncol Biol Phys. 2016;95(4):1115-6.
30. Langendijk JA, Lambin P, De Ruysscher D, Widder J, Bos M, Verheij M. Selection of patients for radiotherapy with protons aiming at reduction of side effects: the model-based approach. Radiother Oncol. 2013;107(3):267-73.
31. Bijman RG, Breedveld S, Arts T, Astreinidou E, de Jong MA, Granton PV, et al. Impact of model and dose uncertainty on model-based selection of oropharyngeal cancer patients for proton therapy. Acta Oncol. 2017;56(11):1444-50.
32. Arts T, Breedveld S, de Jong MA, Astreinidou E, Tans L, Keskin-Cambay F, et al. The impact of treatment accuracy on proton therapy patient selection for oropharyngeal cancer patients. Radiother Oncol. 2017;125(3):520-5.
33. Shaw SM, Flowers H, O'Sullivan B, Hope A, Liu LW, Martino R. The effect of prophylactic percutaneous endoscopic gastrostomy (PEG) tube placement on swallowing and swallow-related outcomes in patients undergoing radiotherapy for head and neck cancer: a systematic review. Dysphagia. 2015;30(2):152-75.
34. Perry A, Lee SH, Cotton S, Kennedy C. Therapeutic exercises for affecting post-treatment swallowing in people treated for advanced-stage head and neck cancers. Cochrane Database Syst Rev. 2016(8):CD011112.
35. Skalleberg J, Smastuen MC, Oldenburg J, Osnes T, Fossa SD, Bunne M. The Relationship Between Cisplatin-related and Age-related Hearing Loss During an Extended Follow-up. Laryngoscope. 2020.
36. Whitmer WM, Howell P, Akeroyd MA. Proposed norms for the Glasgow hearing-aid benefit profile (Ghabp) questionnaire. Int J Audiol. 2014;53(5):345-51.

37. Killion MC, Niquette PA. What can the pure-tone audiogram tell us about a patient's SNR loss? The Hearing Journal. 2000;53(3):46-8, 50, 2-3.
38. Brouwer CL, Steenbakkers RJ, van der Schaaf A, Sopacua CT, van Dijk LV, Kierkels RG, et al. Selection of head and neck cancer patients for adaptive radiotherapy to decrease xerostomia. Radiother Oncol. 2016;120(1):36-40.
39. Radiologists TRCo. Timely delivery of radical radiotherapy: guidelines for the management of unscheduled treatment interruptions. 2019;Fourth edition.
40. Roe JW, Drinnan MJ, Carding PN, Harrington KJ, Nutting CM. Patient-reported outcomes following parotid-sparing intensity-modulated radiotherapy for head and neck cancer. How important is dysphagia? Oral Oncol. 2014;50(12):1182-7.
41. Rwigema JC LJ, van der Laan HP, Lukens JN, Swisher-McClure SD, Lin A. Proton Therapy Improves Toxicity for Oropharyngeal Cancer : An Outcomes and Predictive Model-based Approach. International Journal of Radiation Oncology Biology Physics. 2017;99(2):S135-S6.
42. Sharma S ZO, Thompson RF, Lukens JN, Lin A, Ahn P A Comparative Study of Patient-Reported Xerostomia and Dysgeusia in Oropharyngeal Squamous Cell Carcinoma (OPSCC) Treated with Volumetric Modulated Arc Therapy (VMAT) or Proton Pencil Beam Scanning (PBS). International Journal of Radiation Oncology, Biology, Physics 2015;93(3S):S69.
43. Sharma S ZO, Thompson RF, Lukens JN, Lin A, Ahn P Correlation of Dosimetric Parameters to Patient-Reported Taste Changes After Radiation for Oropharyngeal Squamous Cell Carcinoma. International Journal of Radiation Oncology, Biology, Physics 2016;96(2):E354.
44. Hassan SJ, Weymuller EA, Jr. Assessment of quality of life in head and neck cancer patients. Head Neck. 1993;15(6):485-96.
45. Rogers SN, Lowe D, Yueh B, Weymuller EA, Jr. The physical function and social-emotional function subscales of the University of Washington Quality of Life Questionnaire. Arch Otolaryngol Head Neck Surg. 2010;136(4):352-7.
46. EORTC QLQ-C30.
47. EORTC QLQ-H&N43.
48. Singer S, Amdal CD, Hammerlid E, Tomaszewska IM, Castro Silva J, Mehanna H, et al. International validation of the revised European Organisation for Research and Treatment of Cancer Head and Neck Cancer Module, the EORTC QLQ-HN43: Phase IV. Head Neck. 2019;41(6):1725-37.
49. Singer S, Araujo C, Arraras JI, Baumann I, Boehm A, Brokstad Herlofson B, et al. Measuring quality of life in patients with head and neck cancer: Update of the EORTC QLQ-H&N Module, Phase III. Head Neck. 2015;37(9):1358-67.
50. Chen AY, Frankowski R, Bishop-Leone J, Hebert T, Leyk S, Lewin J, et al. The development and validation of a dysphagia-specific quality-of-life questionnaire for patients with head and neck cancer: the M. D. Anderson dysphagia inventory. Arch Otolaryngol Head Neck Surg. 2001;127(7):870-6.
51. Patterson JM, Hildreth A, McColl E, Carding PN, Hamilton D, Wilson JA. The clinical application of the 100mL water swallow test in head and neck cancer. Oral Oncol. 2011;47(3):180-4.
52. Performance Status Scale for Head & Neck Cancer Patients (PSS-HN).
53. List MA, Ritter-Sterr C, Lansky SB. A performance status scale for head and neck cancer patients. Cancer. 1990;66(3):564-9.
54. Work Productivity and Activity Impairment Questionnaire: Specific Health Problem V2.0 (WPAI:SHP).
55. Reilly MC, Zbrozek AS, Dukes EM. The validity and reproducibility of a work productivity and activity impairment instrument. PharmacoEconomics. 1993;4(5):353-65.
56. EQ-5D.
57. Martens RM, Noij DP, Ali M, Koopman T, Marcus JT, Vergeer MR, et al. Functional imaging early during (chemo)radiotherapy for response prediction in head and neck squamous cell carcinoma; a systematic review. Oral Oncol. 2019;88:75-83.
58. Stieb S, Elgohari B, Fuller CD. Repetitive MRI of organs at risk in head and neck cancer patients undergoing radiotherapy. Clinical and translational radiation oncology. 2019;18:131-9.

59. Zhang Q, Wei YM, Qi YG, Li BS. Early Changes in Apparent Diffusion Coefficient for Salivary Glands during Radiotherapy for Nasopharyngeal Carcinoma Associated with Xerostomia. Korean journal of radiology. 2018;19(2):328-33.
60. Honeychurch J, Illidge TM. The influence of radiation in the context of developing combination immunotherapies in cancer. Therapeutic Advances in Vaccines and Immunotherapy. 2017;5(6):115-22.
61. Balermpas P, Michel Y, Wagenblast J, Seitz O, Weiss C, Rödel F, et al. Tumour-infiltrating lymphocytes predict response to definitive chemoradiotherapy in head and neck cancer. British Journal of Cancer. 2014;110(2):501-9.
62. Fukushima Y, Someya M, Nakata K, Hori M, Kitagawa M, Hasegawa T, et al. Influence of PD-L1 expression in immune cells on the response to radiation therapy in patients with oropharyngeal squamous cell carcinoma. Radiother Oncol. 2018;129(2):409-14.
63. Colton M, Cheadle EJ, Honeychurch J, Illidge TM. Reprogramming the tumour microenvironment by radiotherapy: implications for radiotherapy and immunotherapy combinations. Radiation Oncology. 2020;15(1):254.
64. Dovedi SJ, Adlard AL, Lipowska-Bhalla G, McKenna C, Jones S, Cheadle EJ, et al. Acquired Resistance to Fractionated Radiotherapy Can Be Overcome by Concurrent PD-L1 Blockade. Cancer Research. 2014;74(19):5458-68.
65. Mehanna H, Wong WL, McConkey CC, Rahman JK, Robinson M, Hartley AG, et al. PET-CT Surveillance versus Neck Dissection in Advanced Head and Neck Cancer. N Engl J Med. 2016;374(15):1444-54.
66. Beetz I, Schilstra C, van der Schaaf A, van den Heuvel ER, Doornaert P, van Luijk P, et al. NTCP models for patient-rated xerostomia and sticky saliva after treatment with intensity modulated radiotherapy for head and neck cancer: the role of dosimetric and clinical factors. Radiother Oncol. 2012;105(1):101-6.
67. Wopken K, Bijl HP, van der Schaaf A, van der Laan HP, Chouvalova O, Steenbakkers RJ, et al. Development of a multivariable normal tissue complication probability (NTCP) model for tube feeding dependence after curative radiotherapy/chemo-radiotherapy in head and neck cancer. Radiother Oncol. 2014;113(1):95-101.
68. Kierkels RGJ, Wopken K, Visser R, Korevaar EW, van der Schaaf A, Bijl HP, et al. Multivariable normal tissue complication probability model-based treatment plan optimization for grade 2-4 dysphagia and tube feeding dependence in head and neck radiotherapy. Radiother Oncol. 2016;121(3):374-80.
69. Vergouwe Y, Nieboer D, Oostenbrink R, Debray TPA, Murray GD, Kattan MW, et al. A closed testing procedure to select an appropriate method for updating prediction models. Stat Med. 2017;36(28):4529-39.
70. Wilson JA, Carding PN, Patterson JM. Dysphagia after nonsurgical head and neck cancer treatment: patients' perspectives. Otolaryngology--head and neck surgery : official journal of American Academy of Otolaryngology-Head and Neck Surgery. 2011;145(5):767-71.
71. Patterson JM, McColl E, Carding PN, Hildreth AJ, Kelly C, Wilson JA. Swallowing in the first year after chemoradiotherapy for head and neck cancer: Clinician-and patient-reported outcomes. Head Neck. 2013.
72. PATHOS. https://www.clinicaltrials.gov/ct2/show/NCT02215265?term=pathos&rank=1.
73. De-ESCALaTE. https://www.clinicaltrials.gov/ct2/show/NCT01874171?term=de-escalate&rank=1.
74. Moor JW, Patterson J, Kelly C, Paleri V. Prophylactic gastrostomy before chemoradiation in advanced head and neck cancer: a multiprofessional web-based survey to identify current practice and to analyse decision making. Clin Oncol (R Coll Radiol). 2010;22(3):192-8.
75. Patterson JM, McColl E, Carding PN, Kelly C, Wilson JA. Swallowing performance in patients with head and neck cancer: a simple clinical test. Oral Oncol. 2009;45(10):904-7.
76. List MA, Mumby P, Haraf D, Siston A, Mick R, MacCracken E, et al. Performance and quality of life outcome in patients completing concomitant chemoradiotherapy protocols for head and neck cancer. Quality of life research : an international journal of quality of life aspects of treatment, care and rehabilitation. 1997;6(3):274-84.

77. List MA, Siston A, Haraf D, Schumm P, Kies M, Stenson K, et al. Quality of life and performance in advanced head and neck cancer patients on concomitant chemoradiotherapy: a prospective examination. J Clin Oncol. 1999;17(3):1020-8.
78. Porceddu SV, Pryor DI, Burmeister E, Burmeister BH, Poulsen MG, Foote MC, et al. Results of a prospective study of positron emission tomography-directed management of residual nodal abnormalities in node-positive head and neck cancer after definitive radiotherapy with or without systemic therapy. Head Neck. 2011;33(12):1675-82.
79. Sjovall J, Wahlberg P, Almquist H, Kjellen E, Brun E. A prospective study of positron emission tomography for evaluation of neck node response 6 weeks after radiotherapy in patients with head and neck squamous cell carcinoma. Head Neck. 2016;38 Suppl 1:E473-9.
80. Bird T, Barrington S, Thavaraj S, Jeannon JP, Lyons A, Oakley R, et al. (18)F-FDG PET/CT to assess response and guide risk-stratified follow-up after chemoradiotherapy for oropharyngeal squamous cell carcinoma. Eur J Nucl Med Mol Imaging. 2016;43(7):1239-47.
81. Nelissen C, Sherriff J, Jones T, Guest P, Colley S, Sanghera P, et al. The Role of Positron Emission Tomography/Computed Tomography Imaging in Head and Neck Cancer after Radical Chemoradiotherapy: a Single Institution Experience. Clin Oncol (R Coll Radiol). 2017;29(11):753-9.
82. Vainshtein JM, Spector ME, Stenmark MH, Bradford CR, Wolf GT, Worden FP, et al. Reliability of post-chemoradiotherapy F-18-FDG PET/CT for prediction of locoregional failure in human papillomavirus-associated oropharyngeal cancer. Oral Oncol. 2014;50(3):234-9.
83. Prestwich RJ, Sen M, Scarsbrook AF. Management of Advanced Head and Neck Cancer. N Engl J Med. 2016;375(5):491-2.
84. Jones TM, De M, Foran B, Harrington K, Mortimore S. Laryngeal cancer: United Kingdom National Multidisciplinary guidelines. J Laryngol Otol. 2016;130(S2):S75-S82.
85. Gupta T, Master Z, Kannan S, Agarwal JP, Ghsoh-Laskar S, Rangarajan V, et al. Diagnostic performance of post-treatment FDG PET or FDG PET/CT imaging in head and neck cancer: a systematic review and meta-analysis. Eur J Nucl Med Mol Imaging. 2011;38(11):2083-95.
86. Huang SH, O'Sullivan B, Xu W, Zhao H, Chen DD, Ringash J, et al. Temporal nodal regression and regional control after primary radiation therapy for N2-N3 head-and-neck cancer stratified by HPV status. Int J Radiat Oncol Biol Phys. 2013;87(5):1078-85.
87. Marcus C, Ciarallo A, Tahari AK, Mena E, Koch W, Wahl RL, et al. Head and neck PET/CT: therapy response interpretation criteria (Hopkins Criteria)-interreader reliability, accuracy, and survival outcomes. J Nucl Med. 2014;55(9):1411-6.
88. Liu HY, Milne R, Lock G, Panizza BJ, Bernard A, Foote M, et al. Utility of a repeat PET/CT scan in HPV-associated Oropharyngeal Cancer following incomplete nodal response from (chemo)radiotherapy. Oral Oncol. 2019;88:153-9.
89. Slevin F, Subesinghe M, Ramasamy S, Sen M, Scarsbrook AF, Prestwich RJ. Assessment of outcomes with delayed (18)F-FDG PET-CT response assessment in head and neck squamous cell carcinoma. Br J Radiol. 2015;88(1052):20140592.
90. Arunsingh M, Vaidyanathan S, Dyker KE, Sen M, Scarsbrook AF, Prestwich RJD. Accuracy of Response Assessment Positron Emission Tomography-Computed Tomography Following Definitive Radiotherapy Without Chemotherapy for Head and Neck Squamous Cell Carcinoma. Clin Oncol (R Coll Radiol). 2019;31(4):212-8.
91. Meerwein CM, Queiroz M, Kollias S, Hullner M, Veit-Haibach P, Huber GF. Post-treatment surveillance of head and neck cancer: pitfalls in the interpretation of FDG PET-CT/MRI. Swiss Med Wkly. 2015;145:w14116.
92. Wang K, Wong TZ, Amdur RJ, Mendenhall WM, Sheets NC, Green R, et al. Pitfalls of post-treatment PET after de-intensified chemoradiotherapy for HPV-associated oropharynx cancer: Secondary analysis of a phase 2 trial. Oral Oncol. 2018;78:108-13.
93. Liu Q, Ghosh P, Magpayo N, Testa M, Tang S, Gheorghiu L, et al. Lung cancer cell line screen links fanconi anemia/BRCA pathway defects to increased relative biological effectiveness of proton radiation. Int J Radiat Oncol Biol Phys. 2015;91(5):1081-9.

94. Bird D, Scarsbrook AF, Sykes J, Ramasamy S, Subesinghe M, Carey B, et al. Multimodality imaging with CT, MR and FDG-PET for radiotherapy target volume delineation in oropharyngeal squamous cell carcinoma. BMC Cancer. 2015;15:844.

A1: NTCP Models

Radiotherapy is planned to maximise doses to tumour while minimising those to surrounding normal tissues/organs at risk (OARs). The risk of toxicity increases with increasing dose and volume of normal tissue irradiated. The dose-volume parameters that are important vary for the different OARs. For some the planned mean dose to the normal tissue is important and for others it is a particular volume (e.g. V20=the percentage of the volume of an OAR that receives a dose of 20 Gy or more). Normal tissue complication probability (NTCP) models have been developed for different OARs and contain various dose-volume parameters or combinations. The models can also incorporate patient factors when shown to increase risk of toxicity, e.g., patient age, use of concurrent chemotherapy. There are numerous NTCP models and they can also be expressed as nomograms. Validation of models in multiple cohorts has been poor until very recently. Validation involves analysis in retrospective cohorts. Prospective validation within a clinical trial has not been reported.

NTCP models can identify patients who are expected to benefit from IMPT *vs* IMRT (30). The approach involves comparing the dose distributions for IMPT and IMRT using *in silico* comparative planning. Following planning, the magnitude of NTCP sparing is assessed to determine whether a reduction in dose will translate into a lower NTCP. Comparative planning studies showed the estimated reduced NTCP for protons versus photons varies between patients despite similar tumour characteristics. It was shown that NTCP reduction for IMPT *vs* IMRT varied between patients from 4% to 18% for xerostomia. A threshold is then set for the percentage of patients likely to benefit from IMPT. For the xerostomia example and applying a threshold of a 10% reduction in NTCP, 70% of patients would be selected for IMPT.

Dutch Delta-NTCP Thresholds for Patient Selection

Variable	Endpoint		
	**Patient-reported moderate-to-severe xerostomia (EORTC QLQ-HN35; q41)**	**Physician-rated dysphagia (cannot eat solid food or worse)**	**Tube feeding dependence**
**Assessment point**	At 6 months after RT	At 6 months after RT	At 6 months after RT
**Grading**	Grade ≥ II	Grade ≥ II	Grade ≥ III
**ΔNTCP-thresholds**	≥ 10%	≥ 10%	≥ 5%

Patients qualify for proton therapy in case:

- ΔNTCP of at least one Grade ≥ II model ≥ 10%
- ΔNTCP of at least one Grade ≥ III model ≥ 5%
- Sum of the two Grade ≥ II models ≥ 15%

The TORPEdO trial will enable validation of the NTCP models (three endpoints, SUM-NTCP: NTCP xerostomia grade II or higher, NTCP dysphagia grade II or higher, NTCP tube feeding dependence), to assess predicted versus observed toxicity. The models select patients based on the predicted NTCP-profile 6 months after completion of treatment and we shall additionally assess how this relates to an observed increase in UW-QoL physical composite score 12 months after treatment.

The six physical symptoms comprising the UoW physical subscale were related to the EORTC QLQ-HN35 questionnaire to comprise a UW-QoL-equivalent scale. The mean value for SUM-NTCP at 6 months with

IMR T was 111% (range: 64-219%); SD: 30.9%. For a linear regression analysis using the UW-QoL-equivalent score at 12 months as an independent variable and SUM-NTCP as a dependent variable (regression formula: SUMNTCP = 173 - 0.8 * UW-QoL), a UW-QoL-equivalent score of 70 corresponded with a SUM-NTCP of 117, and UW-QoL-equivalent score of 80 corresponded with a SUM NTCP of 109. Using the formula, a mean delta-SUM-NTCP (IMRT vs IMPT) of 15% at 6 months corresponded with an estimated mean delta-UW-QoL score of 18 points at 12 months.

We would therefore additionally hypothesise that for an enriched group of patients with a predicted delta-SUM-NTCP (IMRT vs IMPT) of 15% at 6 months, there would be a 'large' observed increase in UW-QoL physical composite scale at 12 months of ≥ 15 points. While the expected number of patients in the enriched group (approximately 50%) would be sufficient to demonstrate this difference and allow for estimates of precision, it is not possible to sufficiently power a formal test of interaction between the subgroups.

Calculation of NTCP-values for proton and photon plans (66-69)

NTCP patient-rated moderate-to-severe xerostomia = $(1 + e^{-S})^{-1}$

In case baseline xerostomia (EORTC QLQ-HN35 Q41) = '<u>not at all</u>':

$S = - 1.507 + (0.052 * D_{\text{mean contralateral parotid gland}})$

In case baseline xerostomia (EORTC QLQ-HN35 Q41) = '<u>a bit</u>':

$S = - 1.507 + (0.052 * D_{\text{mean contralateral parotid gland}}) + 0.525$

In case baseline xerostomia (EORTC QLQ-HN35 Q41) = '<u>quite a bit to very much</u>':

$S = - 1.507 + (0.052 * D_{\text{mean contralateral parotid gland}}) + 1.482$

NTCP grade ≥ II dysphagia = $(1 + e^{-S})^{-1}$

$S_{\text{dysphagia}} = -3.303 +$
$(0.024 * D_{\text{mean oral cavity}}) +$
$(0.024 * D_{\text{mean superior-PCM}}) +$
$(0.967 * \text{dysphagia grade 2-3})$

$NTCP = (1 + e^{-S})^{-1}$

$S_{\text{feeding tube}} = -6.849 +$
$(0.030 * D_{\text{mean superior PCM}}) +$
$(0.013 * D_{\text{mean inferior PCM}}) +$
$(0.022 * D_{\text{mean contralateral parotid}}) +$
$(0.008 * D_{\text{mean cricpharyngeus}}) +$
$(0.680 * \text{T3-T4}) +$
$(0.198 * \text{accelerated radiotherapy}) +$
$(1.101 * \text{chemoradiation}) +$
$(1.716 * \text{bioradiation}) +$
$(0.317 * \text{weight loss 1-10\%}) +$
$(1.178 * \text{weight loss >10\%})$

Multivariable NTCP model can be combined to a single multivariate logistic NTCP model

$NTCP = (1 + e^{-S})^{-1}$

$S = \beta0 + \sum \beta i \,.\, xi$

With n prognostic variables, x (dosimetric and clinical factors) and regression coefficients β. The above equation shows how multiple models are combined for different endpoints into a single NTCP model.

Secondary OARs planning priorities for treatment optimisation (after contralateral parotid and submandibular glands)

Variable	Endpoint		
	Patient-reported moderate-to-severe xerostomia (EORTC QLQ-HN35; Q41)	Physician-rated dysphagia (cannot eat solid food or worse)	Tube feeding dependence
D_{mean} oral cavity	0.024		
D_{mean} superior PCM	0.024	0.030	
D_{mean} superior PCM		0.022	0.052
D_{mean} PCM inferior		0.013	
D_{mean} cricopharyngeus muscle		0.008	

A2: Health Economics

A within-trial economic component will be integrated into the study to estimate the cost-effectiveness of IMPT from the perspective of the UK NHS, patient and society. Healthcare resource use will be collected from hospital electronic patient records as well as via patient or family member self-report using a participant resource-use questionnaire developed specifically for the study (which will be developed during the course of the trial and submitted as a substantial amendment. The development of the participant resource-use questionnaire will be informed by PPI representatives included within the study research team to capture wider costs to the patient and family of centrally-delivered radiotherapy services (i.e. travel costs, informal carer support and loss of earnings). Patient resource use from the date of randomisation to the end of 1-year follow-up will be used to define cumulative healthcare costs for the within-trial economic component. Resource consequences will include diagnostic and therapeutic procedures and interventions, medications as well as primary care and hospital clinic attendances and inpatient episodes. Examples of resource consequences will include, but will not be limited to, radiotherapy planning and delivery, surgery for node dissection and salvage, dental assessments, imaging modalities and care for acute toxicity side-effects, such as oral dryness, dysphagia and pain management. Hospital length of stay distributions will be valued using a per diem unit cost derived from NHS reference costs. Monetary values will be attached to the labour costs for healthcare practitioners associated with each radiotherapy pathway using standard NHS pay and price estimates. No discounting of costs and outcomes will be undertaken for the within-trial economic component.

The economic component will consider the joint distribution of cumulative hospital costs and quality-adjusted survival on the basis of intention to treat. The EuroQoL EQ-5D-5L utility score will be used to adjust patient survival times in order to calculate quality-adjusted life years. Generalized linear and flexible parametric regression models will be used to estimate the costs and quality-adjusted survival differences between the two treatment groups. The primary outcome for the economic component will be the incremental cost-effectiveness ratio comparing the differing use of health care services and quality-adjusted survival for IMPT versus IMRT.

A3: WHO Performance Status

Grade	*Performance Status*
0	*Able to carry out all normal activity without restriction.*
1	*Restricted in physically strenuous activity but ambulatory and able to carry out light work.*
2	*Ambulatory and capable of all self-care but unable to carry out any work; up and about more than 50% of waking hours.*
3	*Capable of only limited self-care; confined to bed or chair more than 50% of waking hours.*
4	*Completely disabled; cannot carry on any self-care; totally confined to bed or chair.*

A4: Patient Quality of Life and Swallowing Function Assessments

EORTC QLQ-C30 and QLQ-H&N43.

The EORTC QLQ-C30 and associated head and neck module HN43 will be used for assessment of quality of life. Patients will complete the EORTC QLQ C30 general cancer questionnaire (30 questions) which is a brief standardised instrument that provides a simple descriptive profile of their health status (46). In addition they will complete The QLQ-H&N43 is a 43-item questionnaire, with 12 multi-item scales and 7 single-item symptom scales to be used in head and neck patients (47-49).

MD Anderson Dysphagia Inventory (MDADI)

The MDADI is a patient-reported swallowing outcome measure, specifically designed for use with the HNC population. This 20-item written questionnaire has proven reliability and validity, and is sensitive to changes over time. MDADI scores also follow the expected pattern of recovery after organ preservation regimens for HNC, and differentiate between distinct cancer treatment regimens (50, 70, 71). Each item on the MDADI has a five-point response scale. Five scores can be calculated from the MDADI. These include 2 summary scores (global, total/composite) and 3 subscales (emotional, functional, physical) each calculated as a weighted average with a range of 20 (worst impairment) to 100 (no impairment).

The MDADI has been adopted as an outcome measure in international and UK trials (UK: PATHOS (72), DARS, TUBE and De-ESCALaTE (73); US: RTOG-1221 and ECOG-3311). It has also been widely used as a clinical outcome measure in the UK. It is acceptable to patients, quick to complete and easily analysed.

University of Washington Quality of Life Questionnaire (UW-QOL) version 4

The UW-QOL is a validated quality of life tool examining 12 quality of life domains pain, appearance, activity, recreation, swallowing, chewing, speech, shoulder function, taste, saliva, mood and anxiety (13, 44, 45). Each question is scaled from 0 (worst) to 100 (best) according to the hierarchy of response. It also has two global questions about their health-related and overall quality of life, including pain, appearance, activity, recreation, swallowing, chewing and speech. In addition, it is possible to calculate a composite score of physical and social-emotional functioning (13, 44, 45). The UW-QOL questionnaire is a self-administered scale and each of the categories has several options within it that allow the patient to describe their current functional status. Patients also have the opportunity to select up to 3 options from the 12 domains that are most important to them. The whole questionnaire relates to the previous 7 days.

100mL Water Swallow Test (WST)

The 100mL WST forms part of the clinical assessment component. The WST does not require any imaging techniques or invasive procedures. The WST has been shown to have good reliability and validity, is sensitive to measure changes over time and follows the expected pattern in differentiating between different cancer treatment regimes (51, 71, 74, 75). The 100mL WST is an inexpensive and quick method of monitoring swallowing performance over time and is easy to analyse. In a healthy sample, the WST takes no longer than 10 seconds to complete. In a large sample of head and neck cancer patients, it can take up to three minutes to complete. The WST is suitable for use with large patient cohorts, over multiple time points. The WST provides an indication of overall swallowing performance, but unlike videofluoroscopy, it does not provide any information regarding swallow pathophysiology. Therefore we have selected this measure to provide interim information about changes to swallowing function over time, as an adjunct to the more expensive videofluoroscopy assessment.

Performance Status Scales (PSS-HN)

The PSS-HN is a 3-item scale designed to evaluate functional performance of cancer patients, specifically:
1) Normalcy of Diet;
2) Eating in Public and;
3) Understandability of Speech.

Each subscale is rated based on unstructured interview with a score of 0 to 100, with higher scores indicating better performance. The subscale scores are reported separately, and the Normalcy of Diet subscale will be the primary score of interest for this trial.

The PSS-HN has been shown to discriminate levels of functioning across HNCs, and has demonstrated good inter-rater reliability as well as sensitivity to differences in performance and change over time (53, 76, 77). It is quick (taking approximately 5 minutes to complete), inexpensive to administer and does not require a license. The PSS-HN has been incorporated into the UK Dataset for Head and Neck Oncology (DAHNO) and is a National Comprehensive Cancer Network (NCCN) recommended measure in the US. It also allows for the assessor to note enteral tube feeding status alongside these scores.

Functional measures and endpoints

Timepoints	Study	Domain	Endpoint
Baseline, end of treatment, and post treatment: 6 weeks, and 3, 6, 12, 18, 24, 36, 48 and 60 months	MDADI (50)	Swallowing-related QOL (PRO)	Total/Composite, Global, Subscale Scores (continuous scores: 20 to 100)
Baseline, and post treatment: 3, 6, 12, 18 and 24 months	WST (51)	Swallow performance	Swallow capacity (mL/ time)), Swallow volume (mL per swallow)
Baseline, and post treatment: 3, 6, 12, 18 and 24 months	PSS-HN (52, 53)	Functional performance status	Diet, Eating in Public, Understandability of Speech Scores (ordinal: 0 to 100)
Baseline, end of treatment, and post treatment: 6 weeks, and 3, 6, 12, 18, 24, 36, 48 and 60 months	UW-QoL v4.0 (13, 44, 45)	Health related quality of life	Each question is scaled from 0 (worst) to 100 (best) according to the hierarchy of response. Can calculate a composite score of physical and social-emotional functioning. Patient can select up to 3 options from the 12 domains that are most important to them.

A5: Evaluation of Response

Response assessment with PET-CT

Response assessment with 2-[Fluorine-18]-fluoro-2-deoxy-D-glucose (FDG) positron emission tomography-computed tomography (PET-CT has a well-established role following chemoradiotherapy with a high negative predictive value (NPV) at primary site and lymph nodes (78-82). This applies even in the context of metabolically inactive residual masses (78, 83).

In the randomised PET-NECK trial in node positive patients, a strategy of FDG PET-CT guided surveillance had non-inferior survival compared with a planned neck dissection and was found to be more cost effective (65). Response assessment with FDG PET-CT following chemoradiotherapy is now embedded in PET-CT imaging guidelines published in 2016 by the Royal College of Radiologists and Head and Neck Cancer: UK National Multidisciplinary Guidelines in 2016 (4, 84). In a meta-analysis, PET scans at least 12 weeks post-treatment had a higher sensitivity than <12 weeks (85). It has been recognised that lymph nodes in HPV-positive disease may continue to regress longer than in HPV-negative disease (86). A complete metabolic response in the presence of residual enlarged nodes has a high NPV in human papilloma virus (HPV)-positive oropharyngeal carcinoma (82), leading to a recommendation for imaging follow-up in this patient sub-group (65).

There is no consensus regarding the optimal interpretive criteria for PET-CT response assessment in head and neck squamous cell carcinoma. Semi-quantitative methods of treatment response assessment using standardised uptake value (SUV) have not been shown to be accurate at predicting patient outcome which has led to the development of more reproducible qualitative interpretation criteria to assess post-treatment response (78, 87). One widely used method reported by Porceddu et al. and used by others (78, 88-90), classifies scans as positive, negative or equivocal based on whether there is FDG activity greater than adjacent normal tissues and/or liver. Areas of FDG uptake were classified as positive if uptake was focal, corresponding to a structural abnormality and of greater intensity than background liver activity. Scans were classed as equivocal if focal FDG uptake was reduced from baseline, was below liver background but above that of surrounding normal tissues. Scans were classed as negative in the absence of any abnormal focal FDG uptake or diffuse FDG uptake in the absence of corresponding anatomical abnormality on the CT which was considered to be radiotherapy-related.

Whilst high NPVs provide the basis for clinical utility, PET-CT scans are susceptible to false positive results, due to FDG avidity in the context of post-radiotherapy inflammation, reactive changes in lymph node tissue and physiological uptake (91). This is reflected in the consistent reporting of limited positive predictive values (PPV) (80-82, 88, 89, 92). In a meta-analysis a PPV of 52% was reported(85), whilst individual series have shown much lower PPVs. For example, in one report the PPV of equivocal nodal uptake was only 20% (89). This suggests that a policy of intervention for all patients who do not achieve a complete response on PET-CT represents overtreatment (92). Additionally the PPV is lower for HPV-related oropharyngeal carcinoma with a more favourable prognosis and a lower pre-treatment probability of residual disease (88). A secondary analysis of a phase II trial in HPV oropharyngeal carcinoma confirmed that an incomplete or equivocal response assessment PET-CT is poor at predicting persistent disease, and supports an approach of surveillance rather than surgical intervention in patients with favourable risk oropharyngeal carcinoma (92).

In view of the low PPV of response assessment PET-CT, several groups (80, 82, 88, 89) have adopted a strategy of a second interval PET-CT response assessment aiming to discriminate between a slow response to therapy/false positive uptake and persistent disease. Liu et al (93) reported on a series of 41 patients with HPV-associated oropharyngeal carcinoma and an equivocal or partial nodal response on initial PET-CT 12 weeks post chemoradiotherapy. A repeat PET-CT was performed after a further 16 weeks. There was a 71% conversion rate to a complete response and a high NPV of 97%; PPV remained low at 33%. The authors recommended the use of repeat PET-CT to spare patients from unnecessary neck dissection. Vainshtein et al (82) also reported a series of patients with HPV-related oropharyngeal carcinoma and undertook routine serial surveillance. As part of this programme, 21/22 (95%) and 12/12 (100%) of patients with an incomplete response at the primary site and nodes respectively on initial 3-month PET-CT assessment converted to a complete response on subsequent surveillance PET-CT. Bird et al. (94) have also suggested that a repeat PET-CT is appropriate following an equivocal response, based upon a high rate of reassuring PET-CT findings on repeat PET-CT whilst allowing successful salvage surgery in the remainder. In unpublished data (personal communication) from Leeds in a series comprising predominantly patients with HPV-related oropharyngeal carcinoma a repeat PET-CT scan was performed at a median interval of 12 weeks following an equivocal or positive initial PET-CT response assessment provided a 60% conversion rate to complete metabolic response whilst allowing successful salvage of patients with persistent abnormality (although PPV remained low).

Recommendations for HPV-related oropharyngeal disease (for guidance only, local practice should be followed)

1. PET-CT to be performed 12-14 weeks after completion of chemo-radiotherapy.
2. Ideally PET-CT response assessment reporting is performed in context of baseline PET-CT to ensure disease was FDG-avid (e.g., may not be the case with necrotic nodal disease).
3. PET-CT response assessment must be correlated with clinical assessment
4. Patients with a complete metabolic response and a residual mass, in the presence of reassuring clinical examination/assessment, can be managed depending on local MDT decision with an interval scan (PETCT, MRI or CT with contrast) after 8-12 weeks, or clinical observation.
5. Patients with a complete metabolic response and a residual mass, in the presence of concerning clinical examination/assessment, EUA and biopsy of primary site or neck dissection should be considered.
6. Patients with incomplete metabolic PET-CT response and concerning clinical examination/assessment, EUA and biopsy of primary site or neck dissection should be considered.
7. Patients with an equivocal PET-CT response assessment in nodes or primary site and a reassuring clinical examination/assessment, should be managed with an interval repeat PET-CT after 8-12 weeks.
8. Patients with a positive PET-CT response assessment in nodes or primary site, and a reassuring clinical examination/assessment can be managed based on clinical discretion with an interval repeat PET-CT after 8-12 weeks, or EUA and biopsy of primary site or a neck dissection.
9. If incomplete metabolic response on repeat PET-CT, a neck dissection/EUA and biopsy of primary site should be considered depending upon scenario of concern regarding nodal and/or primary tumour response.

Recommendations for HPV-negative oropharyngeal disease (for guidance only, local practice should be followed)

1. PET-CT to be performed 12-14 weeks after completion of chemo-radiotherapy
2. Ideally PET-CT response assessment reporting is performed in context of baseline PET-CT to ensure disease was FDG-avid (e.g., may not be the case with necrotic nodal disease).
3. PET-CT response assessment must be correlated with clinical assessment
4. Patients with a complete metabolic response but presence of a residual mass, equivocal PET-CT response or positive PET-CT response, EUA and biopsy of primary site or neck dissection should be considered.

Summary recommendations (for guidance only, local practice should be followed)

**PET-CT**<br>**12-14 weeks post chemo-radiotherapy**	**Interpretation**	**Action**
Focal FDG uptake<br>with greater intensity than background liver activity<br>and corresponded to a structural abnormality	POSITIVE	**HPV-related**<br>If reassuring clinical examination/assessment, managed based on local MDT decision with an interval repeat PET-CT after 8-12 weeks, or EUA and biopsy of primary site or a neck dissection.<br>If concerning clinical examination/assessment, EUA and biopsy of primary site or a neck dissection<br><br>**HPV-negative**<br>EUA and biopsy of primary site or neck dissection. (78)
Focal FDG uptake below background liver activity<br>but<br>greater intensity than adjacent normal tissue activity and<br>corresponded to a structural abnormality	EQUIVOCAL	**HPV-related**<br>If reassuring clinical examination/assessment, interval repeat PET-CT after 8-12 weeks.<br>If concerning clinical examination/assessment, EUA and biopsy of primary site or neck dissection<br><br>**HPV-negative**<br>EUA and biopsy of primary site or neck dissection
No residual FDG avidity above adjacent normal tissue activity but persistent structural abnormality at primary site or neck node ≥ 10 mm	NEGATIVE – complete metabolic response, residual mass	**HPV-related**<br>If reassuring clinical examination/assessment, managed based on local MDT decision with an interval scan (MRI or CT with contrast) after 8-12 weeks, or clinical observation<br>If concerning clinical examination/assessment, EUA and biopsy of primary site or neck dissection<br><br>**HPV-negative**<br>If reassuring clinical examination/assessment,

		managed based on local MDT decision with an interval scan (MRI or CT with contrast) after 8-12 weeks<br>or EUA and biopsy of primary site or neck dissection
No residual FDG avidity above adjacent normal tissue activity or diffuse FDG uptake on PET imaging without corresponding suspicious structural abnormality (including lymph node < 10 mm)	NEGATIVE – complete metabolic response, no residual mass	**HPV-related**<br>Clinical observation<br><br>**HPV-negative**<br>Clinical observation
