# Supplementary material for: TORPEdO: A phase III trial of intensity-modulated proton beam therapy versus intensity-modulated radiotherapy for multi-toxicity reduction in oropharyngeal cancer
Source: Clin Transl Radiat Oncol. 2022 Nov 21;38:147–54. doi: 10.1016/j.ctro.2022.11.010 (PMC9702982; doi:10.1016/j.ctro.2022.11.010)
Supplement: Supplementary data 2 [file mmc2.pdf]

A phase III trial of intensity-modulated proton beam therapy versus intensity-modulated radiotherapy for multi-toxicity reduction in oropharyngeal cancer.

RADIOTHERAPY TREATMENT PLANNING AND DELIVERY, AND QA GUIDELINES

Version: 3.0
Dated: 9th Feb 2021

Chief Investigator:	Dr David Thomson (The Christie NHS Foundation Trust)
Sponsor:	The Institute of Cancer Research
Approval:	Cancer Research UK: Clinical Research Committee (CRUK CRC)
Funders:	Cancer Research UK
Additional Financial Support:	The Taylor Family Foundation
Coordinating Trials Unit:	ICR Clinical Trials and Statistics Unit (ICR-CTSU)
	The Institute of Cancer Research

Main REC Reference Number: 19/NW/0700

Protocol Number: ICR-CTSU/2019/10067

CCR Number: CCR5134

ISRCTN: 16424014

CRUK Reference Number: CRUK/18/010
IRAS ID: 268843

The TORPEdO trial has been approved by Cancer Research UK's Clinical Research Committee (CRUK CRC).
The TORPEdO trial is part of the National Institute for
Health Research Clinical Research Network Trial Portfolio

This is a controlled document which should be referred to in conjunction with the TORPEdO protocol and should not be copied, distributed or reproduced.

TRIAL ADMINISTRATION

Scientific Coordination

Dr David Thomson (Chief Investigator)
The Christie NHS Foundation Trust
Wilmslow Road
Manchester
M20 4BX

Translational Lead
Prof Catharine West
Professor of Radiation Biology, Translational Radiobiology Group
Division of Cancer Sciences
School of Medical Sciences, Faculty of Biology, Medicine and Health
The University of Manchester, Manchester Academic Health Science Centre
The Christie NHS Foundation Trust
Wilmslow Road
Manchester
M20 4BX

Trial Coordination

ICR-CTSU (a UKCRC registered CTU and an NCRI cancer clinical trials unit) is responsible for the central coordination of the trial on behalf of the Sponsor.

ICR Clinical Trials & Statistics Unit (ICR-CTSU)
Division of Clinical Studies
The Institute of Cancer Research
Sir Richard Doll Building
Cotswold Road
Sutton, Surrey SM2 5NG

ICR-CTSU Scientific/Trial Methodology Lead: Emma Hall
Tel: 020 8722 4013
Email: Emma.Hall@icr.ac.uk

ICR-CTSU Statistician: Abdullahi Omar
Tel: 020 8722 4535
Email: Abdullahi.Omar@icr.ac.uk

ICR-CTSU Clinical Trials Programme Manager: Clare Cruickshank
Tel: 020 8722 4058
Email: Clare.Cruickshank@icr.ac.uk

ICR-CTSU Trial Manager: Vicky Scott
Tel: 020 8722 4157
Email: Vicky.Scott@icr.ac.uk
TORPEdO-icrctsu@icr.ac.uk

Radiotherapy Quality Assurance Team Overview

Primary Contact for General Queries
Dr James Price
Senior Clinical Fellow
Clinical QA Lead for TORPEdO
The Christie NHS Foundation Trust
Email: James.Price@nhs.net

Primary Contact for QA Queries
Julian Phillips, Justine Tyler, Helen Baines
RTTQA Contacts
Email: TORPEdOqa.enh-tr@nhs.net

Clinical Contacts

Dr Nachi Palaniappan<br>Consultant Clinical Oncologist<br>Velindre NHS Trust<br>Email: Nachi.Palaniappan@wales.nhs.uk	Professor Chris Nutting<br>Consultant Clinical Oncologist<br>The Royal Marsden NHS Foundation Trust<br>Email: chris.nutting@rmh.nhs.uk
Dr Robin Prestwich<br>Consultant Clinical Oncologist<br>Leeds Teaching Hospitals NHS Trust<br>Email: robin.prestwich@nhs.net	Dr Bernie Foran<br>Consultant Clinical Oncologist<br>Sheffield Teaching Hospitals NHS Foundation Trust<br>Email: b.foran@nhs.net
Dr Andrew McPartlin<br>Consultant Clinical Oncologist<br>The Christie NHS Foundation Trust<br>Email: Andrew.McPartlin@nhs.net	Dr David Thomson<br>Consultant Clinical Oncologist<br>The Christie NHS Foundation Trust<br>Email: David.Thomson2@nhs.net
Dr Anna Thompson<br>Consultant Clinical Oncologist<br>University College London Hospitals NHS Foundation Trust<br>Email: annathompson1@nhs.net	

Physics Contacts

Dr Mat Lowe<br>Proton Beam Therapy Physicist<br>The Christie NHS Foundation Trust<br>Email: Matthew.Lowe11@nhs.net	Mr Callum Gillies<br>Proton Beam Therapy Physicist<br>University College London Hospitals NHS Foundation Trust<br>Email: callumgillies@nhs.net

National RTTQA Group

Mrs Justine Tyler<br>RTTQA Physicist<br>TORPEdOqa.enh-tr@nhs.net	Ms Helen Baines<br>RTTQA Physicist<br>TORPEdOqa.enh-tr@nhs.net
Mr Julian Phillips<br>RTTQA Dosimetrist<br>TORPEdOqa.enh-tr@nhs.net	
Mrs Elizabeth Miles<br>RTTQA Co-ordinator<br>TORPEdOqa.enh-tr@nhs.net	Dr Chloe Brooks<br>RTTQA Research Fellow<br>TORPEdOqa.enh-tr@nhs.net

FOREWORD

This document describes the radiotherapy outlining, planning and QA processes for the TORPEdO trial. When used in conjunction with the main trial protocol it provides all the information necessary for entering patients into the trial. This document should not be used as a guide for the treatment of patients outside of the trial.

Every care has been taken in drafting these guidelines but corrections or amendments may be necessary. These will be circulated to investigators in the trial, but centres entering patients for the first time are advised to contact the ICR-CTSU (TORPEdO-icrctsu@icr.ac.uk) to ensure they have the most recent and approved version.

If you have any queries in regards to these guidelines please contact the RTTQA group by email on the following address: TORPEdOqa.enh-tr@nhs.net.

DETAILS OF AMENDMENTS TO THE TORPEdO RT GUIDELINES

DOCUMENT VERSION AND DATE	SUMMARY OF CHANGES
Version 1.0 08Nov2019	Original approved version
Version 2.0	Contact details updated
Version 3.0	- RTTQA staff changes<br>- CTV1p and CTV2p expansions updated for patients who have undergone simple (diagnostic) tonsillectomy (page 12)<br>- Clarified need for CTV2 not to overlap with CTV1<br>- OAR descriptions brought in line with GHG consensus guidelines where possible<br>- Upper oesophagus (cervical) caudal border re-defined as suprasternal notch<br>- Minor edits to data collection for proton patients

Contents

1. TRIAL SUMMARY 7

2. TRIAL SCHEMA 9

3. INTRODUCTION 10

4. PATIENT IMMOBILISATION AND IMAGE ACQUISITION 10

5. ANATOMY 11

6. TARGET LOCALISATION AND OUTLINING 13

6.1. Treatment of the neck: 13

6.2. Definition of target volumes 13

6.3. Organs at Risk (OARs) delineation: 17

6.4. Example outlining case (applicable to both IMPT and IMRT) 21

7. TREATMENT PLANNING 24

7.1. IMRT 24

7.2. IMPT 24

8. VERIFICATION AND REPLANNING 27

9. TREATMENT INTERRUPTIONS 28

10. RADIOTHERAPY QUALITY ASSURANCE 29

11. OUTLINING BENCHMARK CASE 31

12. PLANNING BENCHMARK CASE 32

13. REFERENCES 33

1. TRIAL SUMMARY

Protocol title	**TORPEdO - A phase III trial of intensity-modulated proton beam therapy versus intensity-modulated radiotherapy for multi-toxicity reduction in oropharyngeal cancer.**
Short title	TOxicity Reduction using Proton bEam therapy for Oropharyngeal cancer
Trial population	Men and women aged 18 years and over with newly diagnosed oropharyngeal cancer who require concurrent chemo-radiotherapy including bilateral neck treatment.
Target disease	Oropharynx cancer, both HPV positive and HPV-negative disease<br>HPV positive (TNM-8): T1-2 N1-2 (excluding T1 single ipsilateral node <3cm), T3-4 N0-2.<br>HPV negative (TNM8): T1 N2, T2 N1-N2, T3-4 N0-2<br>N3 disease excluded
Study objectives	The primary aim is to assess whether intensity modulated proton therapy (IMPT) compared with intensity modulated radiotherapy treatment (IMRT) reduces toxicity in oropharyngeal squamous cell carcinoma.<br><br>The secondary aim is to validate a biomarker (NTCP-model) as a predictor of benefit from IMPT *vs* IMRT.
Study design	Phase III randomised, controlled, multicentre radiation technology trial.
Recruitment target	183 patients
Trial treatment	Consenting patients will be randomised in a 2:1 ratio to IMPT at The Christie or UCLH vs standard IMRT at the recruiting centre.<br><br>Radiotherapy doses will be same in both treatment groups:<br>● therapeutic dose of 70 Gy (relative biological effective (RBE) equivalent)<br>● elective dose of 56 Gy (RBE equivalent)<br>● delivered in 33 once daily fractions over 6.5 weeks
Chemotherapy	Chemotherapy in the context of this trial is not an investigational medicinal product.<br><br>All patients should be suitable for concurrent platinum chemoradiotherapy. Cisplatin 100 mg/m^2 on day 1 and day 22 of radiotherapy will be mandated for all patients with adequate creatinine clearance (≥60 ml/min calculated by Cockcroft-Gault formula prior to randomisation). If patients subsequently develop renal impairment, ototoxicity or neuropathy, carboplatin AUC=5 may be substituted for the second cycle of chemotherapy.
Co-Primary endpoints	The co-primary endpoints are:<br>● University of Washington Quality of Life Questionnaire (UW-QoL) physical composite score<br>● gastrostomy dependence or CTCAE grade 3 weight loss (i.e. ≥ 20% weight loss from baseline)<br>measured at 12 months after completion of chemoradiotherapy.

Secondary endpoints	● Longitudinal pattern of health related quality of life (HR-QoL).<br>● Tube feeding status.<br>• Acute and late severe toxicity.<br>●Swallowing function.<br>●Performance Status Scale for Head and Neck Cancer (PSS-HN).<br>• Hearing loss.<br>• Trismus.<br>• Resection rates.<br>● Loco-regional tumour control.<br>● Overall survival.<br>● Cost-effectiveness.
Trial assessments and follow up	● **Audiometry and trismus**<br>Audiometry, and trismus by change in maximum interincisal distance, at baseline, and at 3, 12 and 24 months post treatment<br>● **Radiological imaging** (MRI/CT/PET-CT)<br>At baseline, 12-14 weeks after completion of therapy, and subsequently as required by the investigator if clinical review raises the suspicion of recurrent disease.<br>● **Evaluation of swallowing function**<br>100ml water swallow test and Performance Status Scale for Head and Neck Cancer (PSS-HN) at baseline, and at 3, 6, 12, 18 and 24 months following completion of study treatment.<br>● **Quality of life assessment**<br>Patient Reported Outcomes (PRO) booklets containing the validated UW-QoL, EORTC QLQ-C30 and QLQ-H&N43, MDADI, Work Productivity Assessment Index (WPAI-SHP) (only until 24 months), EuroQol five-dimensional questionnaire (EQ-5D-5L) (only until 24 months) and Healthcare resource-use (only until month 12) instruments will be given to the patient in clinic at baseline, at the end of treatment, 6 weeks post-treatment, and then sent to the patient's home address for completion at 3, 6, 12, 18, 24, 36, 48, and 60 months.<br>● **Clinical follow-up is consistent with routine practice**<br>Patients will be assessed weekly during treatment, 6 weeks post-treatment, and at 3, 6, 12, 18 and 24 months post-treatment. For the purposes of the trial, subsequent routine follow-up data will be collected annually in years 3-5.
Imaging and physics-based translational research	(1) Pre-accrual (benchmarking) and during-accrual (recruited patient) DICOM-RT and associated non-DICOM data will be collected to validate and develop improved NTCP models and refine patient selection for IMPT.<br>(2) Image-based data mining of DICOM data is planned to correlate per-voxel radiotherapy doses.
Optional biological sample collection	Archival diagnostic tissue and blood samples will be requested from all consenting patients. Blood samples will be collected at baseline, during and post treatment up to the 3 month (post treatment) timepoint.
Optional MRI imaging sub study	Patients receiving treatment at The Christie Hospital (either IMPT or IMRT) will be asked to have an additional diffusion-weighted (DW)-MRI. Separate patient information will be provided regarding this sub study.

2. TRIAL SCHEMA

Patients >18 years' old with oropharyngeal cancer, either HPV positive or HPV-negative disease,
(HPV positive [TNM-8] T1-2 N1-2 (excluding T1 with a single ipsilateral node <3cm), T3-4 N0-2;
HPV negative [TNM-8] T1N2, T2N1-N2, T3-4N0-2
p16 status ($p16^{InK4A}$ IHC/ISH)
Planned treatment is curative chemo-radiotherapy with bilateral neck treatment

CONSENT

RANDOMISATION 2:1 (IMPT vs standard IMRT)

IMPT: The Christie or UCLH
70Gy(RBE)/56Gy(RBE) in 33# (6.5 weeks)

IMRT: Randomising Centre
70Gy/56Gy in 33# (6.5 weeks)

Baseline assessments

Patient reported outcomes (PRO): University of Washington Quality of Life (UW-QoL), EORTC QLQ-C30 and QLQ-H&N43, M. D. Anderson Dysphagia Inventory (MDADI), health economics (Healthcare resource-use, EQ-5D-5L and Work Productivity Assessment Index (WPAI-SHP)) questionnaires
Audiometry
Baseline signs and symptoms (CTCAE v 5.0)
Weight and WHO Performance Status (WHO PS)
Feeding tube status
Maximum interincisal distance to assess trismus
Evaluation of swallowing dysfunction using the 100mL Water Swallow Test
Diet and eating scores evaluated using the Performance Status Scale for Head and Neck Cancer [illegible]

Follow-up assessments

Weeks 1-6 on treatment
Clinician-reported toxicities (CTCAE v 5.0) & physician-rated dysphagia scores.
Feeding tube status, weight, and WHO PS

End of treatment (week 7) and 6 weeks post treatment
PRO (UW-QoL, EORTC QLQ-C30, QLQ-H&N43, MDADI, EQ-5D-5L, WPAI-SHP)
Clinician-reported toxicities (CTCAE v 5.0)
Feeding tube status, weight, and WHO PS

3, 6, 12, 18 and 24 months post-treatment
PRO (UW-QoL, EORTC QLQ-C30, QLQ-H&N43, MDADI, EQ-5D-5L, WPAI-SHP)
Healthcare resource-use (3 months, 6 months, 12 months)
Clinician-reported toxicity (CTCAE v 5.0 & LENT SOMA)
Feeding tube status, weight, and WHO PS
Audiometry and maximum interincisal distance to assess trismus (3, 12 and 24 months only)
100mL Water Swallow Test, Diet and Eating scores evaluated using the PSS-HN.
Clinical response assessment
Radiological assessment at 3 months

3, 4 and 5 years post-treatment
PRO (UW-QoL, EORTC QLQ-C30, QLQ-H&N43, MDADI, EQ-5D)
Assessment for recurrence and survival only.

Optional Biological Sample Collection: Archival diagnostic tissue and blood samples will be requested from all consenting patients. Blood samples will be collected at baseline, during and post treatment up to the 3-month (post treatment) timepoint.

Optional MRI imaging sub study: Patients receiving treatment at the Christie Hospital (either IMPT or IMRT) will be asked to have an additional diffusion-weighted (DW) MRI at baseline and then at week 3 during their treatment.

3. INTRODUCTION

Patients entered in the TORPEdO trial are randomised to receive either intensity modulated proton therapy (IMPT) at The Christie (Manchester) or UCLH (London), or intensity modulated radiotherapy (IMRT) at their local radiotherapy centre.

Radiotherapy treatment should commence as soon as possible and ideally within 4 weeks after randomisation.

For IMPT and IMRT, treatment is via a simultaneous integrated boost in a single phase, as follows:

- 70Gy(RBE) in 33 fractions (2.12 Gy(RBE) per fraction) over 6.5 weeks to the therapeutic clinical target volume (CTV1) for IMPT, and the therapeutic planning target volume (PTV1) for IMRT.
- 56Gy(RBE) in 33 fractions (1.70Gy(RBE) per fraction) over 6.5 weeks to areas at risk of microscopic disease (CTV2 for IMPT, and PTV2 for IMRT).

In this document the RBE weighted dose in units of Gy(RBE) is used to describe the product of the absorbed dose and the RBE. For protons, the RBE should be interpreted as 1.1. For photons this should be interpreted as 1 for which Gy(RBE) is equivalent to the absorbed dose in Gy.

Radiotherapy planning and outlining should be carried out in accordance with the guidelines in the current version of the TORPEdO Radiotherapy Treatment Planning and Delivery and QA guidelines available on request from ICR-CTSU (TORPEdO-icrctsu@icr.ac.uk) or found on the Radiotherapy Trials Quality Assurance (RTTQA) website www.rttrialsqa.org.uk.

4. PATIENT IMMOBILISATION AND IMAGE ACQUISITION

All patients should be immobilised in a supine position using a 5-point customised thermoplastic shell.

Patients should undergo a radiotherapy planning CT scan in the immobilisation shell, with a maximum CT slice thickness of 3mm, from the vertex of the skull superiorly to the carina inferiorly.

For both IMPT and IMRT planning scans, the use of intravenous contrast is recommended in order to facilitate optimal delineation. For IMPT, an additional pre-contrast scan should be acquired and rigidly co-registered with the post-contrast scan.

An MRI scan of the neck, either diagnostic or in the treatment position, is also recommended to aid delineation of the target volumes and organs at risk. Rigid or deformable registration of the MRI scan with the planning CT may be helpful, depending on a clinical assessment of the quality of co-registration.

5. ANATOMY

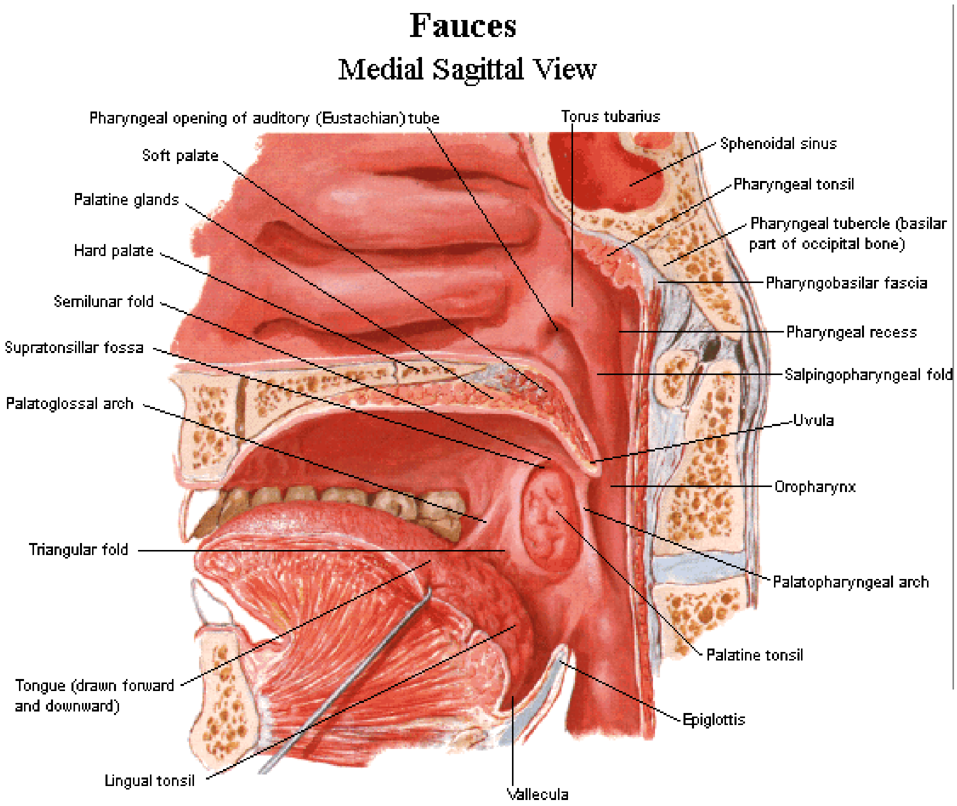

Figure 1 – anatomy of the oropharynx, sagittal view[1]

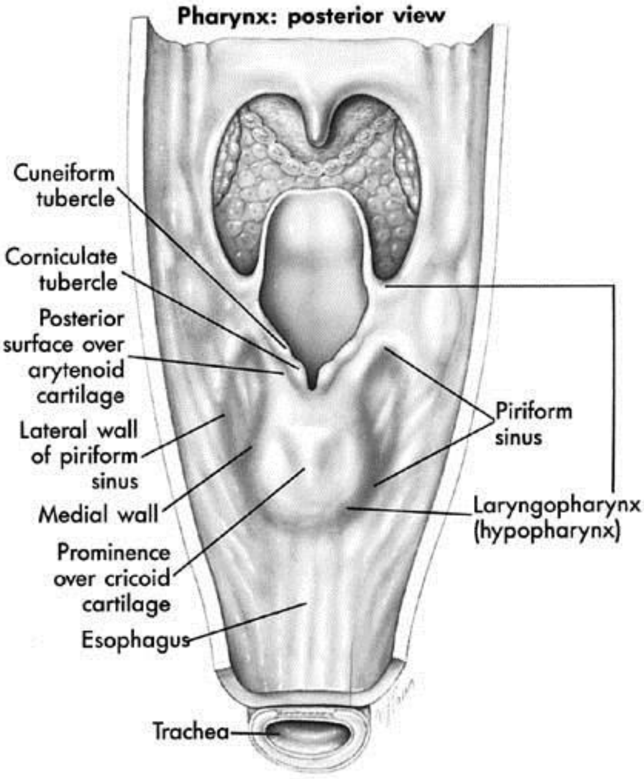

Figure 2 – anatomy of the pharynx, posterior view[2]

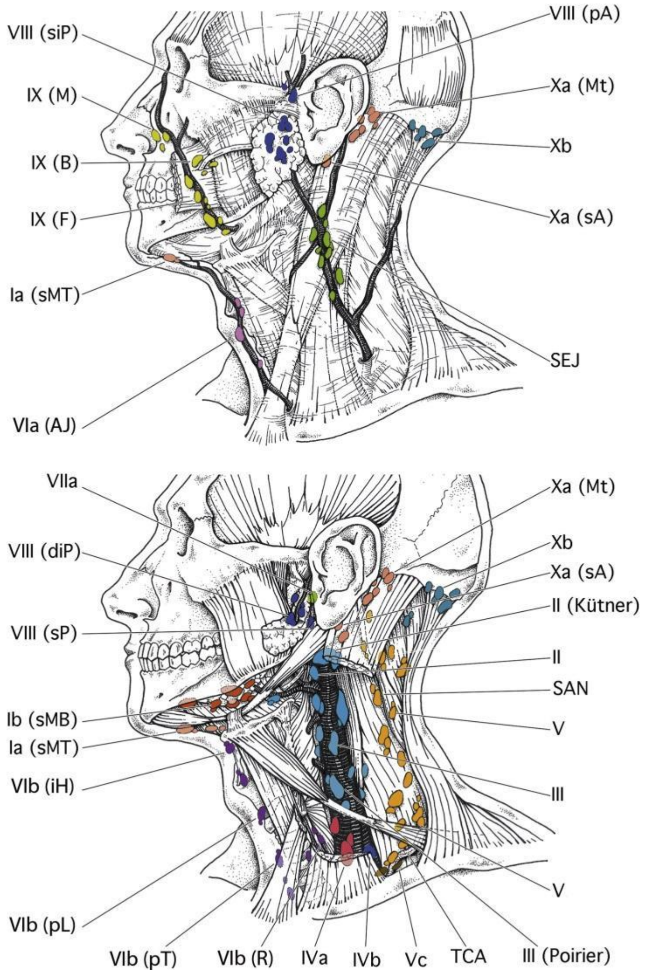

Figure 3 – superficial and deep lymphatic nodal groups of the head and neck[3]

6. TARGET LOCALISATION AND OUTLINING

6.1. Treatment of the neck:

- The TORPEdO trial requires that all patients undergo bilateral neck irradiation, i.e., those patients with non-lateralised tumours, bilaterally involved lymph nodes, **or** lateralised tumours with multiple ipsilateral nodes.
- Lateralised tumours are defined as
    - tumours of the tonsillar fossa / lateral pharyngeal wall with <1cm medial disease extension AND >1cm clearance from the midline, **and**
    - Without any soft palate, base of tongue or posterior pharyngeal wall involvement.

6.2. Definition of target volumes

- The *primary tumour clinical target volumes* are defined according to the 2017 consensus guidelines by Gregoire et al[4], using a "5mm + 5mm" expansion of the primary tumour gross tumour volume (GTVp) to create CTV1p and CTV2p respectively.
- Following geometric expansion, contours should be edited for air cavities, and for anatomic boundaries to tumour spread (e.g. bone and fascia). In some circumstances, CTV2p contours should be edited off adjacent anatomical structures dependent on the oropharyngeal sub-site and T-stage as described in the consensus guidelines[4], and outlined in Table 1.
- A 10mm expansion to create CTV1p may be used in place of the "5mm + 5mm" approach at the discretion of the treating oncologist in the following instances:
    - For indistinct primary tumours
    - Where a patient has undergone simple tonsillectomy and the pre-tonsillectomy GTVp was not apparent on diagnostic imaging, the tonsillar bed should be outlined and a 10mm expansion applied to form CTV1p. However, if the pre-tonsillectomy diagnostic imaging demonstrates the GTVp, the pre-tonsillectomy GTVp should be recreated and standard expansions applied to form CTV1p and CTV2p.
- The *nodal clinical target volume* (CTV1n) is defined by a 10mm isotropic expansion from the nodal GTV(s).
- At-risk lymph node levels (CTV2n) are defined according to Table 2 (below) and will receive an elective dose of 56Gy(RBE).
- The delineation of lymph node levels for elective radiation is defined by the 2013 consensus guidelines by Gregoire et al[3]. An illustrative atlas is available online (https://www.rtog.org/LinkClick.aspx?fileticket=uQmTaI3efxE%3d&tabid=229)
- CTV1 and CTV2 will receive 70 Gy(RBE) and 56 Gy(RBE) respectively, in 33 once daily fractions of 2.12 Gy(RBE) and 1.70 Gy(RBE) respectively, Monday-Friday over 6.5 weeks.
- Trial nomenclature for target volumes is given in Table 3.

Table 1: Summarising where, following expansion from GTVp and after editing for air and barriers to spread, CTV2p should be edited off adjacent anatomical structures:

	**T1 tumours**	**T2 tumours**	**T3 tumours**	**T4 tumours**
**Tonsil**	Edit CTV2p off parapharyngeal space	Edit CTV2p off medial pterygoid muscle		
**Soft palate**		Edit CTV2p off medial pterygoid muscle and mobile tongue	Edit CTV2p off mobile tongue	
**Posterior pharyngeal wall**		Edit CTV2p off pre-vertebral fascia	Edit CTV2p off pre-vertebral fascia	
**Vallecular**	Edit CTV2p off pre-epiglottic space			
**Base of tongue**	Edit CTV2p off hyo-glossus muscle			

Table 2: Selection of Nodal Levels for Elective Radiotherapy:

**Node-positive neck**[#]	***Ipsilateral*** **node-negative neck**	***Contralateral*** **node-negative neck**
Levels Ib, II, III, IVa, Va, Vb, VIIa[†], VIIb[‡]<br><br>*[#]where level IVa or Vb is involved, include levels IVb and Vc (i.e. the medial and lateral supraclavicular fossa)*<br><br>*[†]we recommend that the cranial border of level VIIa is defined as the upper edge of the body of C1 or the upper extent of the hard palate, whichever is more cranial*<br><br>*[‡]where level II is involved*	Levels II, III, IVa, VIIa	Levels II[§], III, IVa, (VIIa[-])<br><br>*[§]for the contralateral node-negative neck only, the cranial border of level II is defined as where the internal jugular vein crosses the posterior belly of the jugulodigastric muscle[5] – see figure 4*<br><br>*[-]If the primary tumour involves the posterior pharyngeal wall or arises from / extends >1 cm on to the soft palate* ***or*** *if there is ipsilateral VII involvement*

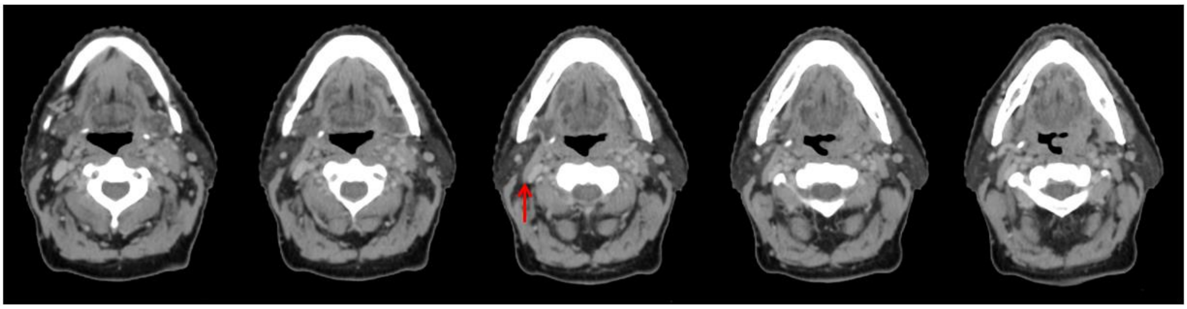

Figure 4 – series of axial CT slices illustrating where the internal jugular vein crosses the posterior belly of the digastric muscle (red arrow). This slice represents the cranial extent of CTV2n for the contralateral node negative neck. Left to right: inferior to superior.

Table 3. Trial nomenclature for target volumes

**Volume**	**Trial nomenclature**
GTV primary	GTVp
GTV involved nodes	GTVn
CTV high risk primary	CTV1p
CTV high risk nodal	CTV1n
Clinical target volume high risk	CTV1
CTV primary elective	CTV2p
CTV nodal elective	CTV2n
Clinical target volume elective	CTV2
PTV high risk	PTV1
PTV low risk elective	PTV2
PTV high risk cropped for skin build up and overlap with a margin around critical structure	PlanPTV1
PTV low risk elective cropped for skin build up and overlap with a margin around critical structure	PlanPTV2
CTV high risk cropped for skin build up and overlap with a margin around critical structures	PlanCTV1
CTV low risk elective cropped for skin build up and overlap with a margin around critical structures	PlanCTV2

Step 1 – outline primary tumour and nodal GTVs

Using all available information, e.g. findings of clinical examination, examination under anaesthesia, diagnostic imaging (CT, MRI +/- PET-CT), delineate gross tumour volumes (GTV) for the primary tumour (GTVp) and involved nodes (GTVn).

Step 2 – create CTV1

Add a 5mm isotropic margin to GTVp to create CTV1p (see text above for instances where a 10mm expansion may be used).

Add a 10mm isotropic margin to GTVn to create CTV1n.

Edit for air cavities and anatomical barriers to spread e.g. bone and fascia (including platysma).

Combine CTV1p and CTV1n to create the final CTV1.

Note:

- The entire involved lymph node level is **not** included in CTV1
- In the presence of clinical/radiological extra-nodal extension (cENE), CTV1 should not include the entirety of the sternocleidomastoid muscle.

Step 3 – create CTV2

Add a 10mm isotropic margin to GTVp to create CTV2p.

Edit for air cavities, anatomical barriers to spread (e.g. bone and fascia) and, where appropriate, for adjacent anatomical structures (see Table 1).

Outline CTV2n, including all uninvolved nodal levels that are at risk of microscopic disease (see Table 2).

Combine CTV2p and CTV2n to create the final CTV2.

Subtract CTV1 from CTV2 so ensure no overlap of CTV volumes.

Step 4 – create PTV1 and PTV2

For IMRT, the Planning Target Volumes (PTVs) constitutes an isotropic expansion of Clinical Target Volumes as per International Commission of Radiation Units (ICRU) reports 50, 62 and 83. PTVs are not edited.

The size of the margin will vary amongst treating institutions, reflecting the degree of geometric uncertainty present in their planning process, but will typically be 3 - 5mm.

For IMPT, whilst a PTV may be created to aid optimisation, plans will be evaluated under uncertainty.

6.3. Organs at Risk (OARs) delineation:

The descriptions below have been adopted from the Global Harmonization Group organ at risk consensus contouring guidelines[12][REF]. The brachial plexus (IMPT patients only) will be delineated according to Hall et al[8] and the ADSCaN trial OAR atlas[9]. Table 4 details the normal tissue structures that should be contoured.

Table 4. Organs at Risk (OARS) for delineation

Nomenclature	Descriptor
SpinalCord	Outline the true spinal cord (not the spinal canal) from the tip of the dens of C2 (cranial border) to the upper edge of T3 (caudal border)
SpinalCord_PRVX (where X = margin)	An isotropic expansion of 3-5mm (according to local practice and adequacy of immobilisation)
Brainstem	Outline the entire brainstem from the substantia nigra at the cerebral peduncle (cranial border; the cranial aspect of the posterior clinoid process may be used as a bony landmark) to the level of the tip of the dens of the C2 vertebra (caudal border)
Brainstem_PRVX (where X = margin)	An isotropic expansion of 3-5mm (according to local practice and adequacy of immobilisation)
Parotid_R and Parotid_L	The anatomic boundaries of the parotid gland are as follows:<br><br>• Cranial – zygomatic arch<br>• Caudal – angle of mandible<br>• Anterior - masseter muscle<br>• Posterior - anterior aspect of sternocleidomastoid muscle<br>• Lateral - platysma muscle<br>• Medial - posterior belly of digastric muscle, styloid process, parapharyngeal space<br><br>Within the tissue of the parotid gland, include the carotid artery, retromandibular vein and extracranial facial nerve
Glnd_Submand_R and Glnd_Submand_L	The anatomic boundaries of the submandibular gland are as follows:<br><br>• Cranial - caudal edge of medial pterygoid muscle at the level of the C3 vertebral body<br>• Caudal - fatty tissue<br>• Lateral - platysma muscle, mandibular surface<br>• Medial - lateral surface of mylohyoid muscle, anterior belly of digastric muscle
Cavity_Oral	Posterior to mandible and maxilla and not to include the inner surface of the lips; the anatomic boundaries are as

	follows:<br><br>• Cranial – include hard palate mucosa, mucosal reflections near maxilla<br>• Caudal – base of tongue mucosa and hyoid posteriorly, include mylohyoid muscle and anterior belly of the digastric muscle anteriorly<br>• Posterior – include posterior borders of soft palate, uvula and (more inferiorly) the base of tongue<br>• Antero-lateral – inner surface of the mandible and maxilla
Glottis	Includes the vocal cords and paraglottic fat; the anatomic boundaries are as follows:<br><br>• Cranial – cranial edge of arytenoid cartilages<br>• Caudal – caudal edge of anterior part of thyroid cartilage<br>• Posterior – cricoid, anterior border of arytenoids<br>• Antero-lateral – thyroid cartilage<br>• Medial – pharyngeal lumen (exclude air)
Larynx_SG	The supraglottic larynx is a soft tissue structure including the epiglottis, supraglottic adductor muscles, aryepiglottic folds, arytenoid cartilages and false vocal cords. The anatomic boundaries are as follows:<br><br>• Cranial – include tip of epiglottis<br>• Caudal – cranial edge of arytenoid cartilages<br>• Anterior – hyoid bone, pre-epiglottic space, thyroid cartilage<br>• Posterior – inferior pharyngeal constrictor muscle, pharyngeal lumen<br>• Lateral – thyroid cartilage<br>• Medial – pharyngeal lumen (exclude air)
Musc_Constrict_S	The anatomic boundaries of the superior pharyngeal constrictor muscles are as follows:<br><br>• Cranial – caudal tip of the pterygoid plates (pterygoid hamulus)<br>• Caudal – lower edge of C2 vertebral body<br>• Anterior – pterygoid hamulus, posterior end of mandible, base of tongue<br>• Posterior – prevertebral muscle<br>• Lateral – medial pterygoid muscle, para-pharyngeal space
Musc_Constrict_M	The anatomic boundaries of the middle pharyngeal constrictor muscles are as follows:

	• Cranial – upper edge of C3 vertebral body<br>• Caudal – lower edge of hyoid bone<br>• Anterior – tongue base, hyoid bone<br>• Posterior – prevertebral muscle
Musc_Constrict_I	The anatomic boundaries of the inferior pharyngeal constrictor muscles are as follows:<br><br>• Cranial – lower edge of hyoid bone<br>• Caudal – lower edge of arytenoid cartilages<br>• Anterior – posterior edge of thyroid cartilage<br>• Posterior – prevertebral muscle<br>• Lateral – superior horn of thyroid cartilage
Inlet_Cricophar	The cricopharyngeal inlet encompasses the cricopharyngeal muscle and the oesophageal inlet; the anatomic boundaries are as follows:<br><br>• Cranial - lower edge of arytenoid cartilages<br>• Caudal - 10mm caudal to lower edge of cricoid cartilage<br>• Anterior - posterior edge of the cricoid cartilages<br>• Posterior - prevertebral muscle<br>• Lateral - thyroid cartilage, fatty tissue, thyroid gland (include all muscle layers)
Oesophagus_S	The anatomic boundaries of the cervical oesophagus are as follows:<br><br>• Cranial – 10mm caudal to lower edge of cricoid cartilage<br>• Caudal – suprasternal notch
Fossa_Posterior	The posterior fossa includes the brainstem (as contoured previously) and cerebellum; the anatomic borders are as follows:<br><br>• Superior - tentorium<br>• Inferior - foramen magnum<br>• Anterior – anterior edge of brainstem<br>• Posterior – posterior extension of meninges to inner table of skull<br>• Lateral - Lateral extension of meninges around cerebellum
Bone_Mandible	The mandible is defined as the entire mandible bone from the temporo-mandibular junction to the symphysis menti and excludes teeth.<br><br>The use of CT bone windows is recommended.
Cochlea_R and Cochlea_L	The cochlea is embedded in the temporal bone, located lateral to the internal auditory meatus and is best visualised

	on CT bone windows. The cochleae appear as small curved or round lucencies in the petrous portion of the temporal bone<br><br>The cochleae lie caudal to the semicircular canals, lateral to the internal auditory meatus, anterior to the vestibular apparatus and medial to the middle ear. The structure is small and measures up to 0.6cc; exclude the semi-circular canals.
BrachialPlex_R and BrachialPlex_L	The brachial plexus should be contoured for IMPT patients only<br><br>The defined ipsilateral brachial plexus originates from the spinal nerves exiting the neural foramina on the involved side from around C5 to T2<br><br>For the purposes of this protocol, nerve roots from C5 to T1 inclusive will be contoured as well as the main trunk of the brachial plexus, using the subclavian and axillary vessels as a surrogate for identifying the location of the plexus<br><br>This neurovascular complex will be contoured starting proximally at C5 and following along the route of the subclavian artery ending after the neurovascular structures cross the 2nd rib

6.4. Example outlining case (applicable to both IMPT and IMRT)

Oropharynx, left base of tongue SCC, p16 positive – T2 N2a M0 (TNM7), T2 N1 M0 (TNM8)

Clinical information: A 56 year old man presenting with a 3 month history of odynophagia, and a 1 month history of left neck swelling. He is otherwise fit and well with no co-morbidity.

MRI neck: left tongue base mass measuring 23 x 14 x 16mm. Enlarged lymph node (25 x 32 x 22mm) in left level II.

FNA & cytology left level II node: suspicious for squamous cell carcinoma

Tongue base biopsy: poorly differentiated squamous cell carcinoma, p16 positive

Step 1 - outline primary tumour and nodal GTVs (GTVp and GTVn), as shown in figure 5 (both in orange).

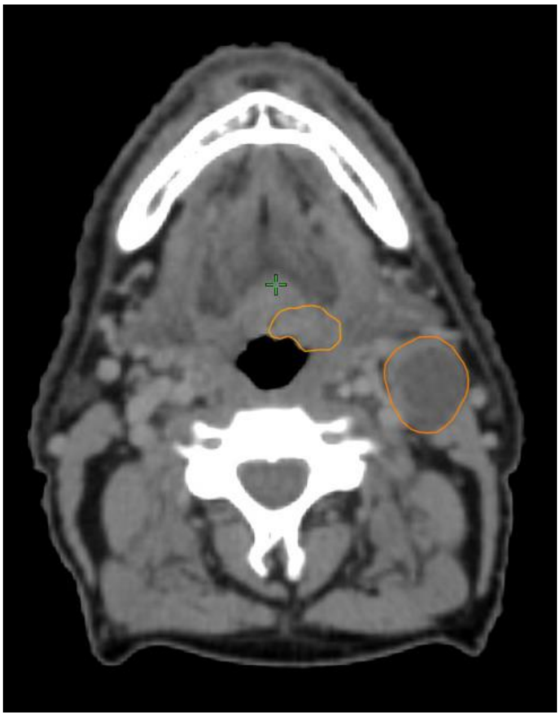

Figure 5

Step 2 - add a 5mm isotropic margin to the primary tumour GTV (GTVp) to create CTV1p, and a 10mm isotropic margin to the nodal GTV (GTVn) to create CTV1n (figure 6). Edit for air cavities, anatomical barriers to spread e.g. bone and fascia (including platysma) and combine to create the final CTV1, as shown in figure 7.

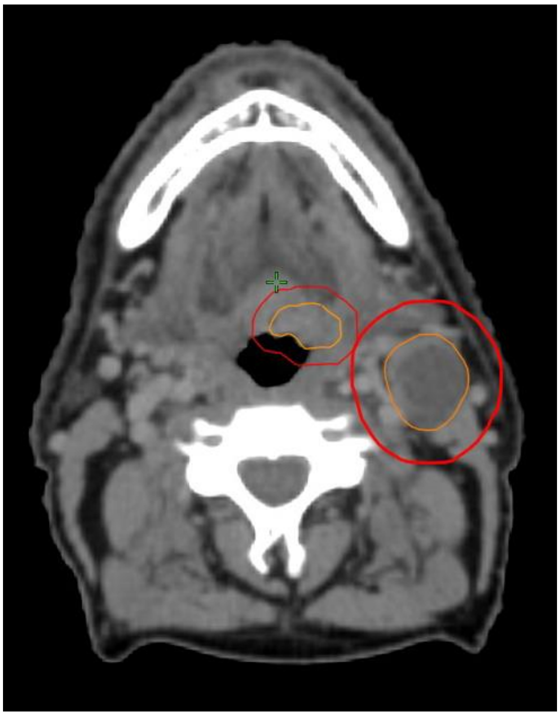

Figure 6

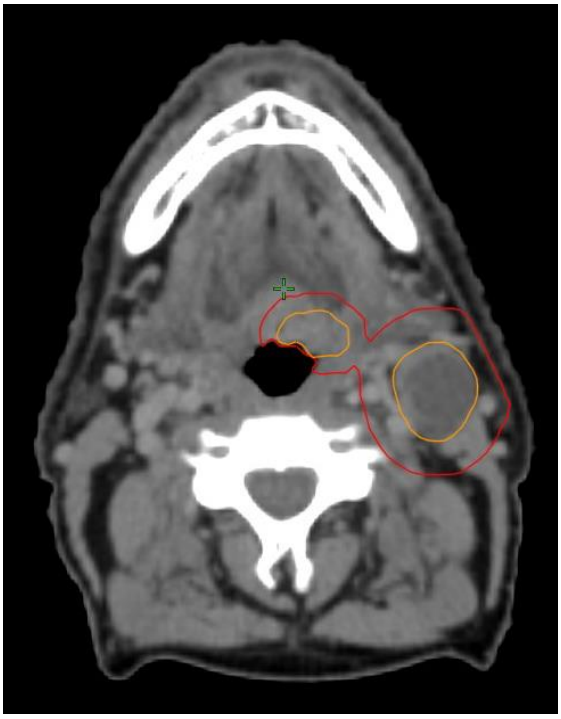

Figure 7

Step 3 – add a 10mm isotropic margin to GTVp to create CTV2p (figure 8). Edit for air cavities and anatomical barriers to spread e.g. bone, air, and fascia (including platysma), and ensure CTV2 does not overlap with CTV1n (figure 9).

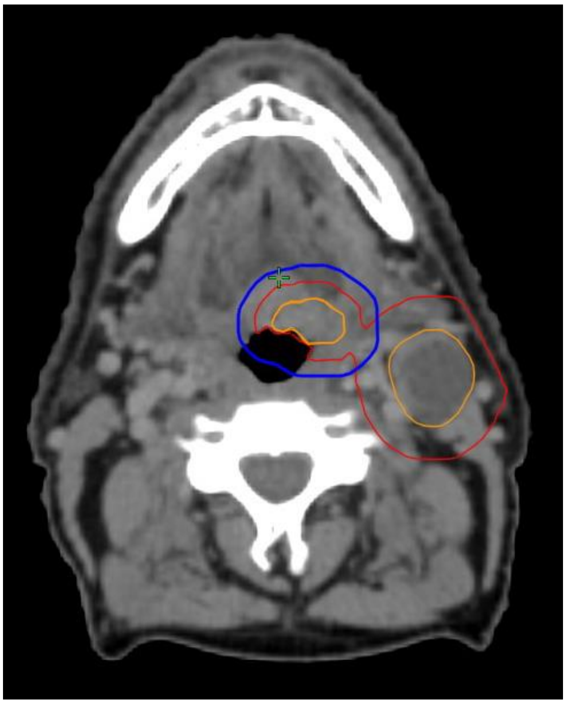

Figure 8

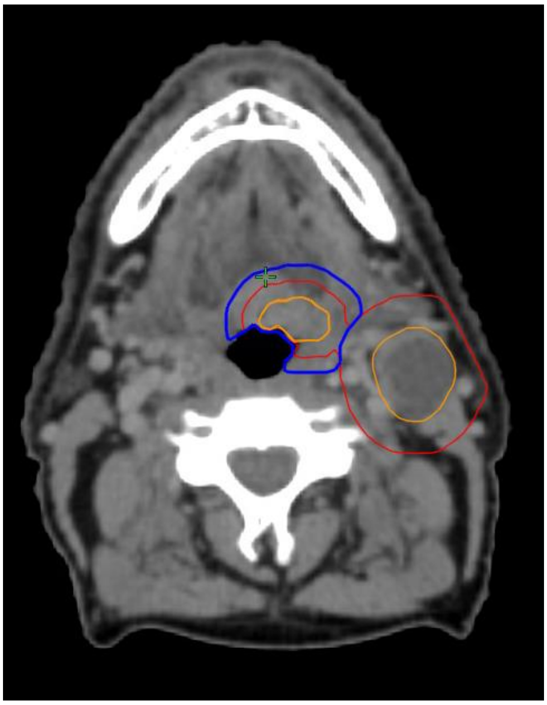

Figure 9

Outline the elective nodal volume (CTV2n) and combine with CTV2p to form the final CTV2 (figure 10).

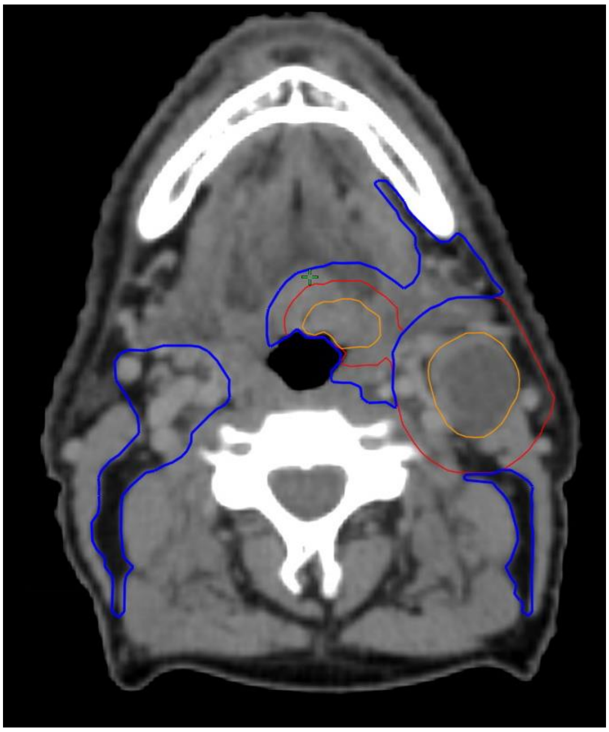

Figure 10

7. TREATMENT PLANNING

Planning aims should be prioritised according to Table 5.

Mandatory and optimal dose constraints for critical and other organs-at-risk are given in Tables 6 and 7 respectively. Target volume constraints are given in Table 8.

Plans will be prescribed to the mean (or median) of PlanCTV1 (IMPT) or PlanPTV1 (IMRT).

7.1. IMRT

Photon irradiation will be inverse planned IMRT or rotational arc therapy (e.g. Rapid Arc™, VMAT™ or Tomotherapy™). IMRT planning should follow ICRU 83.

Mandatory spinal cord and brainstem constraints are to the planning risk volume, which is typically a 3-5mm expansion. If these PRV overlap with PTVs, a PlanPTV (excluding a margin around the PRVs) can be created to aid optimisation, with PTV1 prioritised over PTV2. Target constraints are specified in table 6 for photon PTVs.

Density override volumes:

An additional structure should be created that covers all artefacts caused by dental fillings, as well as the high density regions where intravenous contrast had been injected. The density of the contoured are should be overridden to a bulk density of 0 HU.

Dose prescription and calculation:

The dose should be prescribed to the median PlanPTV rather than the unedited original volume, to avoid the low dose build-up unbalancing the overall dose.

Centres unable to prescribe to the median dose due to their planning system capabilities can alternatively prescribe to the mean dose and should inform the QA team of this decision. The median and mean dose should both be reported on the plan assessment form and are expected to be within 0.5 Gy of each other. Centres with any issues regarding the median/mean dose prescription should contact the QA team.

7.2. IMPT

Proton irradiation will be using pencil beam scanning. Multi-field optimisation is required due to complex target volumes, requiring field-specific targets to achieve adequate normal tissue sparing. It is desirable to cover each region of the target volumes with at least two fields.

The doses to targets and critical OARs shall be evaluated by recalculating plans in a number of error scenarios accounting for a setup error of 3-5mm and appropriate range uncertainty. If these volumes overlap with CTVs, a technical CTV volume (excluding a margin around OARs) can be created to aid optimisation, with CTV1 prioritised over CTV2. Target constraints are specified in Table 6 for proton CTVs, evaluated under setup and range uncertainty.

Beam angles will be chosen with consideration to:

- Non biological implants (particularly of unknown composition e.g. amalgam).
- CT artefacts.
- Regions of variable heterogeneity e.g. sinus.
- Avoiding entering through shoulders

- Multiple beams terminating at the same point on the brainstem, optics or spinal cord where RBE uncertainties can present an unaccounted for risk should be avoided.
- Reducing the volume of posterior normal tissue irradiated.
  Where beams enter through the couch, the couch should be appropriately modelled in the treatment planning system.

Table 5: Planning priority order for photon and proton planning. The order differs due to treatment of level Ib (including the submandibular gland) on both sides of the neck for bilateral involved neck nodes

**Unilateral involved neck node planning priority**	**Bilateral involved neck node planning priority**
Critical structures – spinal cord, brainstem	Critical structures – spinal cord, brainstem
Target coverage CTV1/PTV1	Target coverage CTV1/PTV1
Target coverage CTV2/PTV2	Target coverage CTV2/PTV2
Contralateral parotid	Contralateral parotid
Oral Cavity	Oral Cavity
Contralateral submandibular gland	Superior and middle pharyngeal constrictors
Superior and middle pharyngeal constrictors	Larynx
Larynx	Ipsilateral parotid
Ipsilateral parotid	Cochlea
Cochlea	Contralateral submandibular gland
Posterior fossa	Posterior fossa
Ipsilateral submandibular gland	Ipsilateral submandibular gland

Table 6: Mandatory dose constraints for critical organs-at-risk. Constraints are applied to the PRV for IMRT plans or the worst case under uncertainty for IMPT plans.

**Organ-at-risk**	**Dose statistic**	**Mandatory dose constraint**
SpinalCord under uncertainty/PRV	1cc	<46 Gy(RBE)
	0.1cc	<48 Gy(RBE)
BrainStem under uncertainty/PRV	1cc	<54 Gy(RBE)
	0.1cc	<55 Gy(RBE)

Table 7: Optimal dose constraints for other organs-at-risk. Constraints are applied to the volume in the nominal scenario and not under uncertainty or to a PRV.

Organ-at-risk	Dose statistic	Optimal dose constraint
Parotid_R	Mean	Contralateral: <20 Gy(RBE)<br>Ipsilateral: ALARA
Parotid_L	Mean	
Glnd_Submand_R	Mean	Contralateral: <35 Gy(RBE)<br>Ipsilateral: N/A
Glnd_Submand_L	Mean	
Cavity_Oral	Mean	<30 Gy(RBE)
Glottis	Mean	<45 Gy(RBE)
Larynx_SG	Mean	<45 Gy(RBE)
Musc_Constrict_S	Mean	<50 Gy(RBE)
Musc_Constrict_M	Mean	<50 Gy(RBE)
Musc_Constrict_I	Mean	<20 Gy(RBE)
Inlet_Cricophar	Mean	<20 Gy(RBE)
Oesophagus_S	Mean	<20 Gy(RBE)
Bone_Mandible	0.1cc	<73.5 Gy(RBE)
Cochlea_R	Mean	<30 Gy(RBE)
Cochlea_L	Mean	<30 Gy(RBE)
Fossa_Posterior	Mean	As low as possible
BrachialPlex_R*	0.1cc	60 Gy(RBE)
BrachialPlex_L*	0.1cc	60 Gy(RBE)

*IMPT plans only – on side(s) of neck with involved nodes

Table 8: Target volume objectives. For IMRT the target volume is the PTV and for IMPT the CTV is assessed under setup and range uncertainty.

	Dose (%)	
Volume (%)	Radical CTV*/PTV†	Elective CTV*/PTV†
99	≥90	≥90
95	≥95	≥95
50	100	-
5	≤105	As low as possible
2	≤107	As low as possible

* IMPT plans evaluated under setup and range uncertainty, † IMRT plans

8. VERIFICATION AND REPLANNING

Volume delineation, planning and treatment will take place locally (IMRT) or at The Christie or UCLH (IMPT).

Local practice should be followed. For IMPT this will be daily CBCT. For IMRT, i It is recommended that cone beam CT (CBCT) images should be acquired for the initial three fractions and assessed off-line, with weekly images taken thereafter. If a weekly image has an out of tolerance error, verification images should be repeated for the next 2 treatments to allow for calculation of a new systematic shift. Any systematic correction applied should be confirmed using imaging after being first applied.

Patients will have a repeat planning CT scan during week 3 (IMPT and IMRT), ideally on the Wednesday (#13). Please contact the RTTQA group if the mid-treatment CT needs to be delayed for any reason or if the patient has required re-scanning prior to week 3.

The mid-treatment scan will be co-registered with the initial planning CT scan and volume delineation contours transferred from initial planning CT scan to the verification CT scan for review by the local Clinical Oncologist.

Following review and where necessary, only OAR and elective nodal contours will be amended. The therapeutic target volume contours (i.e. the geometric expansions of 5+5mm or 10mm around the primary or nodal disease) are not amended/adapted to a change in GTV. Doses to targets, electively treated volumes and OARs will be re-calculated based on the original plan and coverage of target volumes reassessed. This is done assuming that the entire treatment will be delivered as per the verification scan

A replan is required if:

- The target coverage doesn't meet the D99% and D95% protocol criteria and on visual review target coverage is deemed not to be clinically acceptable.
- The cumulative maximum dose to critical structures is >2% over tolerance or less as per local centre protocol.
- The mean dose to parotids/oral cavity/submandibular glands have increased by >3 Gy(RBE) assuming the entire treatment will be delivered as per the verification scan.

A threshold of 3 Gy(RBE) was selected as corresponding to a difference of up to 10% in NTCP for the mean dose model in the linear range (25 Gy(RBE) to 55 Gy(RBE))[10]. Patient-reported dryness is improved by oral cavity and submandibular gland sparing, and by extrapolation a re-planning threshold of delta 3 Gy(RBE) will be applied.

If replanning is required then the patient should begin on the new plan as soon as possible, ideally on or before the Monday of week 5. Changes in patient anatomy and setup will be monitored during treatment with re-plans permitted where required. The reasons for replanning will be collected.

If, due to observed changes in on-line imaging, a patient is rescanned earlier than week 3, and week 3 on-line imaging is consistent a further rescan may not be required.

9. TREATMENT INTERRUPTIONS

Patients are treated as Category 1, defined by The Royal College of Radiologists[11]. Gaps in treatment should be compensated where deemed clinically appropriate to ensure treatment is delivered within the same overall treatment time (6.5 weeks). This should be by treatment with 2 fractions per day with an inter-fraction interval of at least 6 hours or patients may be treated on a weekend day (Saturday or Sunday). In this situation, patients should receive a maximum of six fractions per week.

Patients should not have a break in treatment where possible. Where interruptions are due to breakdown or unavailability of the treatment machine, patients should be transferred to a matched treatment machine in the first instance. Other causes of treatment interruption may be mitigated by treatment on weekends and bank holidays.

For proton patients, in the event of cyclotron downtime that cannot be mitigated by weekend treatment, patients will be prioritised to receive a photon backup plan according to standard departmental policy.

An IMRT contingency plan will be prospectively generated for each patient receiving IMPT so that in case of breakdown, patients will receive IMRT until service restored ensuring no treatment gaps. If used, these plans will be retrospectively reviewed for quality assurance.

10. RADIOTHERAPY QUALITY ASSURANCE

Radiotherapy Quality Assurance Overview

The radiotherapy quality assurance (RT QA) programme for the TORPEdO trial will be designed and implemented by the National Radiotherapy Trials QA (RTTQA) Group.

This will include pre-trial and on-trial components.

The full details of the programme will be made available on the RTTQA group website (www.rttrialsqa.org.uk).

RT QA streamlining

The QA processes for the TORPEdO trial will be streamlined, where possible, with previous head and neck trial QA based on both centre and Principal Investigator (PI) trial participation.

Target Volume Outlining: Principal Investigators who have successfully completed the TV outlining QA for DARS, NIMRAD, PATHOS and CompARE will be streamlined.

OAR Outlining: Principal Investigators who have successfully completed the OAR outlining QA for DARS, NIMRAD, PATHOS, CompARE and WISTERIA will be streamlined where applicable.

Planning: Centres who have successfully completed the IMRT benchmark bilateral planning case for DARS, NIMRAD, PATHOS, CompARE, WISTERIA, will be streamlined.

Please contact the RTTQA group on the TORPEdO QA email TORPEdOqa.enh-tr@nhs.net for clarification.

Pre-Trial QA

All Pre-Trial QA is completed prior to centre activation for the trial and may include:

Facility Questionnaire (FQ) - General and trial specific questions on equipment, software, techniques and procedures to be used for the trial.

Benchmark Outlining and Planning Cases - QA of the outlining and planning technique.

Dosimetry Audit - Centres must have successfully completed an independent external dosimetry audit through the RTTQA group or other external group.

On trial QA

Prospective case review: The outlining of all cases will be prospectively reviewed. At least the first IMRT and IMPT plans from each centre will be reviewed .

Centres will be notified by email when a patient requires a prospective review.

Further prospective and/or timely retrospective reviews may be deemed necessary at the discretion of the RTTQA group and the trial CI.

To facilitate prospective case review all data should be submitted to the RTTQA Group as soon as it has been completed (case review turnaround time is a maximum of 72 hours). Please send the outlining as soon as it has been completed, together with the case history and associated imaging, to allow review before planning commences. Please allow sufficient time between plan submission and treatment start date for amendments to be actioned.

Retrospective case review:

- All IMRT and IMPT plans will be retrospectively reviewed.
- All IMPT patients will have an contingency pathway. If the patient receives treatment on an IMRT plan, this will be retrospectively reviewed.

- All rescan or replan data should be submitted for timely retrospective review.
- The mid-treatment planning CT should also be submitted for retrospective review.

Universal data collection

Anonymised data, in DICOM format, will be collected for all patients. This will include:

- Brief clinical history
- Diagnostic imaging reports (CT, MRI, PET-CT)
- Diagnostic and/or planning MRIs
- Planning CT
- Structure set
- RT plan
- Total dose cube
- Per Beam dose cubes (IMPT only)
- Rescans (inc. mid-treatment planning CT) and replan data
- All CBCTs

Please send all anonymised data to the RTTQA contact.

Please email the RTTQA contact on the TORPEdO QA email TORPEdOqa.enh-tr@nhs.net with any queries.

11. OUTLINING BENCHMARK CASE

One case should be contoured per radiotherapy centre:

- This should be undertaken by the local investigator who will be responsible for radiotherapy treatment, in collaboration with the local team.
- Please contour according to the TORPEdO Radiotherapy Guideline Document,
- Please adhere to the nomenclature as per the TORPEdO Radiotherapy Guideline Document

Case History – oropharynx, left base of tongue SCC, p16 positive – T2 N2b M0 (TNM7), T2 N1 M0 (TNM8)

Clinical history:

54 year old man, 3 month history of left neck mass.

No significant past medical history. No regular medications. Life-long never smoker, drinks 10 units of alcohol per week. Works as a builder. Performance status 0.

Examination findings:

No abnormality seen on examination of oral cavity.

On nasendoscopy, lesion seen at left base of tongue, does not cross midline.

Palpable left level II node, measures approximately 4.5 x 4.5cm, mobile.

Left tongue base biopsy:

Basaloid squamous cell carcinoma, diffusely and strongly positive for p16.

MRI neck:

There is a soft tissue mass involving the left tongue base that just abuts the margin of hyoglossus muscle and extends to the vallecula. The lesion measures approximately 18mm (AP) x 22mm (RL) x 24mm (CC).

There is a large, septated, predominately cystic lesion with the left level II, which measures approximately 42mm (CC height). There is a smaller partly necrotic node just inferiorly within left level III. There are no features to suggest macroscopic extracapsular spread.

CT thorax:

No evidence of lung metastases.

- Primary tumour and nodal GTVs are already completed
- Clinical target volumes and OARs to be contoured

12. PLANNING BENCHMARK CASE

The outlining benchmark case (above) with gold standard reference contours will be used as the planning benchmark case.

Please plan in accordance with the TORPEdO Radiotherapy Guideline Document.

- Please import the CT and structure sets for the **TORPEdO Planning Case** into your own TPS system.

- Plan this case as you expect to do for patients on the trial (e.g. the same treatment planning system, beam model, standard beam/arc arrangements, etc.).

- This case is pre-contoured with:

    - Target volumes - GTV, CTV1, CTV2, PTV1, PTV2, PlanPTV1, PlanPTV2, PlanCTV1, PlanCTV2
    - OARs – SpinalCord, Brainstem, Parotid_R, Parotid_L, Glnd_Submand_R, Glnd_Submand_L, Cavity_Oral, Glottis, Larynx_SG, Musc_Constrict_I, Musc_Constrict_S, Inlet_Cricophar, Oesophagus_S, Fossa_Posterior, Bone_Mandible, Cochlea_R, Cochlea_L, BrachialPlex_R, BrachialPlex_L

- Please do not alter any of these contours (except as detailed below). Create additional contours as needed to aid planning.

- If you need to modify the Body outline for dose calculation or optimisation, please copy and rename the structure (keeping the original) and inform the QA team when submitting your plans.

- Grow the PTVs and PRVs using your locally-determined margins (if these don't match what was included in the benchmark case). The margins should be based on an assessment of the immobilisation and setup used locally.

- Where proton planning a photon back up plan should be produced

13. REFERENCES

1. www.aboutcancer.com, Fauces – medial sagittal view, aboutcancer.com
2. www.aboutcancer.com, anatomy of the pharynx (posterior view), aboutcancer.com
3. Gregoire V, Ang K, Budach W, Grau C, Hamoir M, Langendijk JA, Lee A, Le QT, Maingon P, Nutting C, O'Sullivan B, Porceddu SV, and Lengele B. *Delineation of the neck node levels for head and neck tumors: A 2013 update. DAHANCA, EORTC, HKNPCSG, NCIC CTG, NCRI, RTOG, TROG consensus guidelines*. Radiother Oncol, 2014. 110(1): p. 172-181.
4. Gregoire V, Evans M, Le QT, Bourhis J, Budach V, Chen A, Eisbruch A, Feng M, Giralt J, Gupta T, Hamoir M, Helito JK, Hu C, Hunter K, Johansen J, Kaanders J, Laskar SG, Lee A, Maingon P, Makitie A, Micciche F, Nicolai P, O'Sullivan B, Poitevin A, Porceddu S, Skladowski K, Tribius S, Waldron J, Wee J, Yao M, Yom SS, Zimmermann F, and Grau C. *Delineation of the primary tumour Clinical Target Volumes (CTV-P) in laryngeal, hypopharyngeal, oropharyngeal and oral cavity squamous cell carcinoma: AIRO, CACA, DAHANCA, EORTC, GEORCC, GORTEC, HKNPCSG, HNCIG, IAG-KHT, LPRHHT, NCIC CTG, NCRI, NRG Oncology, PHNS, SBRT, SOMERA, SRO, SSHNO, TROG consensus guidelines*. Radiother Oncol, 2018. 126(1): p. 3-24
5. Eisbruch A, Marsh LH, Dawson LA et al. *Recurrences near base of skull after IMRT for head-and-neck cancer: implications for target delineation in high neck and for parotid gland sparing*. Int. J. Radiation Oncology Biol Phys 2004. 59(1): p. 28-42.
6. Brouwer CL, Steenbakkers RJ, Bourhis J, Budach W, Grau C, Gregoire V, van Herk M, Lee A, Maingon P, Nutting C, O'Sullivan B, Porceddu SV, Rosenthal DI, Sijtsema NM, and Langendijk JA. *CT-based delineation of organs at risk in the head and neck region: DAHANCA, EORTC, GORTEC, HKNPCSG, NCIC CTG, NCRI, NRG Oncology and TROG consensus guidelines*. Radiother Oncol, 2015. 117(1): p. 83-90
7. Christianen MEMC, Langendijk JA, Westerlaan HE, van de Water TA, Bijl HP. *Delineation of organs at risk involved in swallowing for radiotherapy treatment planning*. Radiother Oncol, 2011. 101(3): p. 394-402.
8. Hall WH, Guiou M, Lee NY, Dublin A, Narayan S, Vijayakumar S, Purdy JA, Chen AM. *Development and validation of a standardized method for contouring the brachial plexus: preliminary dosimetric analysis among patients treated with IMRT for head-and-neck cancer.* Int. J. Radiation Oncology Biol Phys 2008. 72(5): p. 1362-1367.
9. Isotoxic IMRTstudy TMG. OAR Atlas for the ADSCaN Study: All trial arms. p. 1-13.
10. Brouwer CL, Steenbakkers RJ, van der Schaaf A, Sopacua CT, van Dijk LV, Kierkels RG, Bijl HP, Burgerhof JG, Langendijk JA, and Sijtsema NM. *Selection of head and neck cancer patients for adaptive radiotherapy to decrease xerostomia.* Radiother Oncol, 2016. 120(1): p. 36-40.
11. The Royal College of Radiologists*: The timely delivery of radical radiotherapy: guidelines for the management of unscheduled treatment interruptions* 2019. Fourth Edition.
12. Mir R, Kelly SM, Xiao Y, et al. Organ at risk delineation for radiation therapy clinical trials: Global Harmonization Group consensus guidelines. Radiother Oncol. 2020 Sep;150:30-39. doi: 10.1016/j.radonc.2020.05.038. Epub 2020 Jun 3. PMID: 32504762.
