# Supplementary material for: TORPEdO: A phase III trial of intensity-modulated proton beam therapy versus intensity-modulated radiotherapy for multi-toxicity reduction in oropharyngeal cancer
Source: Clin Transl Radiat Oncol. 2022 Nov 21;38:147–54. doi: 10.1016/j.ctro.2022.11.010 (PMC9702982; doi:10.1016/j.ctro.2022.11.010)
Supplement: Supplementary data 3 [file mmc3.docx]

**TORPEdO Trial Management Group**

David Thomson The Christie NHS Foundation Trust

Russell Banner Swansea Bay University Health Board

Matthew Beasley University Hospitals Bristol NHS Foundation Trust

Guy Betts Manchester University NHS Foundation Trust

Katie Biscombe The Institute of Cancer Research

Helen Bulbeck Brainstrust

Frances Charlwood The Christie NHS Foundation Trust

Matt Clarke The Christie NHS Foundation Trust

Clare Cruickshank The Institute of Cancer Research

Olly Donnelly Portsmouth Hospitals NHS Trust

Deborah Gardiner The Institute of Cancer Research

Callum Gillies University College London Hospitals NHS Foundation Trust

James Good University Hospitals Birmingham NHS Foundation Trust

Clare Griffin The Institute of Cancer Research

Emma Hall The Institute of Cancer Research

Virag Harmath The Institute of Cancer Research

Jarrod Homer The Christie NHS Foundation Trust

Sabina Khan University College London Hospitals NHS Foundation Trust

Johannes Langendijk University Medical Centre Groningen

Lip Wai Lee The Christie NHS Foundation Trust

Jim Lester Sheffield Teaching Hospitals NHS Foundation Trust

Zoe Lingard The University of Manchester

Mat Lowe The Christie NHS Foundation Trust

Chris Nutting The Institute of Cancer Research /Royal Marsden NHS Foundation Trust

Abdullahi Omar The Institute of Cancer Research

Nachi Palaniappan Velindre NHS Trust

Julian Phillips RTTQA, Mount Vernon Hospital

Robin Prestwich Leeds Teaching Hospitals NHS Trust

James Price The Christie NHS Foundation Trust

Clare Roberts The Christie NHS Foundation Trust

Justin Roe Royal Marsden NHS Foundation Trust/Imperial College, London.

Ramkumar Shanmugasundaram University Hospital Southampton NHS Trust

Anna Thompson University College London Hospitals NHS Foundation Trust

Justine Tyler RTTQA, Mount Vernon Hospital

Marcel Van Herk The Christie NHS Foundation Trust

Catharine West The University of Manchester

Lorna Wilson The Christie NHS Foundation Trust

Jane Wolstenholme University of Oxford

**TORPEdO Protocol Development Group**

David Thomson The Christie NHS Foundation Trust

Matthew Beasley University Hospitals Bristol NHS Foundation Trust

Helen Bulbeck Patient Involvement Advisor - Brainstrust

Frances Charlwood The Christie NHS Foundation Trust

Matt Clarke The Christie NHS Foundation Trust

Olly Donnelly Portsmouth Hospitals NHS Trust

Marie Emson The Institute of Cancer Research

Bernadette Foran Sheffield Teaching Hospitals NHS Foundation Trust.

Callum Gillies University College Hospitals London NHS Foundation Trust

James Good University Hospitals Birmingham NHS Foundation Trust.

Emma Hall The Institute of Cancer Research

Johannes Langendijk University Medical Centre Groningen

Lip Wai Lee The Christie NHS Foundation Trust

Mat Lowe The Christie NHS Foundation Trust

Andrew McPartlin The Christie NHS Foundation Trust

Elizabeth Miles Mount Vernon Hospital

Mercy Ofuya The Institute of Cancer Research

Christopher Nutting The Institute of Cancer Research/ Royal Marsden NHS Foundation Trust

Nachi Palaniappan Velindre NHS Trust

Robin Prestwich The Leeds Teaching Hospitals NHS Trust.

James Price The Christie NHS Foundation Trust

Clare Roberts The Christie NHS Foundation Trust

Justin Roe Royal Marsden NHS Foundation Trust/Imperial College, London.

Joel Smith University of Oxford

Anna Thompson University College Hospitals London NHS Foundation Trust

Catharine West The University of Manchester

Lorna Wilson The Christie NHS Foundation Trust
